# A cohort-based study of host gene expression: tumor suppressor and innate immune/inflammatory pathways associated with the HIV reservoir size

**DOI:** 10.1371/journal.ppat.1011114

**Published:** 2023-11-29

**Authors:** Ashok K. Dwivedi, Germán G. Gornalusse, David A. Siegel, Alton Barbehenn, Cassandra Thanh, Rebecca Hoh, Kristen S. Hobbs, Tony Pan, Erica A. Gibson, Jeffrey Martin, Frederick Hecht, Christopher Pilcher, Jeffrey Milush, Michael P. Busch, Mars Stone, Meei-Li Huang, Julieta Reppetti, Phuong M. Vo, Claire N. Levy, Pavitra Roychoudhury, Keith R. Jerome, Florian Hladik, Timothy J. Henrich, Steven G. Deeks, Sulggi A. Lee

**Affiliations:** 1 Department of Medicine, Division of HIV, Infectious Diseases & Global Medicine, University of California, San Francisco, California, United States of America; 2 Vaccine and Infectious Disease Division, Fred Hutchinson Cancer Center, Seattle, Washington, United States of America; 3 Department of Obstetrics and Gynecology, University of Washington, Seattle, Washington, United States of America; 4 Department of Medicine, Division of Experimental Medicine, University of California San Francisco, California, United States of America; 5 Department of Biostatistics & Epidemiology, University of California San Francisco, California, United States of America; 6 Vitalant Blood Bank, San Francisco, California, United States of America; 7 Department of Laboratory Medicine and Pathology, University of Washington, Seattle, Washington, United States of America; 8 Universidad de Buenos Aires (UBA), Consejo Nacional de Investigaciones Científicas y Técnicas (CONICET), Instituto de Fisiología y Biofísica Bernardo Houssay (IFIBIO- Houssay), Buenos Aires, Argentina; 9 Department of Medicine, Division of Allergy and Infectious Diseases, University of Washington, Seattle, Washington, United States of America; Emory University, UNITED STATES

## Abstract

The major barrier to an HIV cure is the HIV reservoir: latently-infected cells that persist despite effective antiretroviral therapy (ART). There have been few cohort-based studies evaluating host genomic or transcriptomic predictors of the HIV reservoir. We performed host RNA sequencing and HIV reservoir quantification (total DNA [tDNA], unspliced RNA [usRNA], intact DNA) from peripheral CD4+ T cells from 191 ART-suppressed people with HIV (PWH). After adjusting for nadir CD4+ count, timing of ART initiation, and genetic ancestry, we identified two host genes for which higher expression was significantly associated with smaller total DNA viral reservoir size, *P3H3* and *NBL1*, both known tumor suppressor genes. We then identified 17 host genes for which lower expression was associated with higher residual transcription (HIV usRNA). These included novel associations with membrane channel (*KCNJ2*, *GJB2*), inflammasome (*IL1A*, *CSF3*, *TNFAIP5*, *TNFAIP6*, *TNFAIP9*, *CXCL3*, *CXCL10*), and innate immunity (*TLR7*) genes (FDR-adjusted q<0.05). Gene set enrichment analyses further identified significant associations of HIV usRNA with TLR4/microbial translocation (q = 0.006), IL-1/NRLP3 inflammasome (q = 0.008), and IL-10 (q = 0.037) signaling. Protein validation assays using ELISA and multiplex cytokine assays supported these observed inverse host gene correlations, with P3H3, IL-10, and TNF-α protein associations achieving statistical significance (p<0.05). Plasma IL-10 was also significantly inversely associated with HIV DNA (p = 0.016). HIV intact DNA was not associated with differential host gene expression, although this may have been due to a large number of undetectable values in our study. To our knowledge, this is the largest host transcriptomic study of the HIV reservoir. Our findings suggest that host gene expression may vary in response to the transcriptionally active reservoir and that changes in cellular proliferation genes may influence the size of the HIV reservoir. These findings add important data to the limited host genetic HIV reservoir studies to date.

## Author summary

Although lifelong HIV antiretroviral therapy (ART) suppresses virus, the major barrier to an HIV cure is the persistence of infected cells, “the HIV reservoir.” There are limited host genomic HIV reservoir studies to date. We performed a large cross-sectional study of 191 people with HIV on ART and measured the HIV reservoir size and host gene expression (RNA-seq) from blood CD4+ T cells. We found that individuals with higher expression of host genes involved in suppressing cell proliferation (*P3H3*, *NBL1*) had a smaller total HIV reservoir size. We also observed that individuals with more “transcriptionally active” HIV reservoir had decreased expression of inflammatory signaling (e.g., IL-1β, TLR7, TNF-α, IL-10) genes as well as two membrane channel proteins (encoded by *KCNJ2*, *GJB2*). While we were able to validate some of these findings at the protein level (P3H3, IL-10, TNF-α), further studies are needed to confirm these findings in larger cohorts with longitudinal sampling, including traditionally underrepresented populations in HIV cure research.

## Introduction

Despite several unique cases of possible HIV remission [1–3], there is still no HIV vaccine or cure. The major barrier to a cure is the persistence of infected cells during effective antiretroviral therapy (ART). Modern ART has transformed HIV disease into a treatable chronic disease for individuals who have access to, and are able to maintain, viral suppression [4]. However, ART alone does not eliminate persistent virus in most individuals [5,6]. HIV cure trials aimed at reactivating and eliminating the HIV reservoir have thus far failed to show a clinically meaningful reduction in the HIV reservoir [7–12]. There is an urgent need to bridge drug discovery with a deeper understanding of host-viral dynamics. Although several host factors have been shown to influence the size of the “HIV reservoir”, such as the timing of ART initiation after initial HIV infection [13–16], pre-ART viral load [17], ethnicity [17], and sex [17–20], there are few published human genomic and transcriptomic epidemiologic studies describing potential host factors influencing HIV persistence during treated infection.

Prior host genome wide association studies (GWAS) have focused on predictors of viral control (during untreated HIV disease), identifying key mutations in the C-C chemokine receptor type 5 gene (*CCR5Δ32*) and the human Major Histocompatibility Complex (MHC) human leukocyte antigen (HLA)-B and -C regions, that influence viral setpoint [21–24]. Recently our group reported these mutations (CCR5Δ32 and HLA -B*57:*01)* are associated with smaller HIV reservoir size [25]. However, mRNA expression from DNA variation is complex and not strictly 1:1 DNA to RNA transcription; gene expression is influenced by various factors (alternative splicing, polyadenylation, regulatory enhancers, epigenetic changes, etc.) which may differ by cell type and tissue [26–28]. The limited number of host gene expression studies during HIV infection (e.g., RNA sequencing) have compared gene expression between distinct clinical HIV groups. For example, one prior study compared gene expression among HIV “controllers” (individuals able to control virus in the absence of therapy) versus “non-controllers” [29]. Another study compared HIV non-controllers initiating ART “early” (<6 months from HIV infection) versus “later” (≥6 months after infection) [30]. However, no epidemiologic study has examined quantitative measures of the HIV reservoir size in relation to differences in host gene expression.

**Fig 1:**
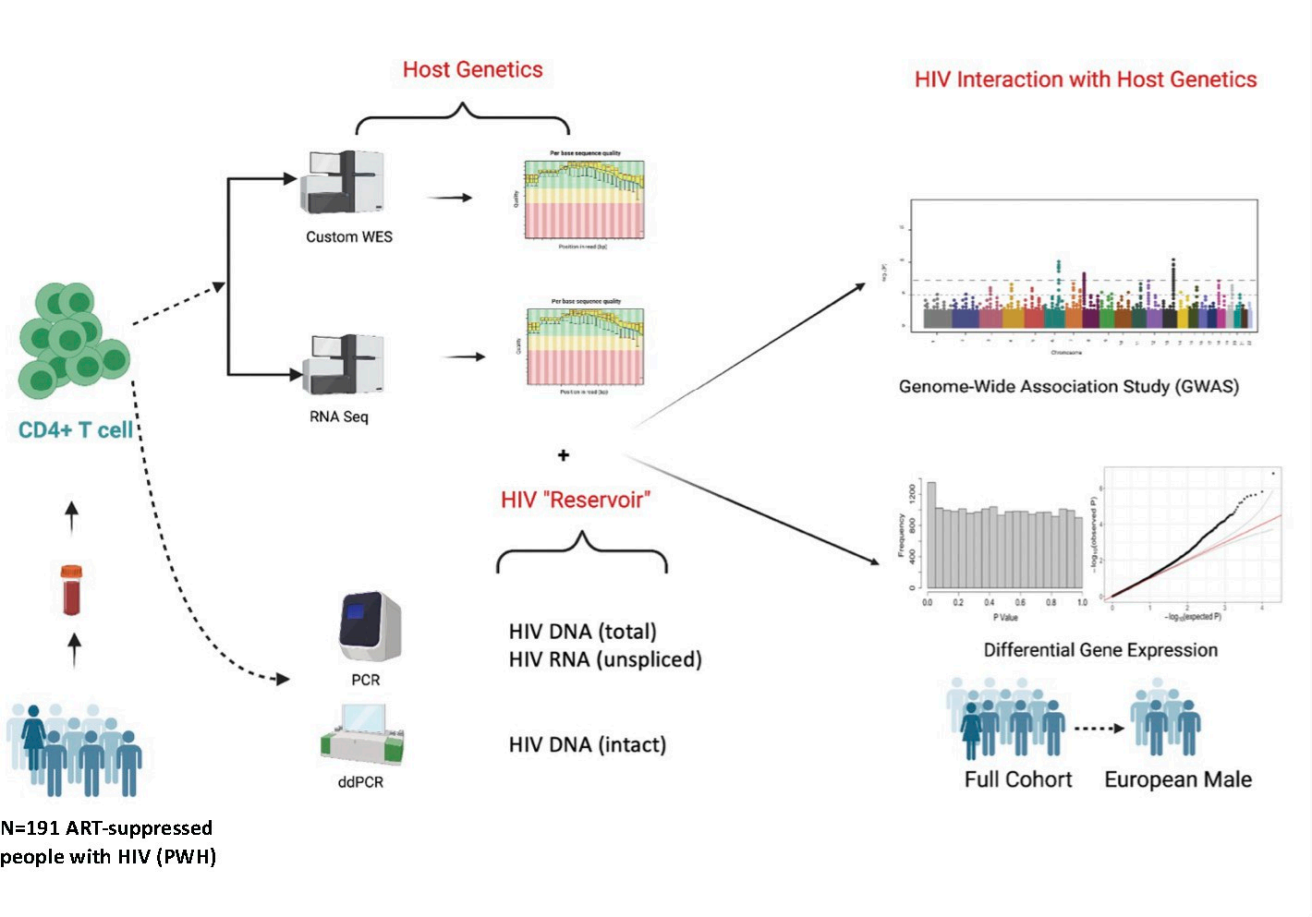
Study methods. DNA and RNA were extracted from CD4+ T cells enriched from cryopreserved peripheral blood mononuclear cells (PBMCs) from 191 ART-suppressed people with HIV (PWH). Extracted DNA was used to perform HIV reservoir quantification (total DNA by quantitative PCR, intact DNA by digital droplet PCR) and host DNA exome sequencing [37]. Extracted RNA was used to perform HIV reservoir quantification (unspliced RNA by qPCR) and host bulk RNA sequencing for the current study. Created with BioRender.com.

Here, we performed a cross-sectional study of 191 ART-suppressed HIV+ non-controllers to identify differentially expressed host genes in relation to three measures of the peripheral CD4+ T cell reservoir: HIV cell-associated “intact” DNA (an estimate of the frequency of potentially “replication-competent” virus with intact HIV genomes) [31], total DNA (“tDNA,” which approximates the total reservoir size, the sum of intact DNA and defective DNA) and unspliced RNA (“HIV usRNA,” which reflects the “transcriptionally active” reservoir) (**Fig 1**). *NBL1* and *P3H3*, both encoding for tumor suppressor genes that inhibit cell proliferation, were the only two genes host genes significantly associated with HIV total DNA reservoir size; higher expression of these genes was associated with lower HIV total DNA. HIV usRNA was significantly inversely associated with several host genes involved in innate immune and inflammatory signaling, as well as with two genes encoding for membrane channel proteins involved in HIV-1 entry and cell-cell communication. Protein validation in a subset of participants with additional biospecimen availability demonstrated consistent inverse associations as observed in the RNA-seq for HIV total DNA (P3H3) and HIV usRNA (IL-10 and TNF-α). Further studies are needed to validate these findings, ideally with dedicated functional genomic and intracellular protein assays using longitudinal samples to demonstrate causality of these observed associations. Our findings add important clinical and immunologic data to the limited host genomic HIV reservoir studies to date.

## Results

### Study population

A total of 191 ART-suppressed participants were selected from the UCSF SCOPE and Options cohorts (**S1 Fig**). HIV “controllers” [32–34] were excluded (individuals with undetectable viral loads in the absence of therapy for ≥1 year). Estimated date of detected infection (EDDI) was calculated to determine recency of infection in relation to ART initiation using the Infection Dating Tool (https://tools.incidence-estimation.org/idt/) [35]. The study included individuals who initiated ART during early (within 6 months) and chronic (>6 months) HIV (**Table 1**). The median age of the cohort was 47 years, nadir CD4+ T cell count, 352 cells/mm^3^, pre-ART viral load, 5.1 log_10_copies/mL, and years of ART suppression, 5.1 years. Consistent with our San Francisco-based study population, participants were mostly male (96%) and reported diverse ethnicity, which was reflected in our principal component analysis (PCA) [36] based on our previously published host DNA exome sequencing data [37] (**S2 Fig**). PCs generated for each participant were included in all downstream multivariate models to adjust for potential confounding by genetic ancestry.

**Table 1:** Descriptive statistics for the study population of 191 HIV-infected ART-suppressed non-controllers.

Descriptive Characteristic	Total (N = 191)	Early-Treated^a^ (N = 54)	Later-Treated^a^ (N = 137)
Male (%)^b^	183 (96%)	54 (100%)	129 (94%)
Age (years)	47 (13)	44 (12)	47 (13)
Nadir CD4+ T cell count (cells/mm^3^)	352 (251)	522 (346)	304 (190)
Maximum pre-ART HIV RNA (log_10_copies/mL)	5.1 (0.9)	5.6 (0.7)	5.0 (0.8)
Duration of ART suppression (years)	5.1 (4.2)	6.0 (4.3)	4.7 (4.2)
Timing of ART initiation (years)	2.0 (4.6)	0.20 (0.19)	3.5 (4.3)
HIV intact DNA (log_10_copies/10^6^ CD4+ T cells)	1.3 (1.0)	1.3 (0.5)	1.9 (1.0)
HIV total DNA (log_10_copies/10^6^ CD4+ T cells)	1.0 (1.3)	0.4 (1.3)	1.2 (1.3)
HIV unspliced RNA (log_10_copies/10^6^ CD4+ T cells)	3.2 (0.8)	3.0 (0.8)	3.3 (0.7)
HIV RNA/DNA	2.3 (1.0)	2.4 (1.0)	2.3 (0.9)

^a^ Early-treated = Individuals who initiated ART within 6 months of the date of detected HIV infection; later-treated = Individuals who initiated ART after 6 months of date of detected HIV infection.

^b^ Absolute frequencies (with percent or median values).

### Measures of the HIV total DNA and unspliced RNA were correlated, but low levels of intact DNA were detected

CD4+ T cells from cryopreserved PBMCs were isolated by magnetic negative selection, and RNA was extracted for HIV reservoir quantification (usRNA) and host transcriptomics (RNA-seq) while DNA was extracted for HIV reservoir quantification (tDNA, intact DNA). Most of the HIV reservoir consists of cells harboring defective virus [38,39], while the “replication-competent” reservoir measures that HIV-infected cells harboring intact DNA, capable of producing infectious virions [31,40,41]. Currently, there is no “gold standard” for measuring the HIV reservoir [42,43]. Here, we measured HIV total DNA (tDNA), which approximates the total defective and intact proviral DNA reservoir, and HIV unspliced RNA (usRNA), which estimates the “transcriptionally active” HIV reservoir, using an in-house qPCR TaqMan assay [44]. Using the remaining extracted DNA from the CD4+ T cells, we performed a multiplexed ddPCR assay targeting three regions of the HIV-1 genome to quantify the frequency of cells with “intact” HIV (a proxy for the frequency of replication-competent provirus) [31]. Of the three measures that we performed to quantify the HIV reservoir, HIV total DNA and unspliced RNA were highly correlated with one another (both quantified using quantitative, qPCR), Spearman R = 0.55, p = 1.6x10^-17^ (**S3 Fig**). HIV intact DNA (performed as a separate droplet digital, ddPCR assay using remaining DNA samples) was significantly associated with HIV usRNA (Spearman R = 0.26, p = 5.0x10^-4^) but not HIV total DNA. This may have been due to unusually high proportion of our study population with undetectable values (48%) for HIV intact DNA (while HIV total DNA by qPCR was measurable in 95% of samples) as described further in the Discussion section.

### Known clinical predictors of the HIV reservoir size were associated with HIV total DNA, unspliced RNA, and intact DNA

Earlier timing of ART initiation and higher nadir CD4+ T cell count were associated with smaller HIV reservoir size in our cohort, consistent with prior reports [17,39,43]. Earlier timing of ART initiation (<6 months from infection) was significantly associated with lower levels of total DNA (Spearman R = 0.29; p = 2.3x10^-5^) and usRNA (Spearman R = 0.28; p = 4.2x10^-5^) and demonstrated a trend with HIV intact DNA (Spearman R = 0.14; p = 0.061) (**S4 Fig**). Lower nadir CD4+ T cell counts were associated with higher total HIV DNA (Spearman R = -0.26; p = 2.3x10^-4^), as well as with higher HIV usRNA (Spearman R = -0.30; p = 1.5x10^-5^) and HIV intact DNA (Spearman R = -0.27; p = 3.7x10^-4^) (**S5 Fig**). We did not observe a significant association with duration of ART suppression, age, or pre-ART viral load. Given the low frequency of females and transgender participants in our study, we were unable to formally compare results based on sex/gender, but sensitivity analyses suggested that inclusion of these participants did not change our overall findings and thus results are shown for the entire combined cohort.

### Individuals with higher expression of tumor suppressor genes (*P3H3*, *NBL1*) had smaller HIV total DNA reservoir size

Peripheral CD4+ T cells isolated by magnetic negative selection were subjected to bulk host mRNA sequencing. Differential gene expression analyses demonstrated that individuals with higher *NBL1* and *P3H3* gene expression had significantly lower HIV total DNA (measured by percent change in host gene expression per two-fold change in HIV total DNA; *NBL1*: -1.8%, q = 0.012; *P3H3*: -1.6%, q = 0.012) in multivariate models controlling for significant covariates, nadir CD4+ T cell count, timing of ART initiation, genetic ancestry (PCs [36,37]), and residual variability (PEERs [45]) (**Table 2**). *P3H3* encodes for Prolyl 3-Hydroxylase 3, which plays a key role in collagen biosynthesis, affecting properties of the extracellular matrix [46–49], and has been previously been shown to act as a tumor suppressor in breast, lymphoid, and other cancers [50–52], while *NBL1* encodes for neuroblastoma suppressor of tumorigenicity 1 [53,54], a transcription factor that is involved in the negative regulation of cell cycle (G1/S transition) [55–58]. The overall expression for these genes was low but were consistent with population mean normalized gene expression from the Human Cell Atlas [59]. Analyzing *NBL1* and *P3H3* gene expression in transcripts per million (TPM), in addition to analyzing these as normalized gene counts (standard protocol for bulk RNA-seq analyses which include filtering steps to remove low-expressed genes [60–62]), yielded similar results (*NBL1*: average TPM 3.37, Spearman R = -0.21, p = 0.0029; *P3H3*, average TPM = 2.10, Spearman R = -0.31, p = 1.5x10^-5^). We also performed unbiased gene set enrichment analyses (GSEA) across the transcriptome using rank-ordered genes by q-value and determining normalized enrichment scores (**S1 Table**). HIV total DNA was statistically significantly associated with pathways involving complement activation and humoral immune response (e.g., “regulation of complement activation”, q = 9.8x10^-6^; “humoral immune response mediated by circulating immunoglobulin”, q = 1.6x10^-5^; “B cell mediated immunity”, q = 0.002; “Fc-gamma receptor signaling pathway”, q = 0.020), but these associations were only observed within the European ancestry subgroup.

**Table 2:** Differentially expressed host genes in relation to HIV total DNA. Results shown for the overall cohort of 191 participants using a Benjamini-Hochberg false discovery rate (FDR) cutoff value of q<0.05.

HIV Total DNA							
Gene	Gene Name	p^a^	q^b^	FC^c^	% Change^d^	TPM^e^	Description
**Total Study Population**							
*NBL1*	NBL1, DAN Family BMP Antagonist	6.14E-07	0.012	0.982	-1.8	3.37	NBL1, also known as neuroblastoma suppressor of tumorigenicity 1, is a transcription factor that belongs to the DAN (differential screening-selected gene aberrant in neuroblastoma) family of proteins [53, 54] and is involved in the negative regulation of cell cycle (G1/S transition) [55–58]. In a recent *ex vivo* analysis of CD4+ T cells from rhesus macaques after HIV-1 Env immunization and antibody co-administration, *NBL1* was identified as a host gene that was differentially expressed in all treated (CTLA-4, PD-1, and CTLA-4 + PD-1 Ab) versus control groups [106].
*P3H3*	Prolyl 3-Hydroxylase 3	1.25E-06	0.012	0.984	-1.6	2.10	*P3H3* encodes for Prolyl 3-Hydroxylase 3, which functions as a collagen prolyl hydroxylase (vital for collagen biosynthesis) that affects properties of the extracellular matrix and alters cellular behavior [46–49]. Prior studies suggest that P3H3 plays a role as a tumor suppressor in breast, lymphoid, and other cancers [50–52].

^a^ p = two sided p-value.

^b^ q = two-sided false discovery rate (FDR) Benjamini-Hochberg q-value.

^c^ FC = fold-change in host gene expression per two-fold change in copies of HIV from multivariate model adjusted for age, sex, nadir CD4+ T cell count, timing of ART initiation, ancestry (PCs), and residual variability (probabilistic estimation of expression residuals, PEERs). Bold font denotes genes with q<0.05.

^d^ % Change = percent change in host gene expression per two-fold change in copies of HIV.

^e^ Mean transcripts per million.

**Fig 2:**
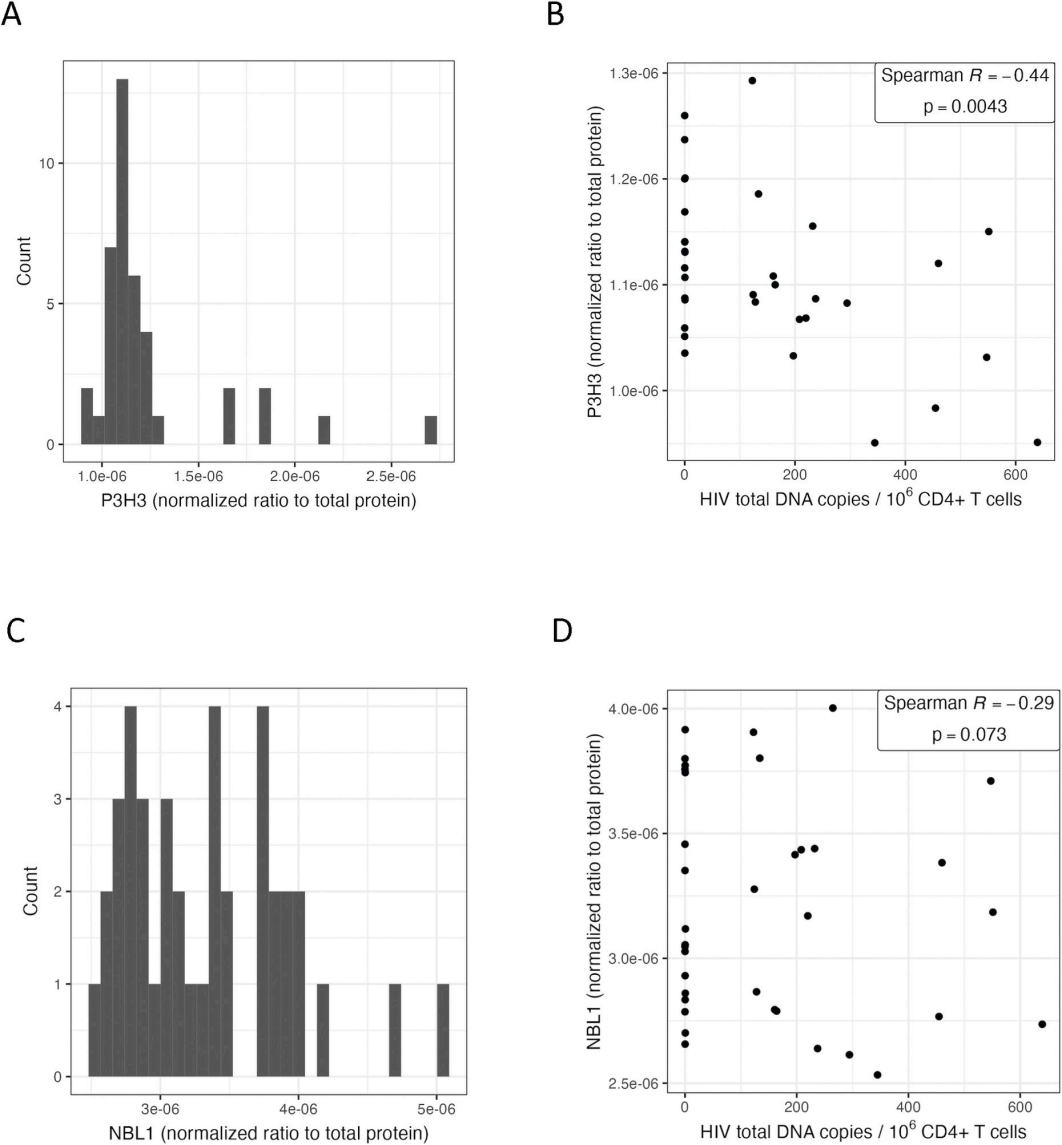
Intracellular P3H3 and NBL1 protein levels from peripheral CD4+ T cells. The distribution of normalized protein levels for P3H3 (A) and NBL1 (C) among a subset of 40 study participants. Correlation scatterplots are shown for P3H3 (B) and NBL1 (D), excluding outliers (>2 standard deviations).

For a subset of 40 participants for whom we had remaining PBMC aliquots, we were able to perform protein validation of *P3H3* and *NBL1* associations with HIV total DNA (**Fig 2**). Both genes encode for intracellular proteins [63,64] and thus, we performed CD4+ T cell isolation followed by ELISA (enzyme-linked immunoassay). P3H3 protein expression levels from peripheral CD4+ T cells demonstrated a significant correlation with HIV total DNA (Spearman R = -0.44, p = 0.0043) (**Fig 2B**), consistent with the RNA-seq observations. Similarly, NBL1 also demonstrated a non-significant inverse trend with HIV total DNA (Spearman R = -0.29, p = 0.073) (**Fig 2D**), consistent with the RNA-seq results. Overall, RNA and protein expression levels were positively correlated, but in this small sample, did not meet statistical significance (P3H3: Spearman R = 0.29, p = 0.067; NBL1: Spearman R = 0.22, p = 0.17) (**S6 Fig**). The inverse associations between protein expression and HIV total DNA were still observed in multivariate models controlling for significant covariates, timing of ART initiation and nadir CD4+ T cell count, but did not meet statistical significance at p<0.05; for each two-fold change in HIV total DNA, there was a -1.2% change in NBL1 protein expression, p = 0.060 (**S2 Table**). Both NBL1 and P3H3 are intracellularly expressed proteins, and protein levels may vary by tissue (e.g., NBL1 is primarily expressed in the central nervous system [63,64]). In our small sample size, we were only able to test whether the RNA-seq findings validated at the protein level from peripheral CD4+ T cells.

### Individuals with higher HIV unspliced RNA had lower expression of host genes involved in innate immunity, inflammasome activation, and inflammation

HIV unspliced RNA was significantly associated with differential expression of several host genes involved in innate immunity, inflammasome activation, and inflammation. In contrast to HIV total DNA, which measures the total reservoir size, HIV RNA roughly estimates the “transcriptionally active” HIV reservoir [65,66], which among the participants with measurable HIV intact DNA (**S3 Fig**), was significantly correlated with usRNA. A total of 17 host genes, the majority of which reflect inflammatory pathways, were significantly lower among individuals with higher HIV usRNA, including after adjustment for significant covariates, timing of ART initiation, nadir CD4+ T cell count, genetic ancestry (PCs), and residual variability (PEERs) (**Table 3**). Specifically, there was approximately a 5–10% decrease in the expression of these host genes for each two-fold increase in HIV usRNA, based on our multivariate model estimates. Host genes associated with HIV usRNA represented inflammasome activation and tumor necrosis factor (*IL1A*: -9.6%, q = 0.012, *CSF3*: -7.5%, q = 0.013; *TNFAIP6*: -7.6%, q = 0.016, *TNFAIP9*: -6.9%, q = 0.031, *TNFAIP5*: -5.9%, q = 0.043), innate immunity (*TLR7*: -7.1%, q = 0.016), and chemokine (*CXCL3*: -7.2%, q = 0.043; *CXCL10*: -9.2%, q = 0.049) signaling genes (**Tables 3 and S3**). The overall expression of these genes in our cohort was low (**Tables 3 and S3**) but overall consistent with average normalized population gene expression reported in the Human Cell Atlas [59].

**Table 3:** Differentially expressed host genes in relation to log_10_copies of HIV unspliced RNA (usRNA). Results shown for the overall cohort of 191 participants (top panel) as well as the European ancestry subgroup (bottom panel) using a Benjamini-Hochberg false discovery rate (FDR) cutoff value of q<0.05. Additional genes meeting an FDR cut-off of q<0.25 are shown in S3 Table.

HIV Unspliced RNA							
Gene	Gene Name	p^a^	q^b^	FC^c^	% Change^d^	TPM^e^	Description
**Full Cohort**							
*KCNJ2*	Potassium Inwardly Rectifying Channel Subfamily J Member 2, kir2.1	1.49E-07	0.003	0.903	-9.7	2.06	*KCNJ2*, encodes for an inwardly rectifying potassium channel (Kir2.1). Inwardly rectifying potassium ion channels can regulate HIV-1 entry and release into host cells [82]. Tight regulation of potassium ion concentrations has been shown to play a critical role in HIV-1 virus production in CD4+ T cells in cell culture models [170]. HIV Nef protein has been shown to increase K+ concentrations in cells [171], and in turn, changes in K+ concentration have been shown to regulate stages in the HIV life cycle (viral entry, replication, and release) [82].
*IL1A*	Interleukin-1 alpha	1.55E-06	0.012	0.904	-9.6	5.81	IL-1α is one of 11 members of the IL-1 family of cytokines [189]. The IL-1 cytokine family that as damage-associated molecular patterns (DAMPs) triggering innate inflammation and also play a key role in angiogenesis, along with tumor necrosis factor and IL-6 [190]. IL-1β is the most widely studied member of the IL-1 family of cytokines and is the primary circulating form of IL-1. IL-1 is an “upstream” pro-inflammatory inducer of interleukin-6 (IL-6) [83], which strongly predicts morbidity (e.g., myocardial infarction, stroke, malignancy) [84–87] and mortality [80,86–89] among people living with HIV on ART. The IL-1 signaling pathway, and in particular, IL-1β, has emerged as a major target for immune modulation [107,159].
*GJB2*	Gap Junction Protein Beta 2	2.26E-06	0.012	0.929	-7.1	0.68	*GJB2*, also known as *CX26*, encodes for gap junction beta 2 protein (connexin 26). Gap junction proteins, or connexins, act as cell-cell communication channels to transport signaling molecules (e.g., K^+^, Ca+, ATP) [83,84], but HIV-1 has been shown to exploit these communication channels to disseminate infection as well as associated inflammation even in the absence of viral replication [85,86]. Connexins are expressed in the endoplasmic reticulum and transported to the plasma membrane as connexin hemichannels that then fuse apposing cells, forming gap junctions [191,192]. A growing body of literature strongly suggests that connexins intensify inflammation by facilitating damage-associated molecular pattern (DAMP) release, which then bind to pattern recognition receptors such as toll-like receptors (TLRs) and nod-like receptors (NLRs). Thus, in several inflammatory diseases, blocking connexin channels has consistently been shown to reduce tissue injury and improve organ function [172].
*KCNJ2-AS1*	KCNJ2 antisense RNA 1	2.49E-06	0.012	0.916	-8.4	5.20	*KCNJ2-AS1* is a long non-coding RNA (lncRNA) encodes for KCNJ2 antisense RNA 1. Although initially considered “transcriptional noise,” non-coding natural antisense transcripts’ role in regulating gene expression has been increasingly recognized, especially in light of recent advances in next generation sequencing and transcriptome assembly [193,194]. Indeed, sense-antisense pairs can act as fast evolving self-regulatory hubs capable of rewiring and regulatory networks [195].
*AC034199*.*1*	*AC034199*.*1*	2.88E-06	0.012	0.946	-5.4	0.28	Novel transcript: no data found in literature in association with HIV or immune response.
*CSF3*	Colony Stimulating Factor 3 (G-CSF)	3.93E-06	0.013	0.925	-7.5	8.04	*CSF3* encodes for granulocyte stimulating factor 3 (G-CSF), a member of the IL-6 superfamily of cytokines [115]. G-CSF is mostly known for its role as a growth factor for neutrophils, promoting the proliferation and survival of neutrophil precursors. However, G-CSF has also been shown to regulate T cell responses via induction of IL-10 secretion [167], leading to inhibition of CD4+ and CD8+ T cell responses and reduction of cytotoxic responses [168]. Indeed, donor treatment of pegylated G-CSF was found to increase IL-10-producing regulatory T cells (Tregs) and enhance transplant tolerance [169], suggesting that G-CSF can directly modify T cell responses via IL-10 [196].
*TNFAIP6*	Tumor Necrosis Factor alpha induced protein 6	5.99E-06	0.016	0.924	-7.6	1.14	*TNFAIP6* encodes for pattern recognition receptor, Tumor Necrosis Factor alpha induced protein 6 which functions as an anti-inflammatory protein [197,198], is induced by IL-1 (upon LPS-stimulation) [118,119], and interacts with *TNFAIP5* [199,200].
*TLR7*	Toll Like Receptor 7	6.59E-06	0.016	0.929	-7.1	0.60	*TLR7* encodes for a PRR that senses HIV single-stranded RNA) [160,161] and has been associated viral persistence in several human and non-human primate studies [128–130]. TLR7 agonist administration has been associated with delayed viral rebound [128] and reduced viral reservoirs in non-human primate studies [129]. A human clinical trial of the TLR7 agonist GS-9620 recently demonstrated a delay in viral rebound in HIV controllers after cessation of ART (NCT05281510) [130]. *TLR7* is located on the X chromosome, and host *TLR7* transcriptional activity has been linked to acute viremia in HIV+ women (linked to type I interferon production) [132] as well as with enhanced innate immune function (i.e., plasmacytoid dendritic cell IFN-α and TNF-α production) [131].
*MRAS*	Muscle RAS oncogene homolog	1.07E-05	0.024	0.945	-5.5	0.17	*MRAS* encodes for a protein in the Ras family of small GTPases which functions as signal transducers in cellular processes.
*TNFAIP9/* *STEAP4*	Tumor Necrosis Factor alpha induced protein 9	1.53E-05	0.031	0.931	-6.9	0.83	*TNFAIP9* encodes for pattern recognition receptor, Tumor Necrosis Factor alpha induced protein 9, also known as *STEAP4* (six transmembrane epithelial antigen of prostate 4). TNFAIP9 has been shown to negatively regulate NF-κB, STAT-3 signaling, and IL-6 production [150,151].
*MIR3945HG*	MIR3945 Host Gene	2.09E-05	0.038	0.942	-5.8	0.36	*MIR3945HG* is an interferon stimulated lncRNA.
*DAPK1-IT1*	DAPK1 Intronic Transcript 1	2.72E-05	0.043	0.950	-5.0	1.11	*DAPK1-IT1* is a lncRNA transcribed from the death associated protein kinases 1 (DAPK1).
*OR2B11*	Olfactory Receptor Family 2 Subfamily B Member 11	2.96E-05	0.043	0.939	-6.1	1.04	OR2B11 is a member of G-protein-coupled receptors (GPCR) responsible for the recognition and G protein-mediated transduction of odorant signals.
*CXCL3*	C-X-C Motif Chemokine Ligand 3	3.03E-05	0.043	0.928	-7.2	31.06	*CXCL3* encodes for C-X-C Motif Chemokine Ligand 3, a member of CXC subfamily called cytokine-induced neutrophil chemoattractant (CINCs). CXCL3 regulates monocyte migration [201,202], neutrophils chemoattraction [202–204], and angiogenesis [205], and is induced by proinflammatory IL-17 [206,207].
*TNFAIP5 /PTX3 /TSFG14*	TNF Alpha-Induced Protein 5 (TNFAIP5), Pentraxin-related protein (PTX3), Tumor Necrosis Factor-Inducible Protein TSG-14 (TSG14).	3.27E-05	0.043	0.941	-5.9	15.37	*TNFAIP5* encodes for a pattern recognition receptor, TNF-α induced protein 5, also known as pentraxin-related protein (PTX3) or tumor Necrosis Factor-Inducible Gene 14 Protein (TSFG14). TNFAIP5, or PTX3, is induced in response to TNF-α, TLR engagement and IL-1β signaling and is part of the pentraxin superfamily of proteins, which includes C-reactive protein (CRP) and serum amyloid [208]. TNFAIP5 is a soluble PRR that plays a key role in host antimicrobial innate immune defense but also functions in complement activation and regulating inflammation in response to tissue repair and cancer [209]. Elevated serum levels of PTX have been associated with severity and survival in patients with sepsis [210].
*RRN3P4*	RRN3 Pseudogene 4	3.97E-05	0.049	0.950	-5.0	0.83	Pseudogene
*CXCL10*	C-X-C Motif Chemokine Ligand 10	4.21E-05	0.049	0.908	-9.2	5.79	*CXCL10* encodes for IP-10 (interferon gamma-induced protein 10) which recruits activated Th1 lymphocytes to sites of infection [120–122] and in HIV, signals through TLR7/9-dependent pathways [122], predicts HIV disease progression [123,124], correlates with acute HIV seroconversion [125], and promotes HIV latency [126,127].
**European Ancestry Subpopulation**							
*TLR7* ^*f*^	Toll Like Receptor 7	1.48E-06	0.018	0.906	-9.4	0.60	*TLR7* encodes for a PRR that senses HIV single-stranded RNA) [160,161] and has been associated viral persistence in several human and non-human primate studies [128–130].
*GJB2* ^*f*^	Gap Junction Protein Beta 2	2.70E-06	0.018	0.909	-9.1	0.68	*GJB2*, also known as *CX26*, encodes for gap junction beta 2 protein (connexin 26). Gap junction proteins, or connexins, act as cell-cell communication channels to transport signaling molecules (e.g., K^+^, Ca+, ATP) [83,84], but HIV-1 has been shown to exploit these communication channels to disseminate infection as well as associated inflammation even in the absence of viral replication [85, 86]. Connexins are expressed in the endoplasmic reticulum and transported to the plasma membrane as connexin hemichannels that then fuse apposing cells, forming gap junctions [191,192]. A growing body of literature strongly suggests that connexins intensify inflammation by facilitating damage-associated molecular pattern (DAMP) release, which then bind to pattern recognition receptors such as toll-like receptors (TLRs) and nod-like receptors (NLRs). Thus, in several inflammatory diseases, blocking connexin channels has consistently been shown to reduce tissue injury and improve organ function [172].
*AC034199*.*1*^*f*^	Ac034199.1	3.09E-06	0.018	0.930	-7.0	0.28	novel transcript
*PPP1R17*	Protein Phosphatase 1 Regulatory Subunit 17	4.20E-06	0.018	0.934	-6.6	0.09	PPP1R17 (Protein Phosphatase 1 Regulatory Subunit 17) is a substrate for cGMP-dependent protein kinase.
*IGSF6*	Immunoglobulin Superfamily Member 6	4.73E-06	0.018	0.972	-2.8	2.91	Immunoglobulin superfamily member 6 (IGSF6) is also known as downregulated by activation (DORA).
*AL133163*.*2*	Al133163.2	7.81E-06	0.025	0.945	-5.5	0.33	novel transcript

^a^ p = two sided p-value.

^b^ q = two-sided false discovery rate (FDR) Benjamini-Hochberg q-value.

^c^ FC = fold-change in host gene expression per two-fold change in copies of HIV from multivariate model adjusted for age, sex, nadir CD4+ T cell count, timing of ART initiation, ancestry (PCs), and residual variability (probabilistic estimation of expression residuals, PEERs).

^d^ % Change = percent change in host gene expression per two-fold change in copies of HIV.

^e^ Mean transcripts per million.

^f^ % also significant in full cohort analysis.

### Pathway-based analyses further identified gene sets involving NRLP3 inflammasome, TLR4/microbial sensing, TNF-α, and IL-10 signaling that were associated with HIV usRNA

**Fig 3:**
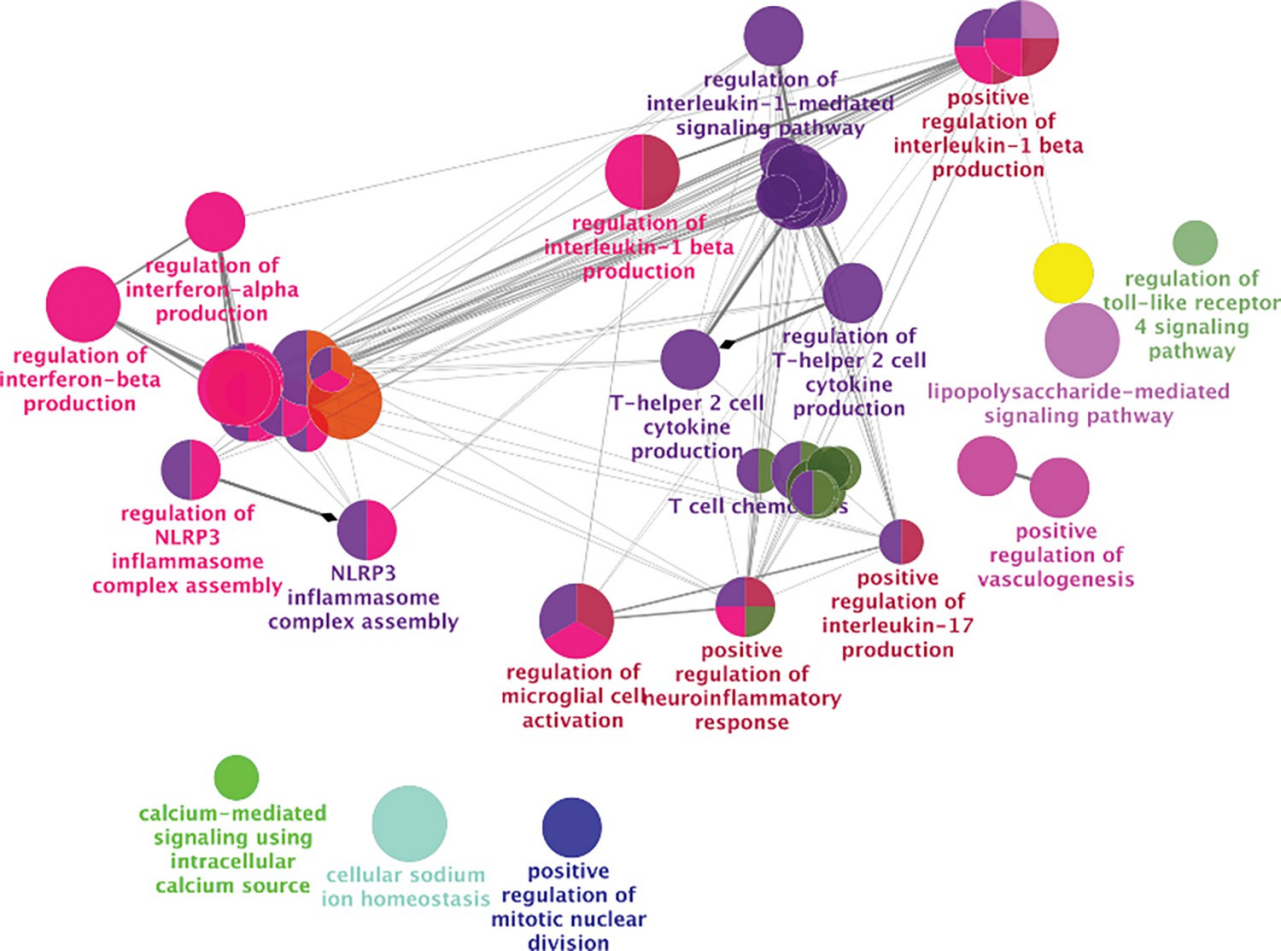
Clustering of top differentially expressed host genes associated with HIV unspliced RNA. Given the large number of statistically significant host genes (Tables 3 and S3 Table), we used network analysis to group the top-ranked genes (q<0.25) into biologically interpretable clusters (ClueGo Network software). Host genes related to NRLP3 inflammasome activation (e.g., IL-1β), Th2 cell cytokine production (e.g., IL-10), and bacterial translocation (e.g., TLR4, lipopolysaccharide) signaling were significantly associated with HIV usRNA. A Benjamini-Hochberg false discovery rate (FDR) of q<0.05 was used to generate nodes (circles) based on kappa scores ≥0.4. The size of the nodes reflects the enrichment significance of the terms, and the different colors represent distinct functional groups. Created with https://apps.cytoscape.org/apps/cluego.

**Fig 4:**
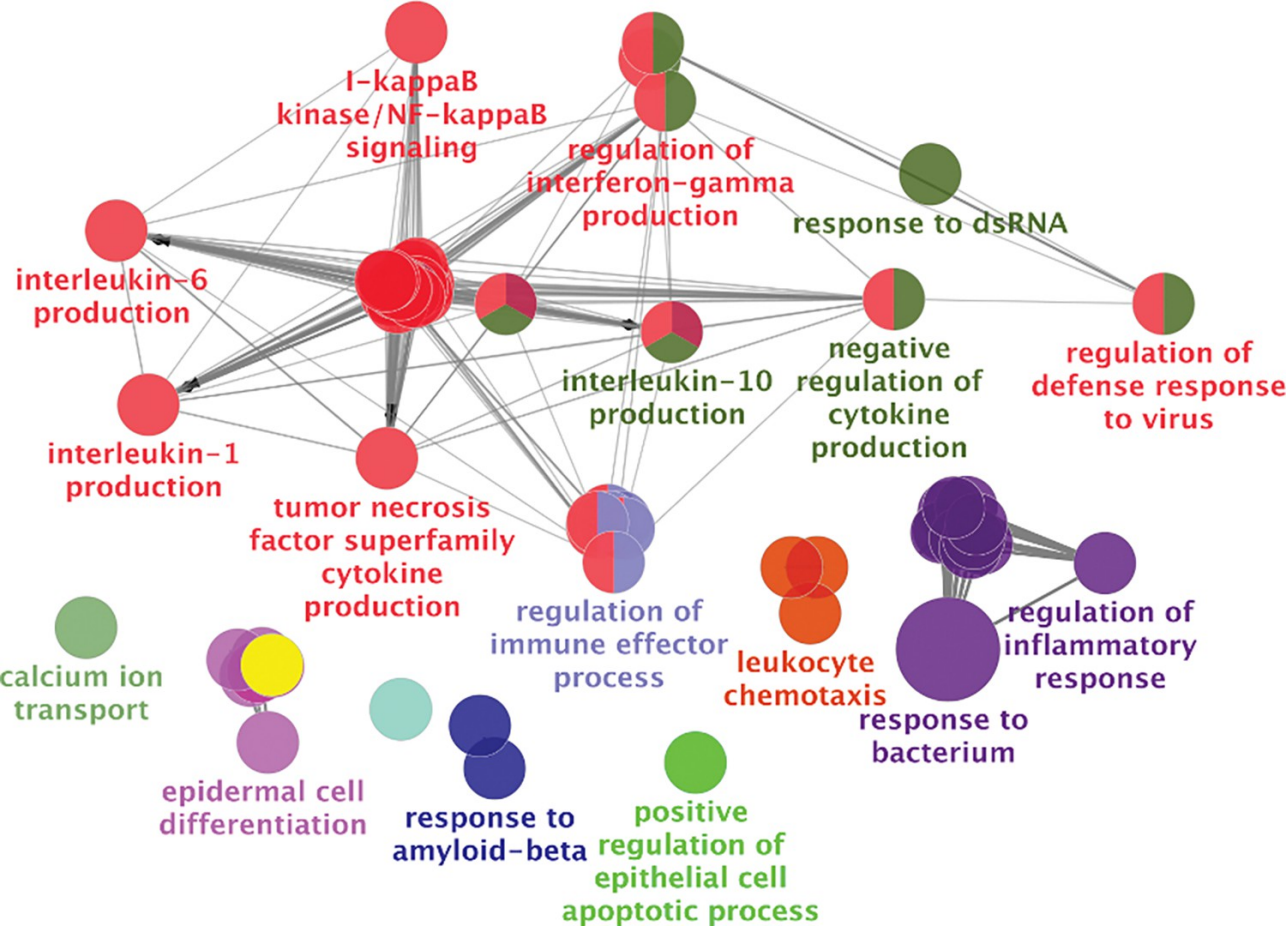
Unbiased gene set enrichment analyses (GSEA) of the entire transcriptome (rank-ordered by q-value) in association with HIV unspliced RNA. Host gene sets involving interferon, IL-10, TNF, NLRP3 inflammasome activation (e.g., IL-1β, IL-6), and bacterial translocation (e.g., lipopolysaccharide-mediated) signaling were significantly associated with HIV usRNA. A Benjamini-Hochberg false discovery rate (FDR) of q<0.05 was used to generate nodes (circles) based on kappa scores ≥0.4. The size of the nodes reflects the enrichment significance of the terms, and the different colors represent distinct functional groups. Created with https://apps.cytoscape.org/apps/cluego.

Given the large number of genes associated with HIV usRNA, we also performed network analyses to interpret clusters of pathways from the gene expression data. We applied the ClueGo network analysis application to visualize the ranked genes (q<0.25) into biologically interpretable clusters [67]. Several key pathways involving inflammasome activation [68–71] and bacterial translocation [72–74] genes were strongly associated with HIV usRNA. These include gene sets involving NLRP3 (NOD-, LRR- and pyrin domain-containing protein 3) inflammasome/IL-1β signaling, as well as pathways involving microbial translocation, such as toll-like receptor 4, lipopolysaccharide (LPS), and IL-17 signaling (**Fig 3**). We also used unbiased gene set enrichment analyses (GSEA) using rank-ordered genes in the entire transcriptome to further identify biologically relevant clusters of genes associated with HIV usRNA. Pathways reflecting microbial translocation (“Response to Bacterium”, q = 7.5x10^-5^; “Cellular Response to Lipopolysaccharide”, q = 0.006), IL-1 signaling (“Interleukin-1 beta production”, q = 0.008; “Regulation of Interleukin-1 Production”, q = 0.008), and cytokine production (“Tumor Necrosis Factor Production”, q = 0.006; “Tumor Necrosis Factor Superfamily Cytokine Production”, q = 0.006; “Regulation of Tumor Necrosis Factor Production”, q = 0.008) were again associated with HIV usRNA. In addition, several gene sets related to IL-10 signaling (“regulation of interleukin-10 production”, q = 0.037, “Interleukin-10 production”, q = 0.041), a pleiotropic cytokine associated HIV immune dysregulation and persistence [75–77], were also significantly associated with HIV usRNA (q = 0.04) (**Fig 4, S4 Table**).

**Fig 5:**
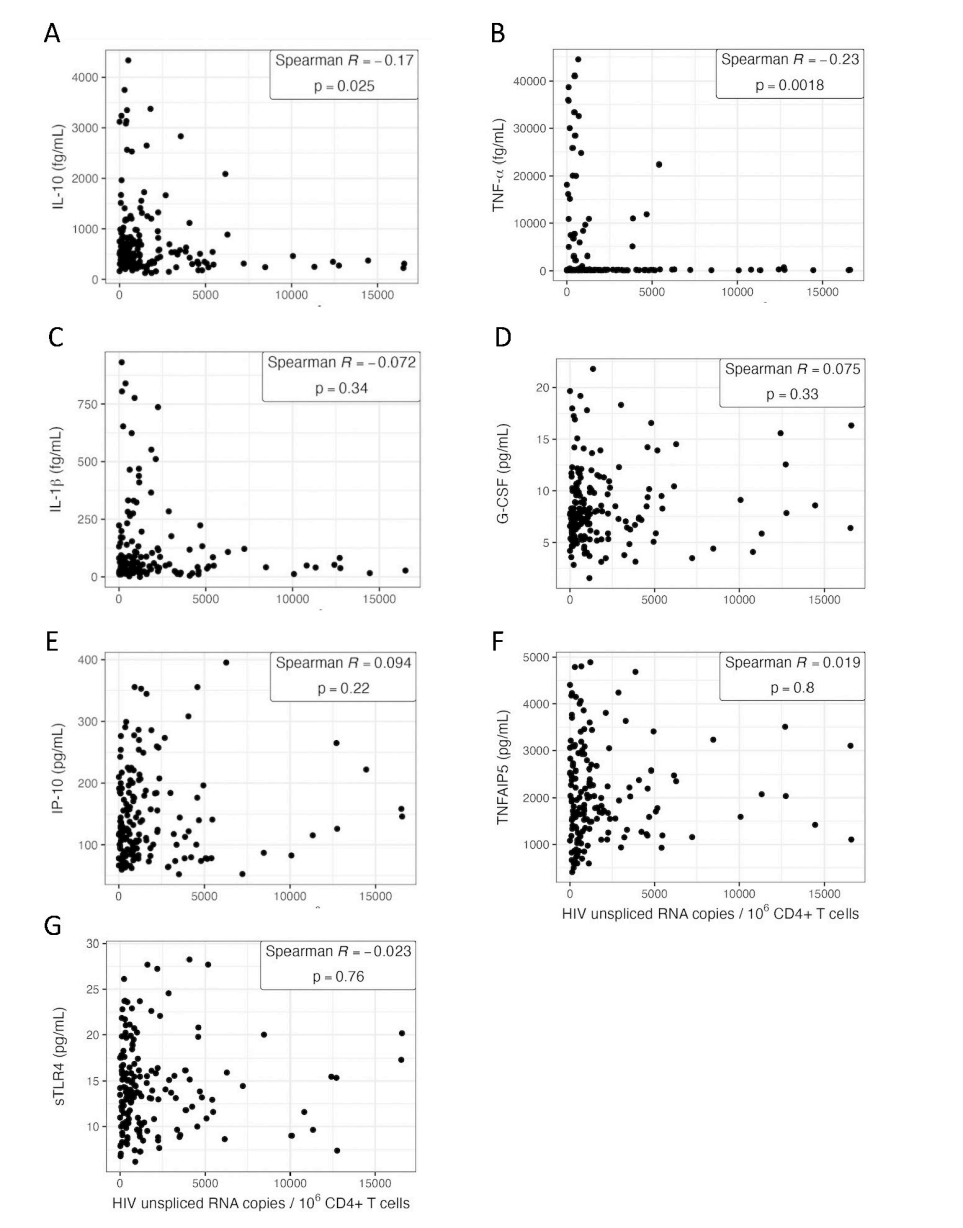
Plasma cytokine expression of IL-10 (A), TNF-α (B), IL-1β (C), G-CSF (D), IP-10 (E), TNFAIP5 (F), and sTLR4 (G). The association between plasma protein expression among 175 study participants in relation to measures of HIV unspliced RNA.

Since several of the host genes and gene sets associated with HIV usRNA reflected cytokine signaling (**Tables 3 and S3, Figs 3 and 4**), we obtained matched cryopreserved plasma samples from 175 of the 191 participants and designed a series of high-sensitivity multiplex cytokine protein assays (Meso Scale Diagnostics and LSBio). We were able to design assays for eight proteins: IL-1α, IL-1β, IL-10, TNF-α, G-CSF, IP-10, TNFAIP5, and sTLR4. Unfortunately, we were unable to perform protein validation of IL-1α since plasma levels were undetectable in most of our samples, potentially due to this cytokine’s primarily intracellular expression, mostly from monocytes/macrophages [78–81]. Overall, for the 7 genes, the RNA and protein expression levels were positively correlated, and were statistically significant for IL-10, TNF-α, and IL-1β (IL-10: Spearman R = 0.34, p = 4.3x10^-6^; TNF-α: Spearman R = 0.19, p = 0.011; IL-1β: Spearman R = 0.29, p = 1.9x10^-4^) (**S7 Fig**). Of the final set of 7 plasma cytokines assayed, two cytokines, IL-10 and TNF-α, were significantly correlated with HIV usRNA (IL-10: Spearman R = -0.17, p = 0.025; TNF-α: Spearman R = -0.23, p = 0.0018) (**Fig 5**), although these associations did not meet statistical significance in multivariate linear models adjusted for significant covariates (**S5 Table**). Of note, plasma IL-10 was also significantly inversely correlated with HIV total DNA (Spearman R -0.18, p = 0.016) (**S8 Fig**), even though the *IL10* gene did not meet FDR-adjusted statistical significance in the RNA-seq analysis with HIV DNA per se (**S2 Table**).

### Individuals with higher HIV unspliced RNA had lower expression of host genes encoding membrane channels involved in HIV-1 entry (*KCNJ2*) and cell-cell communication (*GJB2*)

**Fig 6:**
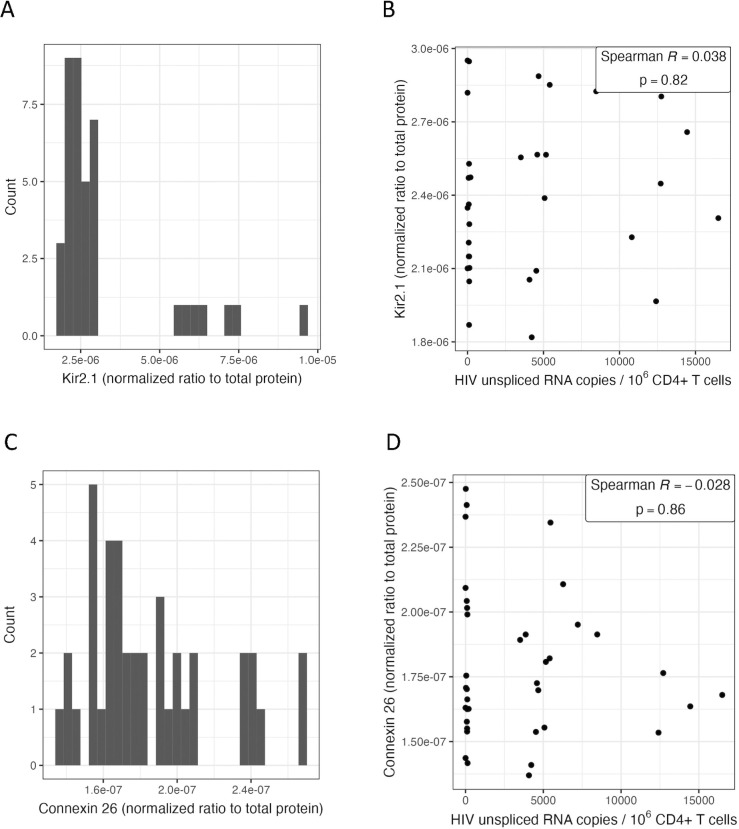
Intracellular Kir2.1 (*KCNJ2*) and connexin 26 (*GJB2*) protein levels from peripheral CD4+ T cells. The distribution of normalized protein levels for Kir2.1 (A) and connexin 26 (C) among a subset of 40 study participants. Correlation scatterplots are shown for Kir2.1 (B) and connexin 26 (D), excluding outliers (>2 standard deviations).

HIV usRNA was also inversely associated with two genes, *KCNJ2* (-9.7%, q = 0.003) and *GJB2* (-7.1%, q = 0.012), encoding for membrane channel proteins, Kir2.1 and connexin 26, respectively (**Table 3**). *KCNJ2* encodes for an inwardly rectifying potassium channel, a class of channels that have been shown to regulate HIV-1 entry and release [82], and *GJB2*, or *CX26*, encodes for gap junction beta 2 protein (also known as connexin 26). Gap junctions act as critical cell-cell communication channels for transport signaling molecules and performing physiologic functions, but are often closed or downregulated under pathologic conditions [83,84]. HIV-1 has been shown to exploit these communication channels to disseminate infection as well as associated inflammation even in the absence of viral replication [85,86]. Of note, *KCNJ2-AS1*, the antisense long non-coding RNA transcript for *KCNJ2*, was also statistically significantly associated with HIV usRNA (-8.4%, q = 0.012) (**Table 3**). Since both *KCNJ2* and *GJB2* encode for membrane-associated proteins [63,64], we performed protein validation from CD4+ T cell isolates for whom we had remaining PBMCs (as we had done above for intracellular protein validation of P3H3 and NBL1). For both genes, RNA and protein expression levels were positively correlated, and the correlation was statistically significant for *GJB2*/connexin 26 (Spearman R = 0.37, p = 0.02) (**S9 Fig**), but we did not observe significant correlations with these proteins as observed in our RNA-seq analyses when testing peripheral CD4+ T cells from a small subset of 40 participants in our cohort (**Fig 6, S6 Table**).

### HIV intact DNA was not significantly associated with host gene expression, but assessment of this may have been limited by a large proportion of undetectable values

HIV intact DNA was undetectable in nearly half (48%) of our measured samples (**S3B and S3C Fig**), which may have decreased statistical power to detect differentially expressed genes in relation to HIV intact DNA. As previously described, we employed a conservative approach of assigning a zero value for HIV intact DNA if one of two HIV-1 target assay results were undetectable [31,87], since even one target with defective proviral sequence suggests non-intact HIV DNA. Nonetheless, the frequency of undetectable values in our cohort is considerably higher than prior reports using similar (2 or more target) HIV intact DNA assays [88–96]. In contrast, HIV total DNA results using a different assay (qPCR) was measurable in 95% of samples from our cohort (**S3A and S3C Fig**), suggesting a potential difference due to assay method and/or input sample DNA concentration (HIV intact DNA was performed using remaining extracted DNA after first performing HIV total DNA by qPCR, and there was a general trend demonstrating measurable HIV intact DNA copies with higher concentrations of input sample DNA, **S10 Fig**). Nonetheless, among participants with measurable HIV intact DNA, differential gene expression analyses demonstrated a slight positive trend (q<0.25) with *PLGLB1* (+6.0%, q = 0.23), which encodes for a protein that inhibits thrombus degradation, and *AGL* (+0.9%, q = 0.23), which encodes for an enzyme involved in glycogen degradation (**S7 Table**). Gene set enrichment analyses demonstrated trends with pathways involving neutrophil activation (“Neutrophil Degranulation”, q = 0.046; “Neutrophil Activation Involved in Immune Response”, q = 0.046; “Leukocyte Activation”; q = 0.046), and among the European subgroup, pathways associated with myeloid-mediated immunity (“Myeloid Leukocyte Mediated Immunity”; q = 0.058; “Myeloid Cell Activation Involved in Immune Response”; q = 0.060) (**S8 Table**).

### Proposed Model of Host Gene Expression and HIV Reservoir from Peripheral CD4+ T Cells

**Fig 7:**
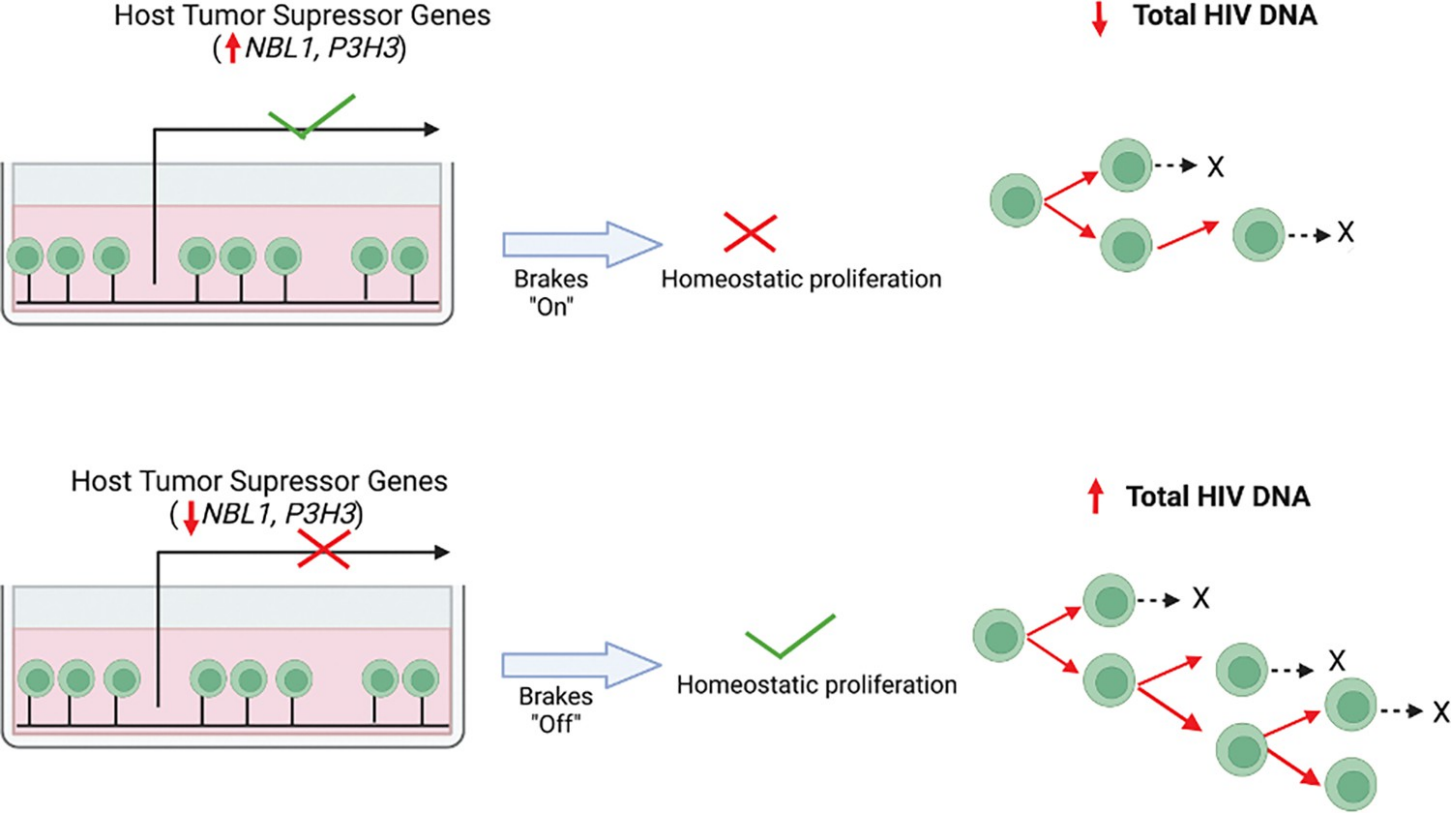
Proposed model based on host bulk RNA-sequencing and HIV reservoir quantification from peripheral CD4+ T cells of 191 ART-suppressed people with HIV. Increased expression of host tumor suppressor genes *NBL1* and *P3H3* were observed among individuals with smaller HIV total DNA reservoir size in our cross-sectional study (q<0.05). The model depicts host genes from HIV-uninfected cells influencing the subset of CD4+ T cells harboring provirus. Created with BioRender.com.

**Fig 8:**
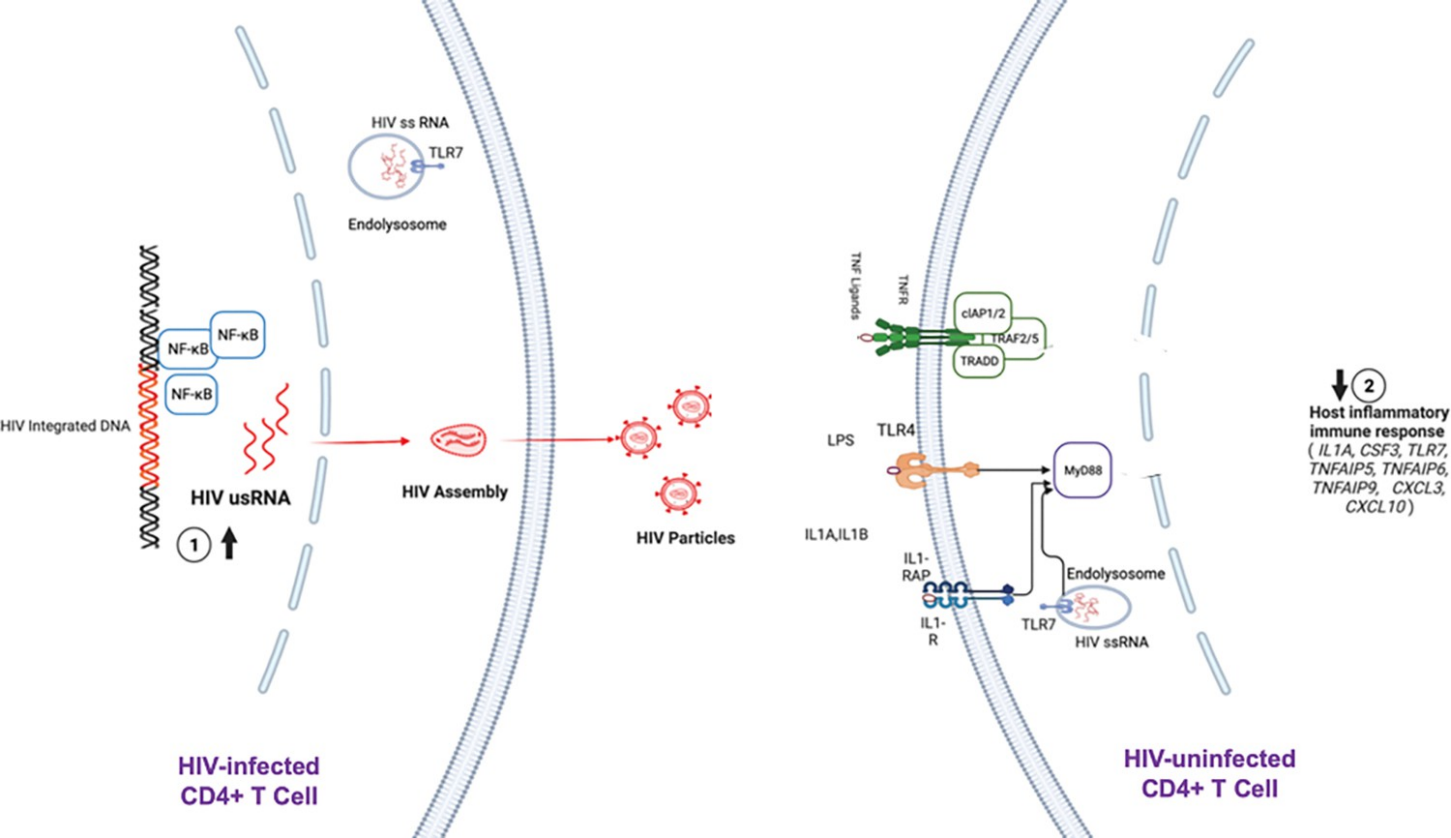
Proposed model for the inverse association between HIV unspliced RNA and host gene expression in our cross-sectional study of 191 ART-suppressed people living with HIV. Within bulk peripheral CD4+ T cells, higher transcriptional reservoir activity (HIV usRNA) from HIV+ cells (left) may chronically lead to downregulation of host proinflammatory gene expression in bystander cells (right) in an attempt to suppress persistent inflammation and innate immune activation. Created with BioRender.com.

While our cross-sectional study from bulk peripheral CD4+ T cells was unable to demonstrate direct causality, based on the known biologic function(s) of these host genes, we propose a hypothetical feedback loop whereby host genes expressed from uninfected CD4+ T cells act as drivers of the total HIV-infected CD4+ T cell reservoir (**Fig 7**), and in return, host innate/inflammatory genes from uninfected CD4+ T cells respond to the transcriptionally active HIV-infected CD4+ T cell reservoir (**Figs 8 and S11**).

## Discussion

To our knowledge, this is the largest cohort-based transcriptomic study of host genetic predictors of the HIV reservoir. We observed only two host genes (*P3H3*, *NBL1*) that were significantly (inversely) associated with HIV total DNA, both of which are known tumor suppressor genes and regulate cell cycle. We also observed 17 host genes that were significantly associated with HIV usRNA, all of which demonstrated an inverse relationship with HIV usRNA and are highly interrelated pathways involved in inflammation (e.g., IL-1, IL-6, IL-10, TNF-α, TLR4, NRLP3 inflammasome signaling). We did not observe any host genes that were significantly associated with HIV intact DNA, but this may have been due to a large number of undetectable provirus in our population, potentially due to low sample input DNA concentrations. Protein validation from a subset of participants with remaining biospecimens supported a significant correlation between HIV total DNA and P3H3 expression (from CD4+ T cells), and between HIV usRNA with plasma IL-10 and TNF-α levels.

Prior HIV integration studies have identified several host oncogenes and/or cell cycle genes that enriched for HIV integrations during long-term ART [97–103]. Tumor suppressor genes encoding for p53 (*TP53*) and p21 (*CDKN1A*) have previously been associated with inhibition of HIV early replication [104] and blockade of HIV infection [105]. We observed statistically significant associations for HIV total DNA and two tumor suppressor genes, *NBL1* and *P3H3*, using a stringent false discovery rate q<0.05. In a recent *ex vivo* analysis of CD4+ T cells from rhesus macaques after HIV-1 Env immunization and antibody co-administration, *NBL1* was identified as a host gene that was differentially expressed in all treated (CTLA-4, PD-1, and CTLA-4 + PD-1 Ab) versus control groups [106]. Our protein validation of these two host genes associated with HIV total DNA from peripheral CD4+ T cells demonstrated similar inverse trends as observed in our RNA-seq analyses. It is unclear whether these particular genes (*P3H3* and *NBL1*) versus “tumor suppressor” genes in general, which broadly function as regulators of cell cycle, may have important roles in HIV persistence.

A much larger set of host genes were strongly associated with HIV usRNA. Since we analyzed over 20,000 genes in the human transcriptome, stringent false discovery rate (FDR)-correction methods were employed. These included data dimensionality reduction approaches (principal component analyses [36]) using whole exome data [37] and inclusion of PEER factors (probabilistic estimator of expression residuals) in multivariate models to further account for residual variability in the data [45]. Even after these additional measures, we observed 17 statistically significant genes associated with HIV usRNA that met stringent FDR q<0.05 in several interrelated immune signaling pathways, many of which have been previously shown to play an important role in the host response to HIV [75–77,107–138].

We were able to perform protein validation of several of these genes reflecting secreted cytokines using matched plasma samples from 175 of the 191 study participants (encoding for IL-1α, IL-1β, IL-10, TNF-α, G-CSF, IP-10, TNFAIP5, and sTLR4; IL-1α was undetectable in most of our samples despite high-sensitivity assay, potentially due to its intracellular expression [78–81]). Plasma IL-10 and TNF-α were significantly associated with HIV usRNA (TNF-α, even after adjustment for nadir CD4+ T cell count and timing of ART initiation). Interestingly, plasma IL-10 was also inversely associated with HIV total DNA. IL-10 is a complex pleiotropic cytokine that has been highly studied in several autoimmune and infectious diseases and possess complex actions that vary by stage of infection and by tissue [139]. IL-10 can both inhibit pathogen clearance and reduce excessive immunopathology, thus exhibiting both an inflammatory and regulatory response [140,141]. For example, IL-10 plays a critical role in intestinal homeostasis and both induces and prevents mucosal damage [142]. Thus, in several infectious diseases (viral, bacterial, fungal, parasitic), the effect of IL-10 varies by stage of infection and by tissue [139–141,143–148]. Here, as observed with several of the other differentially expressed host genes in relation to HIV usRNA, plasma IL-10 was inversely associated with HIV usRNA. These findings are in contrast to a recent non-human primate study by Harper et. al., where rhesus macaques ART-suppressed for 7 months demonstrated that higher plasma IL-10 levels were associated with larger SIV DNA reservoirs, suggesting that IL-10 maintains long-lived reservoir cells [75]. (The authors did not evaluate or report findings for SIV RNA but did show that *in vivo* neutralization of soluble IL-10 with a monoclonal antibody resulted in a 2-log increase in plasma IL-10). Differences in timing of ART initiation, duration of ART, and/or cross species differences might explain our contrasting findings. An alternate explanation might be that during long-term ART, IL-10 contributes to an ongoing dynamic interplay between the host immune response and low levels of HIV transcription, similar to complex host-viral dynamics that have been described associated with type I interferons during acute versus chronic infection [149].

Plasma TNF-α was the other cytokine significantly associated with HIV usRNA, consistent with the RNA-seq associations with TNF pathway genes (*TNFAIP5*, *TNFAIP6*, *TNFAIP9)*. These TNF-associated pattern recognition receptors (PRRs) not only respond to TNF-α, but also respond to IL-1 signaling and toll-like receptor (TLR) engagement [116–119], and negatively regulate NF-κB signaling and IL-6 production [150–155]. IL-10 has been shown to block HIV-induced TNF-α and IL-6 secretion and inhibit HIV replication [156], and a cohort study of 51 people with HIV suppressed on ART demonstrated decreasing plasma IL-10/TNF-α ratio to be associated with AIDS progression [157].

The remaining host genes associated with HIV usRNA did not replicate in our limited set of protein validation assays, but collectively represent highly interrelated immune pathways that warrant further study. Plasma IL-6 significantly predicts mortality in ART-suppressed people with HIV in several large cohort studies [110–114]), and IL-1β, an upstream inducer of IL-6, has emerged as a major target for HIV immune modulation [107,158,159]. IL-1α can act as a “dual function” cytokine, directly sensing intracellular DNA damage as well as a proinflammatory mediator, but it is mostly found intracellularly [78,79]. We observed two toll-like receptors (TLRs) from our analyses, *TLR7* (in the differential gene expression analysis) and *TLR4* (in the pathway-based analysis), to be associated with HIV usRNA. *TLR7* encodes for a PRR that senses HIV single-stranded RNA [160,161] and has been associated with viral persistence in several human and non-human primate studies [128–130]. Host *TLR7* transcriptional activity has been linked to acute viremia in women with HIV [132] as well as with enhanced innate immune function (i.e., IFN-α and TNF-α production) [131], but TLR7 is only expressed intracellularly [162], so we were unable to include it in our protein validation experiments. *TLR4* encodes for a PRR mediating the inflammatory response to microbial translocation [163] (e.g., bacterial endotoxin products such as lipopolysaccharide [164], which are significantly higher in people with HIV compared to uninfected individuals and are associated with AIDS progression [133–138]). *CXCL3* and *CXCL10* encode for chemokines that recruit immune cells to sites of inflammation [165,166] and signal through innate immune pathways (e.g., TLRs) [122]. IP-10 (encoded for by *CXCL10*) has been strongly linked to HIV disease progression and persistence [125–127]. *CSF3* encodes for G-CSF (granulocyte stimulating factor), which is also part of the IL-6 superfamily of cytokines [115]. G-CSF (granulocyte stimulating factor), encoded by *CSF3*, has been shown to regulate T cell responses via induction of IL-10 [167], inhibiting CD4+ and CD8+ T cell responses [168], and has been shown to increase IL-10-producing regulatory T cells (Tregs) [169].

We also identified novel associations between HIV usRNA and two genes (*KCNJ2*, *GJB2*) encoding for membrane channel proteins previously linked to HIV-1 entry and release, and cell-cell communication, respectively. Tight regulation of potassium ion concentrations have been shown to play a critical role in HIV-1 virus production in CD4+ T cells in cell culture models [170], and the HIV Nef protein has been shown to increase K+ concentrations in cells [171] while changes in K+ concentration have been shown to regulate the HIV life cycle (e.g., viral entry, replication, and release) [82]. HIV-1 has been shown to exploit gap junction protein channels to disseminate infection as well as associated inflammation even in the absence of viral replication [85, 86], and a growing body of literature strongly suggests that connexins intensify inflammation by facilitating damage-associated molecular pattern release and binding to PRRs such as TLRs [172]. From our small subset of 40 participants, however, protein expression from CD4+ T cells did not validate the RNA-seq findings.

The study has several limitations that deserve mention. First, although the HIV reservoir has been shown to be relatively stable over time [17,91,173], our cross-sectional design limits the ability to demonstrate causality and simply provides a “snapshot” of the HIV reservoir after a median of 5.1 years of ART suppression. However, based on the known functions of the significantly associated host gene, we have proposed at least two potential models that need to be further validated in longitudinal and functional genomics studies. Indeed, the true *in vivo* associations might involve more complex feedback pathways between the HIV reservoir and host responses. Second, as is characteristic of our San Francisco-based HIV+ population, our study included mostly males of European ancestry. We accounted for this using well-established GWAS-based methods to adjust for population stratification bias [36,174], as well as the use of PEERs [45], which account for residual variance that is characteristic of RNA-seq data. Third, we quantified the peripheral CD4+ T cell reservoir, but the majority of the HIV reservoir persists in lymphoid tissues, such as in the gut-associated lymphoid tissues [175,176]. Recent data suggests that the tissue compartment largely reflects (and is the likely source of) the peripheral compartment [177–179]. While several of the genes associated with HIV usRNA reflect tissue-based inflammation (e.g., IL-10), future studies will need to determine whether our findings from the blood reservoir are relevant to the tissue HIV reservoir. We also performed bulk RNA-seq from peripheral CD4+ T cells and did not perform separate analyses by HIV-infected versus uninfected cells (given the limited availability of PBMCs for our study participants). Thus, the interpretation of our findings likely largely represents host gene expression differences among HIV-uninfected cells, given the infrequency of CD4+ T cells harboring provirus in an aliquot of 10 million cells. Finally, while we selected participants to focus on HIV “non-controllers,” our findings may also be applicable to HIV elite and/or post-treatment controllers. The purpose of this transcriptomic analysis as well as our previously published whole exome sequencing analysis [37] from this cohort were to focus on previously undescribed host genetic predictors of the HIV reservoir (signals that might be lost amidst a study population enriched for previously reported strong genetic effects, such as with HLA and/or *CCR5Δ32* [21–24]) that may be applicable to the large majority of people with HIV who are unable to suppress virus in the absence of therapy.

We did not observe statistically significant associations with HIV intact DNA and host genes. We believe that this may be because HIV intact DNA was undetectable in 48% of our measured samples (while for example, total DNA was measurable in 95% of samples). Here, we performed the HIV intact DNA assay after already allocating DNA for whole exome sequencing and for HIV total DNA quantification by qPCR [37]. Therefore, the low intact DNA detection rate may have been due to (1) low sample input DNA, (2) low true frequencies of cells containing intact proviruses, and/or (3) misclassification of “intact” provirus (primer/probe mismatches described for these type of assays [96]). We quantified HIV intact DNA by targeting five regions of the HIV genome, including regions that are highly conserved when present but are also often deleted, as well as an Env region with frequent hypermutations, across two droplet digital PCR reactions [31]. This allowed the analysis of potentially replication-competent (“intact”) proviral genomes by quantifying the number of droplets positive for 3 targets per each of the two reactions, and then mathematically combining the results of both reactions to calculate the number of HIV genomes containing all 5 regions (5T-IPDA). Zero intact proviral copies was assigned when either of the two reactions failed to detect all three regions in any of the droplets. While we are unable to determine potential primer-mismatches as an underlying cause (since we did not perform full-length or near full-length sequencing), this may have also influenced our results since we have previously shown that the frequency of primer mismatches is likely higher in assays interrogating a larger number of HIV-1 targets (e.g., our 5-target assay compared to a previously published 2-target assay [42]).

Overall, findings from our cross-sectional cohort identified several biologically plausible genes and immune pathways that may be associated with the HIV reservoir. In particular, we observed two host genes associated with cell cycle regulation (previously described in relation to tumor suppression) as well as several host genes involved in innate immunity and/or inflammation to be associated with peripheral measures of the HIV DNA and RNA reservoirs, respectively. Several of the inflammatory and innate immune genes and pathways associated with HIV usRNA are highly interrelated signaling pathways related to pathogen recognition (TLRs, NLRs), inflammasome activation (IL-1, IL-6), mucosal homeostasis (IL-10, TLR4), and inflammation (TNF-α, chemokine release). In addition, we report two novel associations with genes encoding for membrane channel proteins that may play a role in these same inflammatory pathways. A limited set of protein validation assays were performed which showed that the association between IL-10 and TNF-α was also significantly inversely associated with HIV usRNA. These discovery-based transcriptomic findings add to the limited host genetic and HIV reservoir literature and suggest that while changes in host gene expression may influence the size of the HIV reservoir, host gene expression itself may in turn, vary in response to the transcriptionally active reservoir. Additional studies in larger cohorts are needed to further validate these findings.

## Materials and methods

### Study participants

HIV+ ART-suppressed non-controllers from the University of California San Francisco (UCSF) SCOPE and Options HIV+ cohorts were included in the study. The UCSF SCOPE and Options cohorts are prospective clinic-based cohort studies, currently with over 2500 participants to date, and are enriched for elite controllers (ECs) and post-treatment controllers (PTCs) as part of the ongoing aims (NCT00187512). Detailed interviews are conducted every 4 months, including questions regarding medications, medication adherence, and intercurrent illnesses. At each study visit, plasma HIV-1 RNA levels and CD4+ T cell counts are measured, as well as PBMC and plasma collected. Using strict clinical and laboratory criteria, Elite Controller and/or Post-Treatment Controller status is defined (e.g., for Elite Controller- Confirmed: “antiretroviral therapy-naïve subjects who have at least one year duration of documented viral loads that are below the level of detection on conventional assays up until the date of the selected specimen”). Inclusion criteria were laboratory-confirmed HIV-1 infection, availability of cryopreserved peripheral blood mononuclear cells (PBMCs), and plasma HIV RNA levels below the limit of assay quantification for at least 24 months at the time of biospecimen collection. We excluded individuals with elite or post-treatment control (as defined by clinical and laboratory data), recent hospitalization, infection requiring antibiotics, vaccination, or exposure to immunomodulatory drugs in the six months prior to sampling timepoint. The research was approved by the University of California San Francisco Committee on Human Research (CHR), and all participants provided written informed consent.

### HIV reservoir quantification

Cryopreserved PBMCs were viably thawed and total CD4+ T cells were enriched via immunomagnetic selection (StemCell, Vancouver, Canada), with cell count, viability, and purity assessed by flow cytometry both before and after selection. DNA and RNA were extracted from CD4+ T cells using the AllPrep Universal Kit (Qiagen, Hilden, Germany). Quantitative PCR was performed to determine the levels of HIV-1 cell-associated RNA (HIV usRNA), proviral DNA (HIV totalDNA), and CCR5 in each subgroup. CCR5 was used to calculate assay cell concentration and extraction efficiency. Primer pairs and probe sequences were used as described in [44,180]. Briefly, the same primer and probe sequences were used for both total HIV-1 DNA and unspliced RNA and sit near the Gag-LTR junction, a highly conserved region among all group M HIV-1 sequences. PCR reactions were performed on an ABI OneStep qPCR machine (Applied Biosystems) using either the ABI TaqMan Universal PCR Master Mix for DNA or the ABI TaqMan Fast Virus 1-Step Master Mix for RNA for up to 45 cycles as we have previously described [44].

Using the remaining extracted DNA from the CD4+ T cells, we performed a multiplexed ddPCR assay targeting three regions of the HIV-1 genome to quantify the frequency of cells with “intact” HIV (a proxy for the frequency of replication-competent provirus) [31]. HIV intact DNA was quantified by targeting five regions on the HIV genome, including highly conserved regions and positions that are frequently deleted or hypermutated [31]. Optimized restriction enzyme digestion was used to prepare the genomic DNA for droplet formation while minimizing the amount of shearing within the viral genome. The protocol targeted 5 regions in the HIV genome across two triplex droplet digital PCR (ddPCR) reactions. Droplet generation and thermocycling were performed according to manufacturer instructions. Thus, the multiplex ddPCR assay allowed the analysis of potentially replication-competent (“intact”) proviral genomes by quantifying the number of droplets positive for 3 targets per reaction. Two targets in a housekeeping gene (*RPP30*) were used to quantify all cells, and a target in the T cell receptor D gene (*TRD*) was used to normalize the HIV copy numbers per 1x10^6^ CD4+ T cells. A DNA shearing index (DSI) was then calculated, and mathematically corrected for residual DNA shearing as measured by *RPP30* targets to calculate the estimated number of intact proviral genomes per million CD4+ T cells after correcting for shearing [87]. As previously described, we employed a conservative approach of assigning a zero value for HIV intact DNA if one of two HIV-1 reactions were undetectable [31,87], since even one reaction with defective proviral sequence suggests non-intact HIV DNA.

### Host RNA sequencing

As above for the HIV reservoir quantification, cryopreserved PBMCs were enriched for CD4+ T cells (StemCell, Vancouver, Canada), and RNA was extracted from CD4+ T cells using the AllPrep Universal Kit (Qiagen, Hilden, Germany) with one aliquot set aside for HIV reservoir quantification and a second aliquot for host RNA sequencing. RNA was quantified using Qubit 2.0 Fluorometer (Thermo Fisher Scientific, Waltham, MA, USA) and integrity checked using Tape Station (Agilent Technologies, Palo Alto, CA, USA). Standard RNA sequencing libraries were prepared using the NEBNext Ultra II RNA Library Prep Kit for Illumina using manufacturer’s instructions (New England Biolabs, Ipswich, MA, USA). Briefly, mRNAs were initially enriched with Oligod(T) beads and then fragmented for 15 minutes at 94°C. First strand and second strand cDNA were subsequently synthesized, end repaired and adenylated, and universal adapters were ligated to cDNA fragments, followed by index addition and library enrichment by PCR. For a subset of samples, ultra-Low RNA sequencing libraries were prepared with SMART-Seq v4 Ultra Low Input Kit for Sequencing for full-length cDNA synthesis and amplification (Clontech, Mountain View, CA), and Illumina Nextera XT library was used for sequencing library preparation. Briefly, cDNA was fragmented, and adaptor was added using Transposase, followed by limited-cycle PCR to enrich and add index to the cDNA fragments. Sequencing libraries were validated using the Agilent Tape Station (Agilent Technologies, Palo Alto, CA, USA), and quantified using Qubit 2.0 Fluorometer (Thermo Fisher Scientific, Waltham, MA, USA). The sequencing libraries were then clustered on flowcell lanes using the Illumina NovaSeq6000 instrument according to manufacturer’s instructions and sequenced using a 2x150bp Paired End (PE) configuration. Image analysis and base calling were conducted by the NovaSeq Control Software (allowing for single mismatch for index sequence identification), and raw sequence data (.bcl files) generated from Illumina NovaSeq was converted to fastq files and de-multiplexed using Illumina’s bcl2fastq 2.19 software.

The HTStream pre-processing pipeline (s4hts.github.io/htstream/) was used for removing PCR duplicates, adapters, N characters, PolyA/T sequences, Phix contaminants, and poor-quality sequences (with quality score <20 with sliding window of 10 base pairs). The quality of raw reads was assessed using FastQC [181]. All samples had a per base quality score and sequence quality score >30. RNA-seq reads were then mapped to the human genome (GRCh38) [182] with a corresponding annotation file from the GENCODE project [183]. Alignment and gene quantification were performed using the STAR alignment tool and its quantification protocols [184–186]. Gene expression was converted to counts per million (CPM). To normalize the distribution of expression values across the experiment, the trimmed mean of M-values (TMM) [60] was used for sample-specific adjustment. Low-expressed genes (<1 CPM for all samples) were removed. The mean-variance trend was estimated [61] to assign observational weights based on predicted variance on log_2_-counts per million (log_2_-CPM) using the Limma-Voom pipeline [62]. After QC analyses, a total of 19,912 genes out of 60,719 were included for downstream differential gene expression analyses.

### Differential gene expression analysis

Multivariate linear models were fit for each of the three HIV reservoir measures using the Limma-Voom workflow [61,62], a quantitative weighting method that utilizes variance modeling to accommodate for residual technical and/or biological heterogeneity [61]. For all analyses, in order to account for potential population stratification bias (i.e., systematic differences in results due to ancestry rather than association of genes with disease) we used well-established methods to account for this by (1) calculating and including the first five principal components (PCs) as covariates in the multivariate models [36] and (2) performing sensitivity analyses among the largest subgroup, individuals of European ancestry. Eigenvalues were calculated to generate genetic principal components (PCs) [36] to adjust for ancestry [37]. Inclusion of potential clinical covariates for sex, age, timing of ART initiation (estimated days from HIV acquisition to ART initiation), nadir CD4+ T cell count, duration of ART suppression, and maximum pre-ART viral load were tested in multivariate models. PEERs (probabilistic estimation of expression residuals) were tested for inclusion in multivariate models to control for additional systematic sources of bias [45]. Model fit was assessed using a lambda genomic coefficient close to 1 [187]. Statistical significance was determined using a false discovery rate (FDR) q-value threshold of <0.05. Data were analyzed and visualized (ggplot2) using the R studio statistical package (2023.06.1 Build 524).

### Pathway-based analyses

For each of the three HIV reservoir measures, we also performed pathway-based analyses to more broadly evaluate whether specific immune pathways were linked to each HIV reservoir measurement. Genes from the entire transcriptome were first rank-ordered by q-values from the differential gene expression analysis for each HIV reservoir measure, and then the rank-ordering was used to identify immunologic pathways that were enriched from our dataset, using the Gene Ontology Biological Processes (GO-BP) database [188]. For the HIV usRNA analyses, for which there were several statistically significant differentially expressed genes (even after multiple testing), we performed network analyses to better cluster and visualize the statistically significant results. Using ClueGo, a network analysis application [67], only statistically significant and marginally significant genes (q<0.25) were included to calculate Kappa statistics that allowed more meaningful visualization of potential biologically relevant pathways (**Fig 4**).

### Protein validation sample selection and statistical analyses

For validation of intracellularly expressed or membrane-associated encoded proteins, we performed ELISA from peripheral CD4-enriched T cells. We were able to obtain an additional PBMC aliquot of 10 million cells for 40 of our 191 study participants. For validation of the several inflammatory pathway genes identified in association with HIV usRNA, most of the encoded proteins were secreted proteins, and thus, we performed high-sensitivity multiplex plasma cytokine quantification (Meso Scale Diagnostics) from 175 of the 191 study participants for whom we had available matched plasma. After customization, we were able to include seven proteins from our analyses (G-CSF, IP-10, TNFAIP5, IL-1β, IL-10, TNF-α, and sTLR4). For the protein validation analyses, given the smaller sample size, we first performed Spearman correlations between the protein expression levels and corresponding gene expression levels. Next, we performed Spearman correlations between each protein level and the associated HIV reservoir measures. Several of the protein levels demonstrated a small number of individuals with outlier values; we performed sensitivity analyses with and without outliers for each protein assayed. Multivariate models for each protein were fit as described above for the RNA-seq analyses, including significant covariates for nadir CD4+ T cell count and timing of ART initiation for protein analyses. Linear spline models as well as nonlinear general additive models were fit; results were similar and therefore, linear model estimates are presented in the final tables given the enhanced interpretability of these results (e.g., fold-change in host protein expression per two-fold change in copies of HIV). Data were analyzed and visualized (ggplot2) using the R studio statistical package (2023.06.1 Build 524).

### Preparation of cellular lysates from purified CD4+ T cells

CD4+ T cells were isolated from PBMC by negative selection, using the EasySep Human CD4+ T Cell Isolation Kit (StemCell Technologies, Vancouver, BC, Canada), following manufacturer’s guidelines. Purified CD4+ cells were resuspended in 250 μl of PBS supplemented with 2% heat-inactivated fetal bovine serum, 1 mM EDTA and a cocktail of protease inhibitors (Pierce, ThermoFisher Scientific). The Muse Human CD4 T Cell kit (Luminex, Austin, TX) in combination with the Guava Muse Cell Analyzer were used to determine the concentration and percentages of CD4+ T cells after the isolation. The mean purity (% CD4+) was 96.95% (range: 88.95%-99.33%, N = 40 samples) and the mean total number of CD4+ cells was 2.41x106 (range: 6.07x105- 5.89x10^6^, N = 40 samples). To generate cellular lysates, purified CD4+ T cells were subjected to three cycles of freezing/thawing, using a dry ice (frozen CO2) /absolute ethanol mixture and a 37°C water bath. Complete lysis was verified by trypan blue staining and microscopic analysis. Lysates were spun down at 1,500 g for 10 min at 4°C (to remove cellular debris) and the supernatants diluted 1:5 with PBS and kept at -80°C until the time of protein quantification.

### Quantification of intracellular protein markers in purified CD4^+^ T cells

Total protein concentration of the CD4^+^ T cell lysates was determined using the Pierce BCA assay (Thermo Fisher Scientific). The mean value obtained was 0.94 mg/ml (range: 0.66 mg/ml- 1.18 mg/ml). The list below enumerates the assays used to determine intracellular concentration of the protein markers described in the text: Human NBL1 / DAN (CLIA) ELISA Kit (LSBio, LS-F25869); Human P3H3 / LEPREL2 Sandwich ELISA Kit (LSBio, LS-F74784); KCNJ2, Human Inward rectifier potassium channel 2 ELISA Kit (MyBioSource, MBS9305840); Human Connexin 26/GJB2 ELISA Kit (Novus Biologicals, NBP2-79811) and Human TLR4 (CLIA) ELISA Kit (LSBio, LS-F29972).

Chemiluminescence or absorbance was read on a SpectraMax iD5 multi-mode plate reader (Molecular Devices, San Jose, CA) and reported in relative light units (RLU). A standard curve was constructed by plotting the log mean RLU reading for each standard on the y-axis against the log of known concentrations on the x-axis using the SoftMax Pro 7.1 software (Molecular Devices, San Jose, CA). Data were normalized by total protein concentration to accurately reflect the total population of cells (live and dead). Briefly, a 1:5 dilution factor (based on supernatant dilution with PBS at the time of CD4+ T cell isolation) was used to calculate the concentration in the lysate before the dilution. Each protein marker was then quantified using the marker-specific ELISA (again, taking into account the 1:5 dilution performed before cryopreservation of the lysates). Data normalization was performed by dividing the concentration of each protein in the final lysate (e.g., for P3H3 in pg/ml) by the total protein concentration (mg/ml).

### Quantification of plasma cytokines by MSD

Plasma levels of IP-10 (the encoded protein for *CXCL10*), G-CSF (*GCSF*) and pentraxin 3 (*TNFAIP5*) were quantified using the electrochemiluminescence-based 3-plex mesoscale discovery (MSD) platform (U-Plex mesoscale discovery, Rockville, MA); IL-10 (*IL10*), IL-1β (*IL1B*) and TNF-α (*TNFA*) were measured in a separate 3-plex S-plex Proinflammatory panel kit, and IL-1α (*IL1A*) was quantified by a V-plex kit. In all these assays, undiluted samples were run in duplicate following manufacturer’s instructions, and protein concentrations were determined using MSD Discovery Workbench (version 4.0.13) analysis software. The light intensities from the samples were interpolated using a four-parameter logistic fit (FourPL) to a standard curve of electrochemiluminescence generated from eight calibrators of know concentrations. The lower limit of detection for each marker can be found on the manufacturer’s website (MesoScale Diagnostics, https://www.mesoscale.com/~/media/files/handout/assaylist.pdf).

### Quantification of soluble TLR4 (sTLR4) in plasma by ELISA

ELISA analysis of plasma samples was performed using a human TLR4 CLIA (chemiluminescent based) kit (LSBio, Seattle, WA). TLR4 standards were made in a 1:2 dilution using the calibrator and sample diluent. The assay was performed following manufacturer’s instructions. Chemiluminescence was read on a SpectraMax iD5 multi-mode plate reader (1 sec/well, read height: 1 mm, Molecular Devices, San Jose, CA) and reported in relative light units (RLU). A standard curve was constructed by plotting the log mean RLU reading for each standard on the y-axis against the log of known concentrations on the x-axis using the SoftMax Pro 7.1 software (Molecular Devices, San Jose, CA).

## Supporting information

S1 Fig Study participant sample selection flowchart. Specific inclusion and exclusion criteria are listed for each selection step and for HIV reservoir measure analysis. CPM = counts per million.
(PDF)

S2 Fig Principal component analysis (PCA) plot of the population structure, based on our previously published host DNA exome data [37]. Principal component analysis (PCA) plot of the population structure of the full study cohort (a). Secondary PCA plot of the European ancestry subpopulation only (b) defined by the dashed box in the lower left of panel (a). Genetic PCs were calculated from genetic data from our whole exome analysis. Most of the population was of European ancestry (bottom left of) (a) some continued variability. Some continued variability was observed in European ancestry subgroup (b). Self-identified race/ethnicity shown in the legend. Frequencies for participants were recorded as: White/European American (62%), Black/African American (14%), Hispanic/Latino (11%), Mixed Ethnicity/Multiracial (6%), Asian (4%), Pacific Islander (2%), Native American (<1%), and Middle Eastern (<1%).
(PDF)

S3 Fig Spearman correlations between three HIV reservoir measures performed from peripheral CD4+ T cells of 191 ART-suppressed people living with HIV: HIV total DNA (A, C), unspliced RNA (A-B), intact DNA (B-C).
(PDF)

S4 Fig Spearman correlations of timing of ART initiation (years from estimated infection date to ART start date) and three HIV reservoir measures performed from peripheral CD4+ T cells of 191 ART-suppressed people living with HIV: total DNA (A), unspliced RNA (B), and intact DNA (C).
(PDF)

S5 Fig Spearman correlations of nadir CD4+ T cell count and three HIV reservoir measures performed from peripheral CD4+ T cells of 191 ART-suppressed people living with HIV: total DNA (A), unspliced RNA (B), and intact DNA (C).
(PDF)

S6 Fig Correlations between gene and protein expression for host genes that were associated with HIV Total DNA (*P3H3*, *NBL1*). Spearman correlations between host gene (normalized counts) are shown in relation to protein expression from peripheral CD4+ T cells among a subset of 40 participants in the study.
(PDF)

S7 Fig Correlations between gene and protein expression for host genes that were associated with HIV usRNA (*IL10*/IL-10, *TNFA*/TNF-a, *IL1B*/IL-1b, *CSF3*/G-CSF, *CXCL10*/IP-10, *TNFAIP5*/PTX3, *TLR4*/sTLR4). Spearman correlations between host gene (normalized counts) are shown in relation to plasma protein expression among a subset of 175 participants in the study.
(PDF)

S8 Fig Plasma cytokine expression of G-CSF, IP-10, TNFAIP5, IL-1b, IL-10, TNF-a, and sTLR4. The association between plasma protein expression among 175 study participants in relation to measures of HIV total DNA. While these immunologic pathways were identified in relation to HIV usRNA (Table 3), we were able to compare plasma cytokines in relation to HIV total DNA as an additional analysis.
(PDF)

S9 Fig Correlations between gene and protein expression for host genes that were associated with HIV usRNA (*KCNJ2*, *GJB2*). Spearman correlations between host gene (normalized counts) are shown in relation to protein expression from peripheral CD4+ T cells among a subset of 40 participants in the study.
(PDF)

S10 Fig HIV intact DNA quantification was correlated with sample DNA concentrations. Low levels of detection of HIV intact DNA by ddPCR can be influenced by low sample input DNA concentration, primer-mismatches of HIV-1 sequences, and/or misclassification of “intact” versus “defective” provirus [96]. HIV intact DNA was undetectable in 48% of our measured samples (while for example, total DNA by qPCR was measurable in 95% of samples, S3 Fig).
(PDF)

S11 Fig Proposed model for the inverse association between HIV unspliced RNA and host gene expression in our cross-sectional study of 191 ART-suppressed people living with HIV. Within bulk peripheral CD4+ T cells, higher transcriptional reservoir activity (HIV usRNA) from HIV+ cells (left) may chronically lead to downregulation of host genes encoding for membrane channel proteins involved in HIV-1 entry and release (*KCNJ2*) and cell-cell communication (*GJB2*) in bystander cells (right) in an attempt to suppress persistent cell-cell infection during chronic HIV. Created with BioRender.com.
(PDF)

S1 Table Gene set enrichment analyses (GSEA) of ranked differentially expressed genes in relation to HIV total DNA using the Gene Ontology Biological Processes (GO-BP) database. Genes sets with Benjamini-Hochberg false discovery rate (FDR)-adjusted q<0.25 are shown for the total study population (top panel) and for the European ancestry subgroup (bottom panel). Gene sets where q<0.05 are shown in bold font.
(PDF)

S2 Table Multivariate models of P3H3 and NBL1 protein expression from peripheral CD4+ T cells in relation to HIV total DNA among 40 participants. (PDF)

S3 Table Differentially expressed host genes associated with HIV unspliced RNA (usRNA) in the total study population (top panel) and the European ancestry subgroup (bottom panel), at a Benjamini-Hochberg false discovery rate (FDR) of q<0.25, that were not shown in Table 2. (PDF)

S4 Table Gene set enrichment analyses (GSEA) of ranked differentially expressed genes in relation to HIV unspliced RNA using the Gene Ontology Biological Processes (GO-BP) database. Genes sets with Benjamini-Hochberg false discovery rate (FDR)-adjusted q<0.05 are shown for the total study population (top panel) and for the European ancestry subgroup (bottom panel). Gene sets where q<0.05 are shown in bold font.
(PDF)

S5 Table Multivariate models of plasma IL-1β, IL-10, TNF-α, G-CSF, IP-10, TNFAIP5, and sTLR protein expression in relation to HIV unspliced RNA among 175 participants. (PDF)

S6 Table Multivariate models of Kir2.1 (*KCNJ2*) and Connexin 26 (*GJB2*) protein expression from peripheral CD4+ T cells in relation to HIV unspliced RNA among 40 participants. (PDF)

S7 Table Differentially expressed host genes in relation to HIV Intact DNA in the Total Study Population (top panel) and the European ancestry subgroup (bottom panel), at a Benjamini-Hochberg false discovery rate (FDR) of q<0.25. Two-fold higher level of HIV intact DNA was associated with upregulation of genes involved in glycogen degradation (*AGL*) and inhibits thrombus (clot) degradation (*PLGLB1*).
(PDF)

S8 Table Gene set enrichment analyses (GSEA) of ranked differentially expressed genes in relation to HIV intact DNA using the Gene Ontology Biological Processes (GO-BP) database. Genes sets with Benjamini-Hochberg false discovery rate (FDR)-adjusted q<0.25 are shown for the total study population (top panel) and for the European ancestry subgroup (bottom panel). Gene sets where q<0.05 are shown in bold font.
(PDF)
