# Supplementary material for: A cohort-based study of host gene expression: tumor suppressor and innate immune/inflammatory pathways associated with the HIV reservoir size
Source: PLoS Pathog. 2023 Nov 29;19(11):e1011114. doi: 10.1371/journal.ppat.1011114 (PMC10712869; doi:10.1371/journal.ppat.1011114)

S1 Fig. Study participant sample selection flowchart. Specific inclusion and exclusion criteria are listed for each selection step and for HIV reservoir measure analysis. CPM = counts per million.

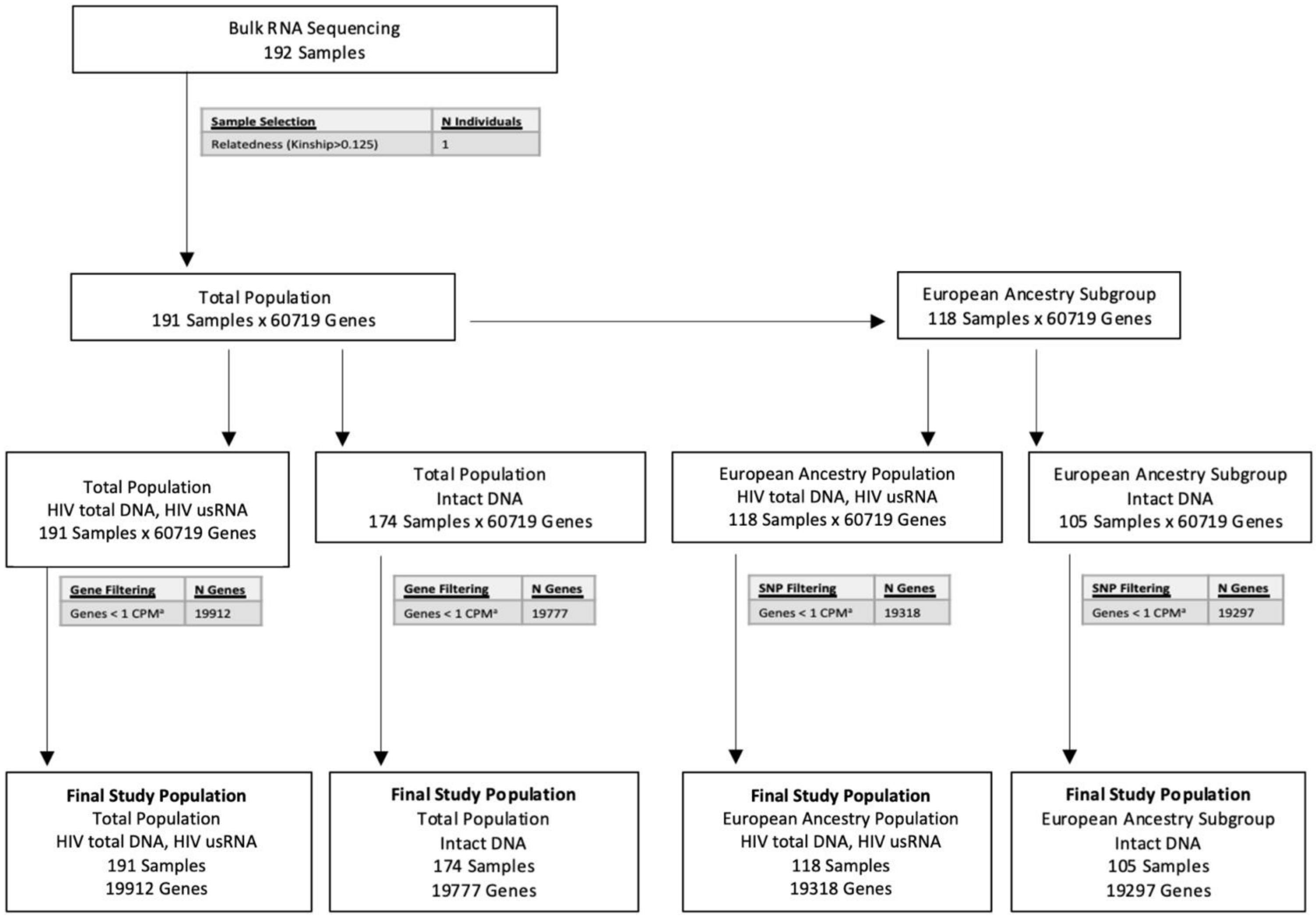

Supplement: S1 Fig — CPM = counts per million. (PDF) [file ppat.1011114.s001.pdf]
