# Supplementary material for: A cohort-based study of host gene expression: tumor suppressor and innate immune/inflammatory pathways associated with the HIV reservoir size
Source: PLoS Pathog. 2023 Nov 29;19(11):e1011114. doi: 10.1371/journal.ppat.1011114 (PMC10712869; doi:10.1371/journal.ppat.1011114)

S2 Fig. Principal component analysis (PCA) plot of the population structure, based on our previously published host DNA exome data [37]. Principal component analysis (PCA) plot of the population structure of the full study cohort (a). Secondary PCA plot of the European ancestry subpopulation only (b) defined by the dashed box in the lower left of panel (a). Genetic PCs were calculated from genetic data from our whole exome analysis. Most of the population was of European ancestry (bottom left of) (a) some continued variability. Some continued variability was observed in European ancestry subgroup (b). Self-identified race/ethnicity shown in the legend. Frequencies for participants were recorded as: White/European American (62%), Black/African American (14%), Hispanic/Latino (11%), Mixed Ethnicity/Multiracial (6%), Asian (4%), Pacific Islander (2%), Native American (<1%), and Middle Eastern (<1%).

A.

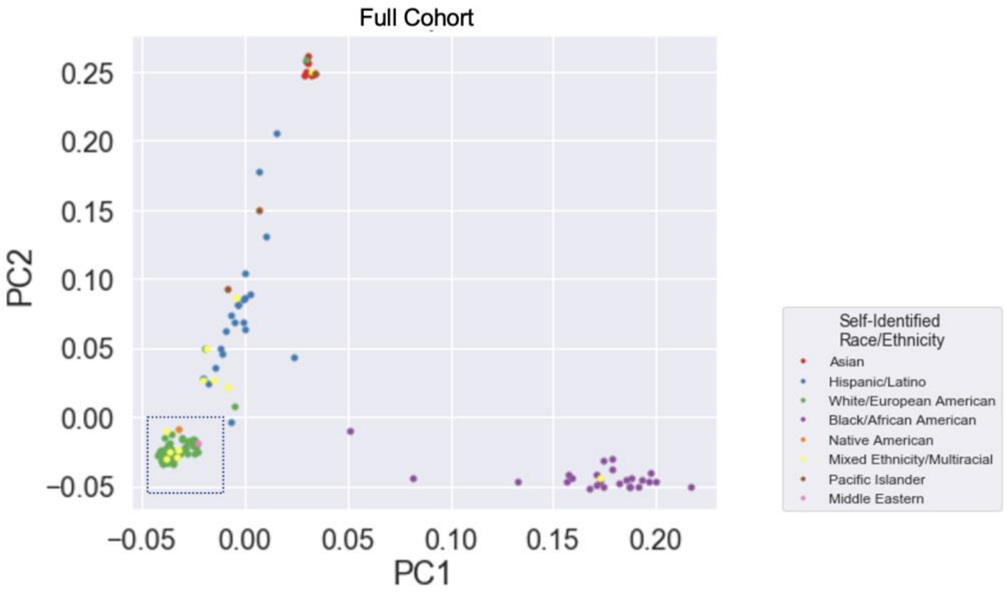

B.

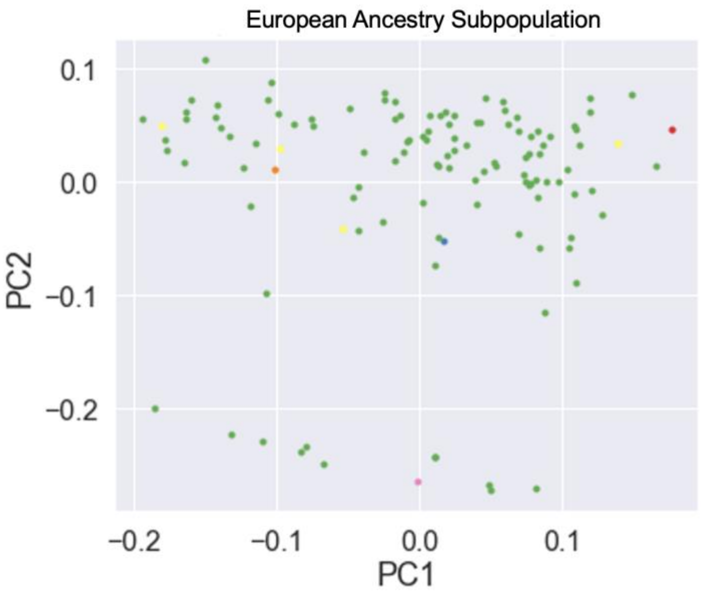

Supplement: S2 Fig — Principal component analysis (PCA) plot of the population structure, based on our previously published host DNA exome data [37]. Principal component analysis (PCA) plot of the population structure of the full study cohort (a). Secondary PCA plot of the European ancestry subpopulation only (b) defined by the dashed box in the lower left of panel (a). Genetic PCs were calculated from genetic data from our whole exome analysis. Most of the population was of European ancestry (bottom left of) (a) some continued variability. Some continued variability was observed in European ancestry subgroup (b). Self-identified race/ethnicity shown in the legend. Frequencies for participants were recorded as: White/European American (62%), Black/African American (14%), Hispanic/Latino (11%), Mixed Ethnicity/Multiracial (6%), Asian (4%), Pacific Islander (2%), Native American (<1%), and Middle Eastern (<1%). (PDF) [file ppat.1011114.s002.pdf]
