# Supplementary material for: A cohort-based study of host gene expression: tumor suppressor and innate immune/inflammatory pathways associated with the HIV reservoir size
Source: PLoS Pathog. 2023 Nov 29;19(11):e1011114. doi: 10.1371/journal.ppat.1011114 (PMC10712869; doi:10.1371/journal.ppat.1011114)

S3 Fig. Spearman correlations between three HIV reservoir measures performed from peripheral CD4+ T cells of 191 ART-suppressed people living with HIV: HIV total DNA (A, C), unspliced RNA (A-B), intact DNA (B-C).

A.

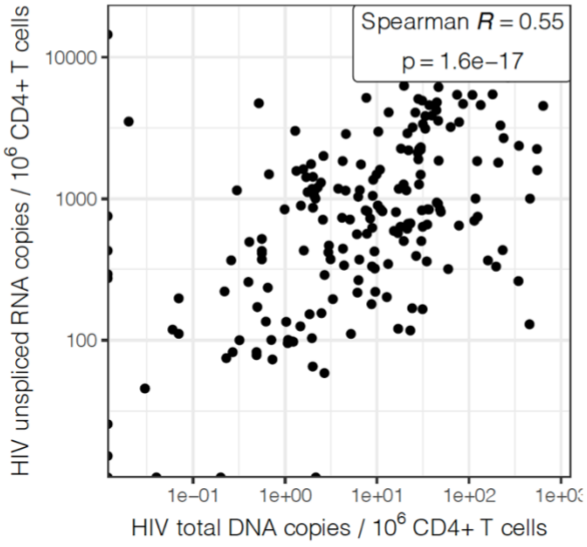

B.

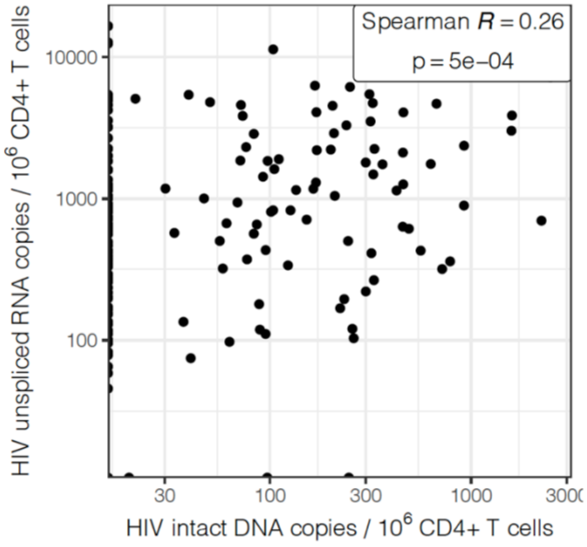

C.

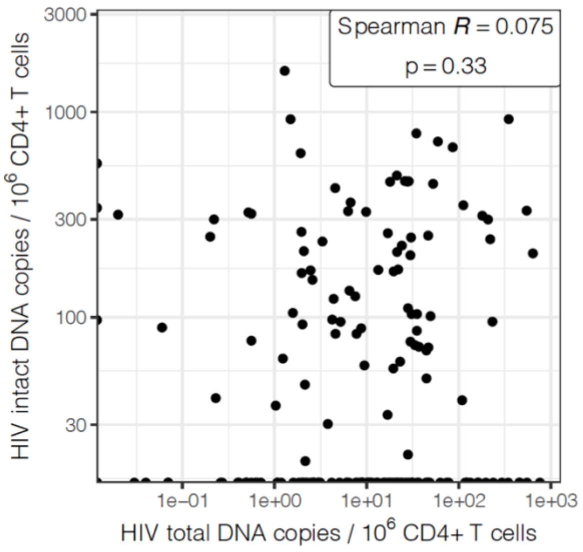

Supplement: S3 Fig — Spearman correlations between three HIV reservoir measures performed from peripheral CD4+ T cells of 191 ART-suppressed people living with HIV: HIV total DNA (A, C), unspliced RNA (A-B), intact DNA (B-C). (PDF) [file ppat.1011114.s003.pdf]
