# Supplementary material for: A cohort-based study of host gene expression: tumor suppressor and innate immune/inflammatory pathways associated with the HIV reservoir size
Source: PLoS Pathog. 2023 Nov 29;19(11):e1011114. doi: 10.1371/journal.ppat.1011114 (PMC10712869; doi:10.1371/journal.ppat.1011114)

S4 Fig. Spearman correlations of timing of ART initiation (years from estimated infection date to ART start date) and three HIV reservoir measures performed from peripheral CD4+ T cells of 191 ART-suppressed people living with HIV: total DNA (A), unspliced RNA (B), and intact DNA (C).

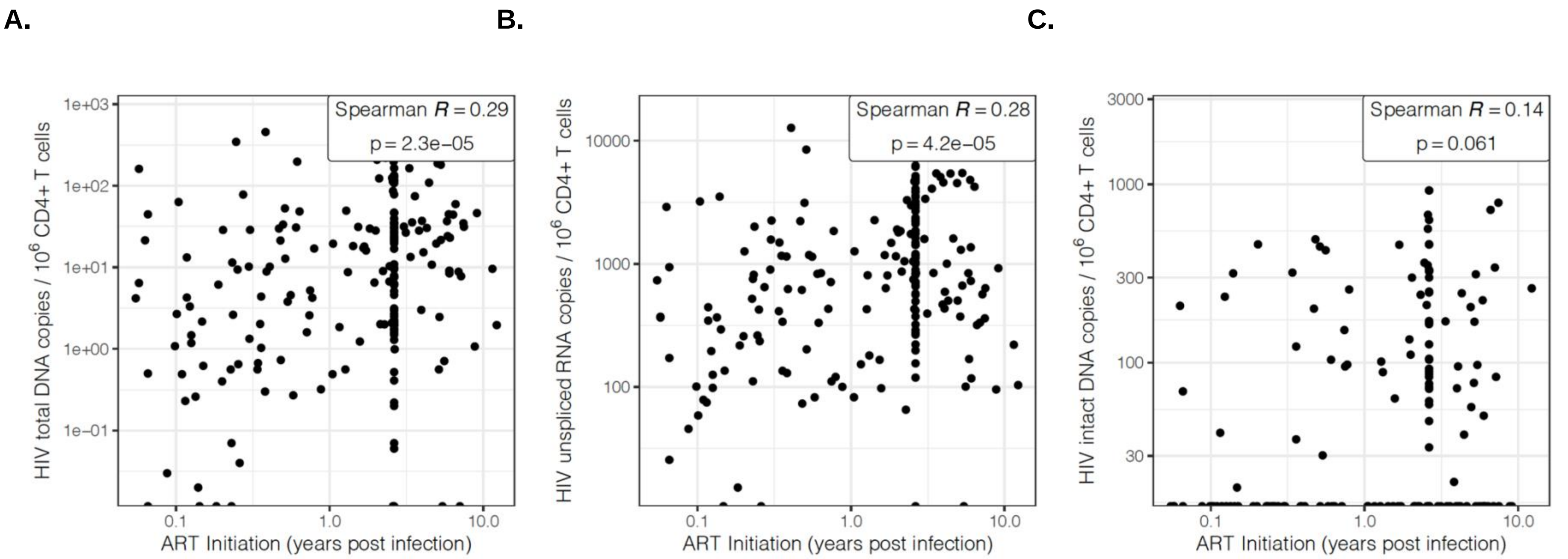

Supplement: S4 Fig — Spearman correlations of timing of ART initiation (years from estimated infection date to ART start date) and three HIV reservoir measures performed from peripheral CD4+ T cells of 191 ART-suppressed people living with HIV: total DNA (A), unspliced RNA (B), and intact DNA (C). (PDF) [file ppat.1011114.s004.pdf]
