# Supplementary material for: A cohort-based study of host gene expression: tumor suppressor and innate immune/inflammatory pathways associated with the HIV reservoir size
Source: PLoS Pathog. 2023 Nov 29;19(11):e1011114. doi: 10.1371/journal.ppat.1011114 (PMC10712869; doi:10.1371/journal.ppat.1011114)

S5 Fig. Spearman correlations of nadir CD4+ T cell count and three HIV reservoir measures performed from peripheral CD4+ T cells of 191 ART-suppressed people living with HIV: total DNA (A), unspliced RNA (B), and intact DNA (C).

A. **B.** **C.**

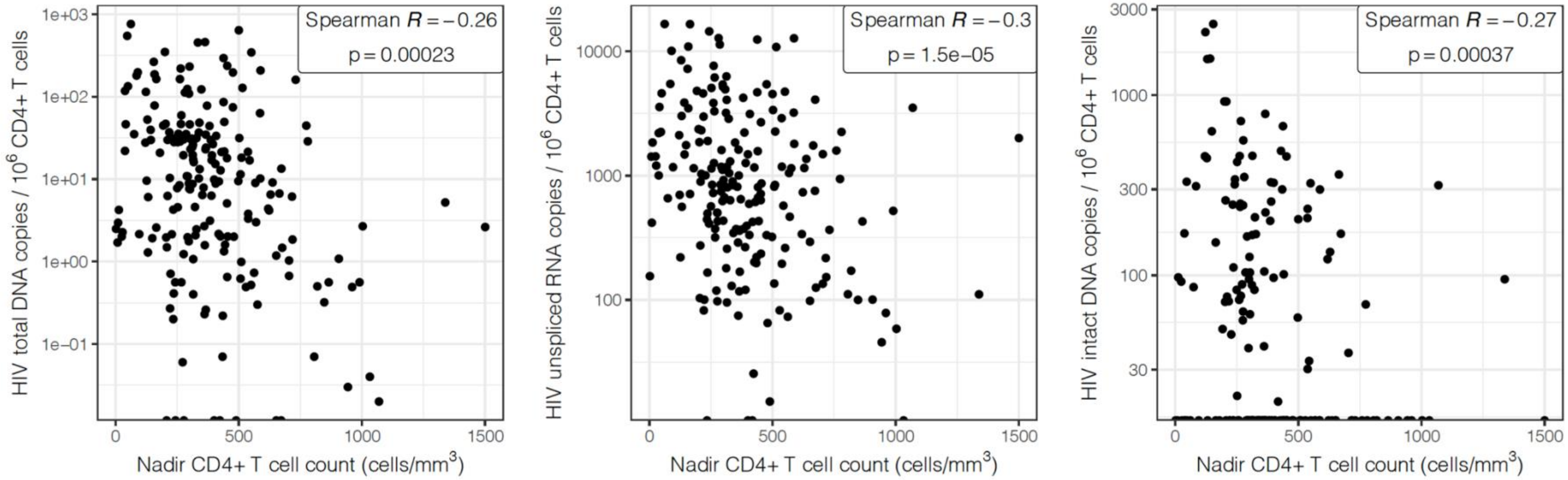

Supplement: S5 Fig — Spearman correlations of nadir CD4+ T cell count and three HIV reservoir measures performed from peripheral CD4+ T cells of 191 ART-suppressed people living with HIV: total DNA (A), unspliced RNA (B), and intact DNA (C). (PDF) [file ppat.1011114.s005.pdf]
