# Supplementary material for: A cohort-based study of host gene expression: tumor suppressor and innate immune/inflammatory pathways associated with the HIV reservoir size
Source: PLoS Pathog. 2023 Nov 29;19(11):e1011114. doi: 10.1371/journal.ppat.1011114 (PMC10712869; doi:10.1371/journal.ppat.1011114)

S6 Fig. Correlations between gene and protein expression for host genes that were associated with HIV Total DNA (*P3H3, NBL1*). Spearman correlations between host gene (normalized counts) are shown in relation to protein expression from peripheral CD4+ T cells among a subset of 40 participants in the study.

A.

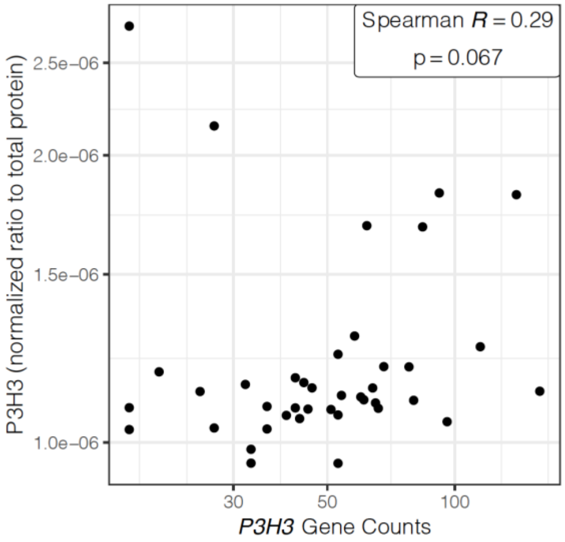

B.

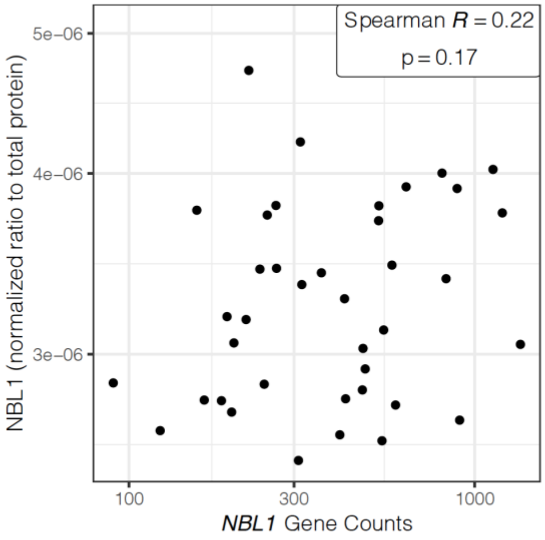

Supplement: S6 Fig — Spearman correlations between host gene (normalized counts) are shown in relation to protein expression from peripheral CD4+ T cells among a subset of 40 participants in the study. (PDF) [file ppat.1011114.s006.pdf]
