# Supplementary material for: A cohort-based study of host gene expression: tumor suppressor and innate immune/inflammatory pathways associated with the HIV reservoir size
Source: PLoS Pathog. 2023 Nov 29;19(11):e1011114. doi: 10.1371/journal.ppat.1011114 (PMC10712869; doi:10.1371/journal.ppat.1011114)
Supplement: S7 Fig — Spearman correlations between host gene (normalized counts) are shown in relation to plasma protein expression among a subset of 175 participants in the study. (PDF) [file ppat.1011114.s007.pdf]

S7 Fig. Correlations between gene and protein expression for host genes that were associated with HIV usRNA (*IL10*/IL-10, *TNFA*/TNF-a, *IL1B*/IL-1b, *CSF3*/G-CSF, *CXCL10*/IP-10, *TNFAIP5*/PTX3, *TLR4*/sTLR4). Spearman correlations between host gene (normalized counts) are shown in relation to plasma protein expression among a subset of 175 participants in the study.

A.

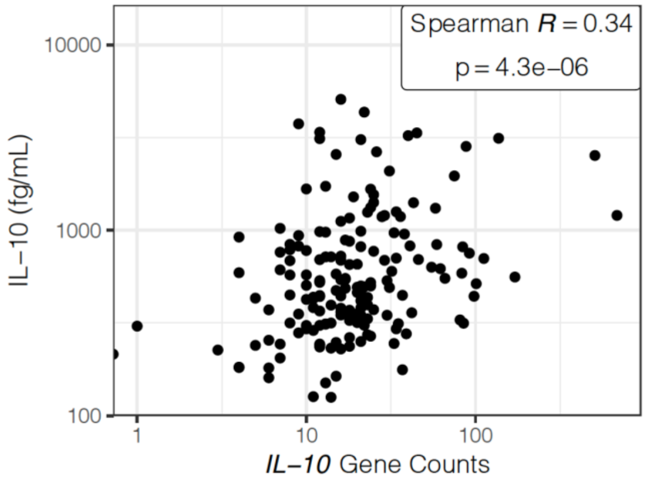

B.

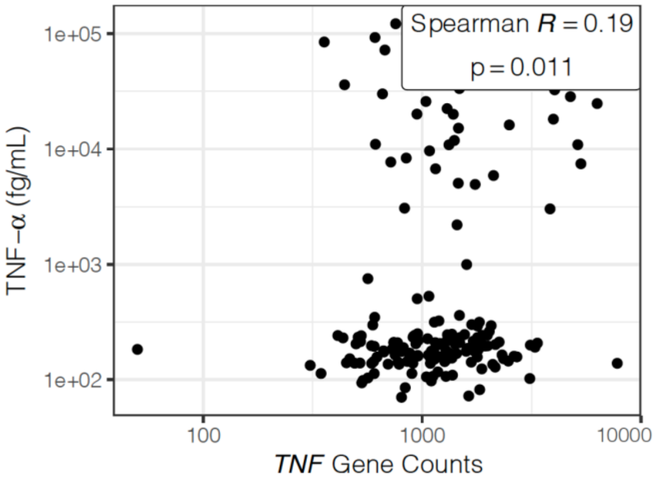

C.

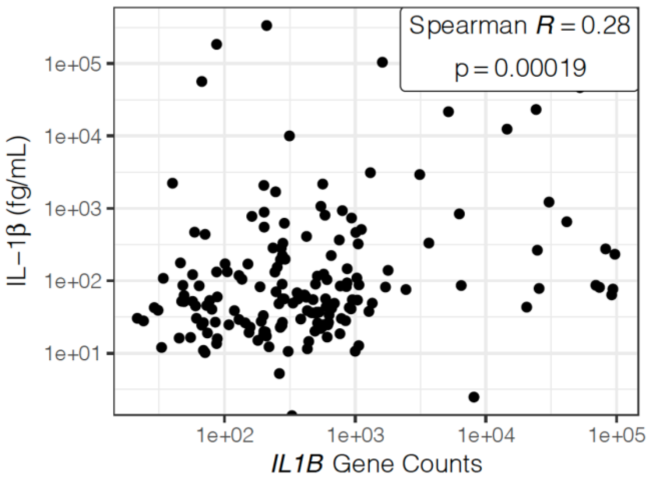

D.

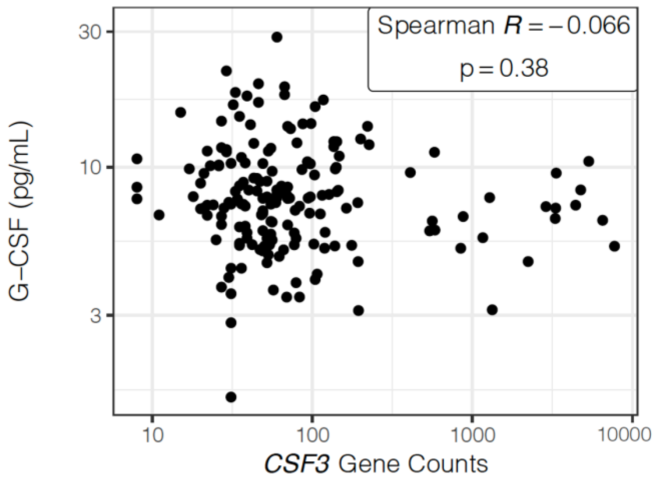

E.

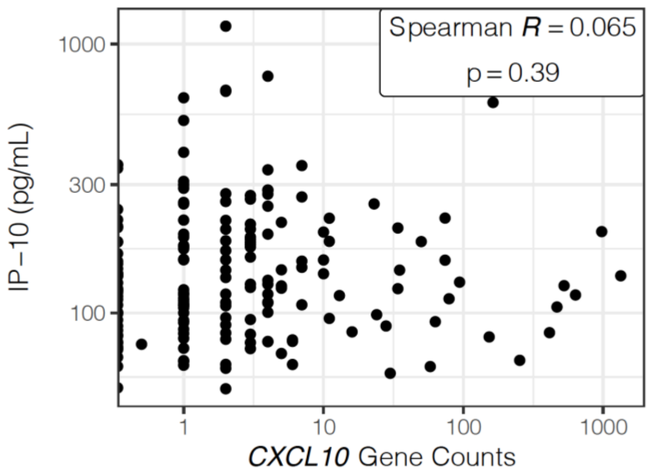
Spearman $R = 0.065$
$p = 0.39$
IP−10 (pg/mL)
1000
300
100
1
10
100
1000
CXCL10 Gene Counts

F.

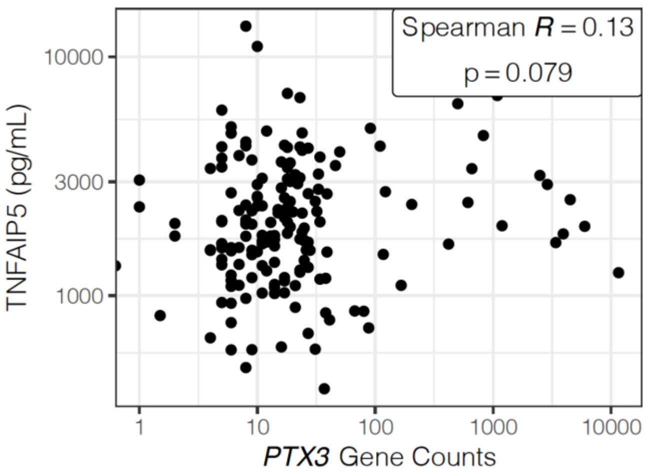
Spearman $R = 0.13$
$p = 0.079$
TNFAIP5 (pg/mL)
10000
3000
1000
1
10
100
1000
10000
PTX3 Gene Counts

G.

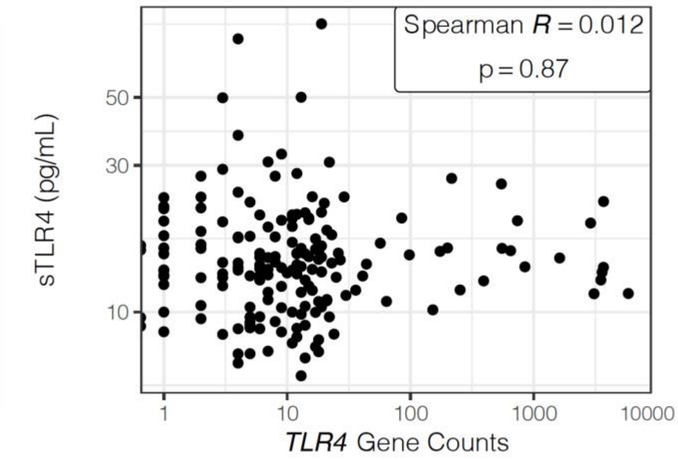
Spearman $R = 0.012$
$p = 0.87$
sTLR4 (pg/mL)
50
30
10
1
10
100
1000
10000
TLR4 Gene Counts
