# Supplementary material for: A cohort-based study of host gene expression: tumor suppressor and innate immune/inflammatory pathways associated with the HIV reservoir size
Source: PLoS Pathog. 2023 Nov 29;19(11):e1011114. doi: 10.1371/journal.ppat.1011114 (PMC10712869; doi:10.1371/journal.ppat.1011114)
Supplement: S8 Fig — The association between plasma protein expression among 175 study participants in relation to measures of HIV total DNA. While these immunologic pathways were identified in relation to HIV usRNA (Table 3), we were able to compare plasma cytokines in relation to HIV total DNA as an additional analysis. (PDF) [file ppat.1011114.s008.pdf]

S8 Fig. Plasma cytokine expression of G-CSF, IP-10, TNFAIP5, IL-1b, IL-10, TNF-a, and sTLR4. The association between plasma protein expression among 175 study participants in relation to measures of HIV total DNA. While these immunologic pathways were identified in relation to HIV usRNA (Table 3), we were able to compare plasma cytokines in relation to HIV total DNA as an additional analysis.

A.

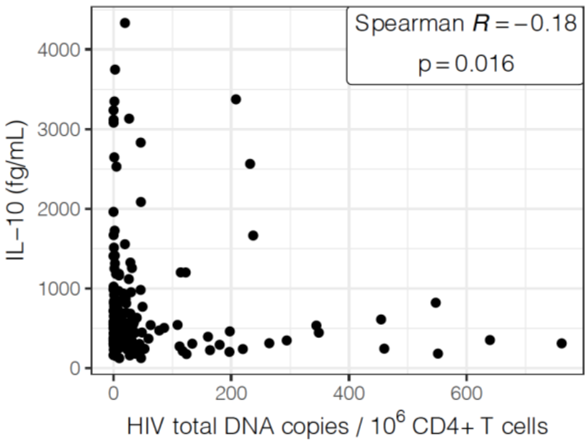

B.

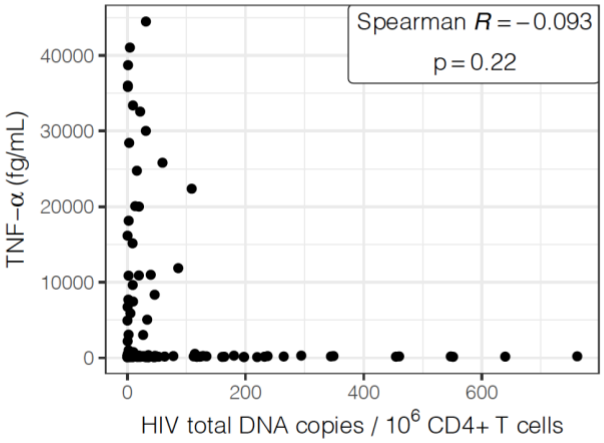

C.

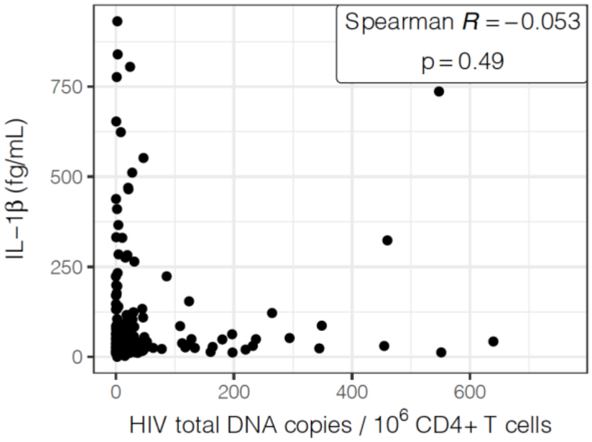

D.

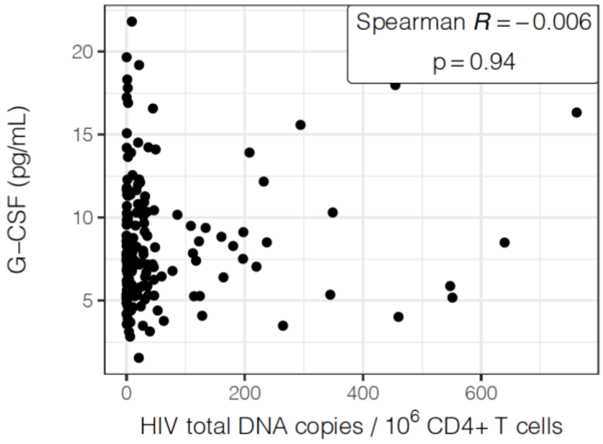

E.

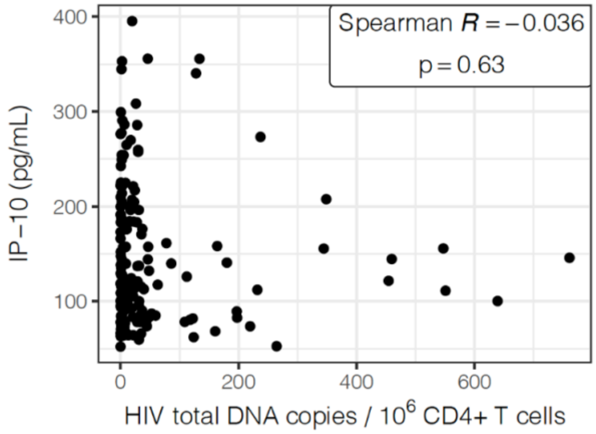
Spearman $R = -0.036$
$p = 0.63$
IP−10 (pg/mL)
400
300
200
100
0
200
400
600
HIV total DNA copies / 10^6 CD4+ T cells

F.

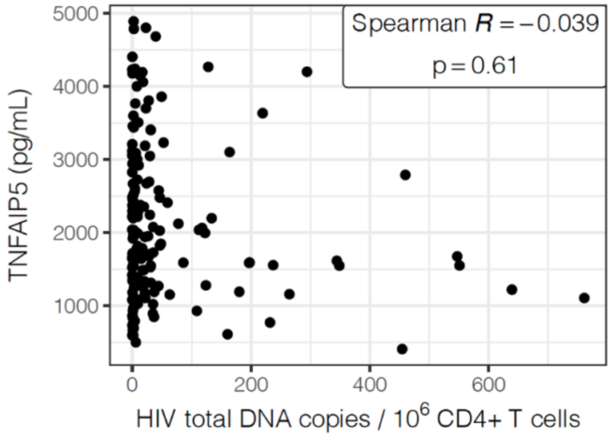
Spearman $R = -0.039$
$p = 0.61$
TNFAIP5 (pg/mL)
5000
4000
3000
2000
1000
0
200
400
600
HIV total DNA copies / 10^6 CD4+ T cells

G.

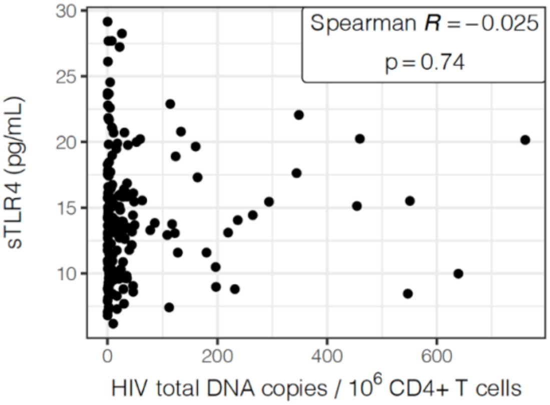
Spearman $R = -0.025$
$p = 0.74$
sTLR4 (pg/mL)
30
25
20
15
10
0
200
400
600
HIV total DNA copies / 10^6 CD4+ T cells
