# Supplementary material for: A cohort-based study of host gene expression: tumor suppressor and innate immune/inflammatory pathways associated with the HIV reservoir size
Source: PLoS Pathog. 2023 Nov 29;19(11):e1011114. doi: 10.1371/journal.ppat.1011114 (PMC10712869; doi:10.1371/journal.ppat.1011114)

S10 Fig. HIV intact DNA quantification was correlated with sample DNA concentrations. Low levels of detection of HIV intact DNA by ddPCR can be influenced by low sample input DNA concentration, primer-mismatches of HIV-1 sequences, and/or misclassification of “intact” versus “defective” provirus [96, 212]. HIV intact DNA was undetectable in 48% of our measured samples (while for example, total DNA by qPCR was measurable in 95% of samples, S3 Fig).

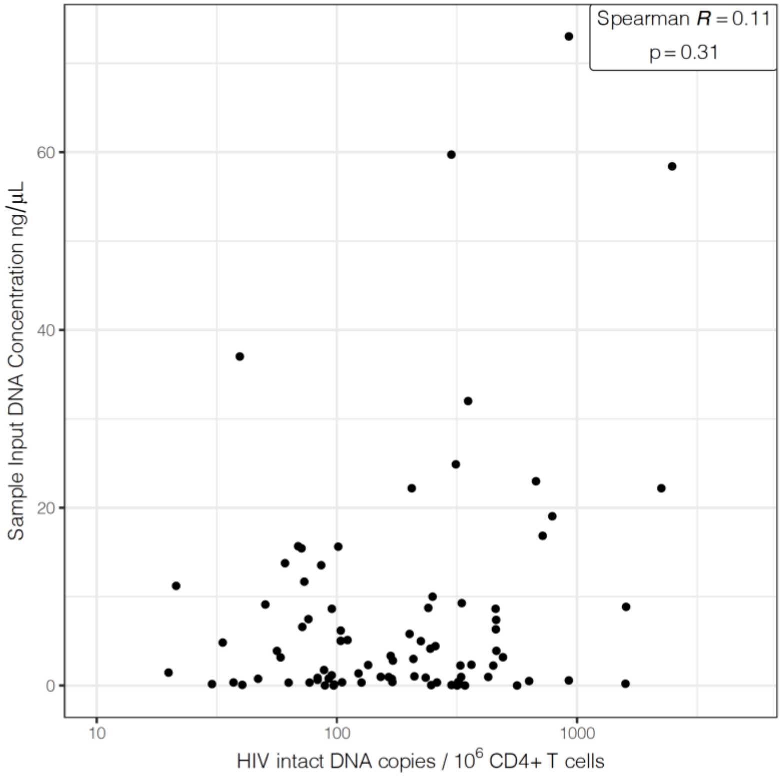

Supplement: S10 Fig — Low levels of detection of HIV intact DNA by ddPCR can be influenced by low sample input DNA concentration, primer-mismatches of HIV-1 sequences, and/or misclassification of “intact” versus “defective” provirus [96]. HIV intact DNA was undetectable in 48% of our measured samples (while for example, total DNA by qPCR was measurable in 95% of samples, S3 Fig). (PDF) [file ppat.1011114.s010.pdf]
