# Supplementary material for: A cohort-based study of host gene expression: tumor suppressor and innate immune/inflammatory pathways associated with the HIV reservoir size
Source: PLoS Pathog. 2023 Nov 29;19(11):e1011114. doi: 10.1371/journal.ppat.1011114 (PMC10712869; doi:10.1371/journal.ppat.1011114)

S11 Fig. Proposed model for the inverse association between HIV unspliced RNA and host gene expression in our cross-sectional study of 191 ART-suppressed people living with HIV. Within bulk peripheral CD4+ T cells, higher transcriptional reservoir activity (HIV usRNA) from HIV+ cells (left) may chronically lead to downregulation of host genes encoding for membrane channel proteins involved in HIV-1 entry and release (*KCNJ2*) and cell-cell communication (*GJB2*) in bystander cells (right) in an attempt to suppress persistent cell-cell infection during chronic HIV. Created with BioRender.com.

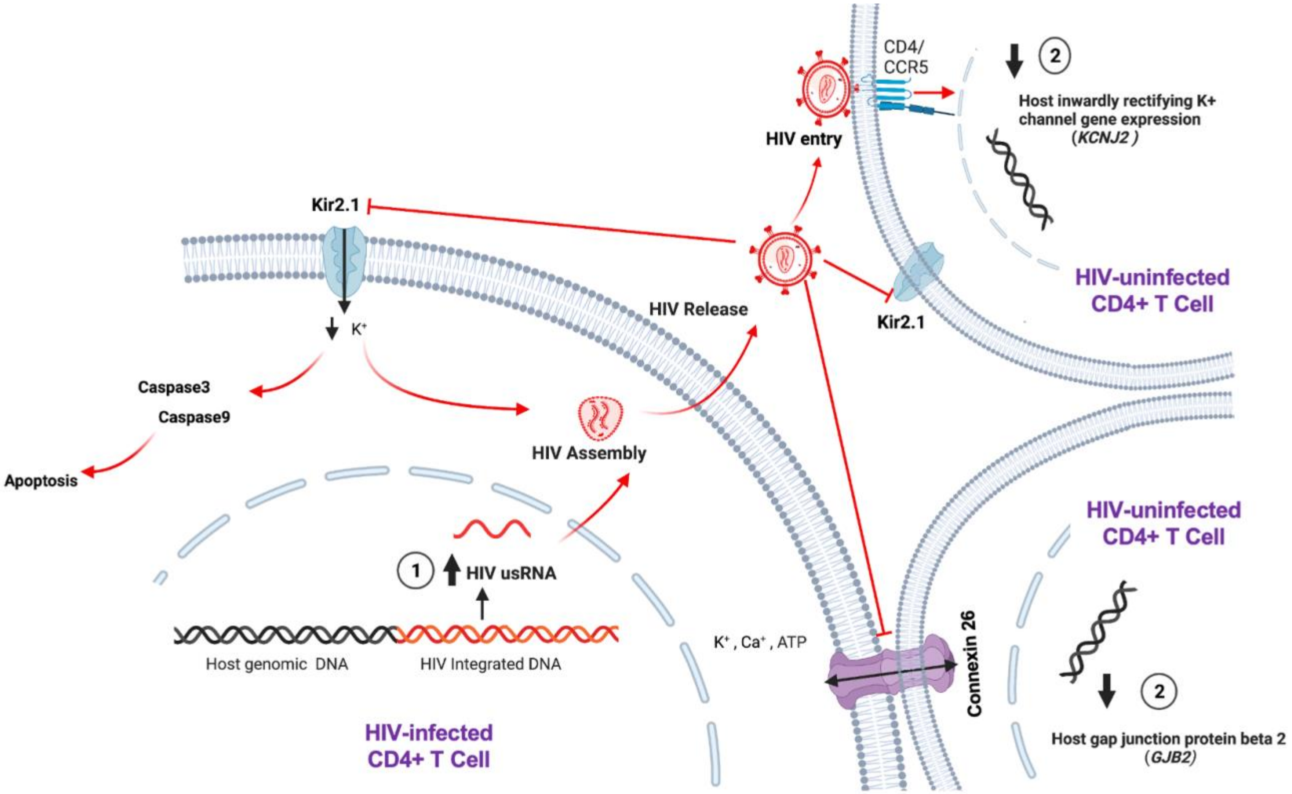

Supplement: S11 Fig — Within bulk peripheral CD4+ T cells, higher transcriptional reservoir activity (HIV usRNA) from HIV+ cells (left) may chronically lead to downregulation of host genes encoding for membrane channel proteins involved in HIV-1 entry and release (KCNJ2) and cell-cell communication (GJB2) in bystander cells (right) in an attempt to suppress persistent cell-cell infection during chronic HIV. Created with BioRender.com. (PDF) [file ppat.1011114.s011.pdf]
