# Supplementary material for: A cohort-based study of host gene expression: tumor suppressor and innate immune/inflammatory pathways associated with the HIV reservoir size
Source: PLoS Pathog. 2023 Nov 29;19(11):e1011114. doi: 10.1371/journal.ppat.1011114 (PMC10712869; doi:10.1371/journal.ppat.1011114)
Supplement: S1 Table — Genes sets with Benjamini-Hochberg false discovery rate (FDR)-adjusted q<0.25 are shown for the total study population (top panel) and for the European ancestry subgroup (bottom panel). Gene sets where q<0.05 are shown in bold font. (PDF) [file ppat.1011114.s012.pdf]

S1 Table. Gene set enrichment analyses (GSEA) of ranked differentially expressed genes in relation to HIV total DNA using the Gene Ontology Biological Processes (GO-BP) database. Genes sets with Benjamini-Hochberg false discovery rate (FDR)-adjusted q<0.25 are shown for the total study population (top panel) and for the European ancestry subgroup (bottom panel). Gene sets where q<0.05 are shown in bold font.

HIV Total DNA					
	**GO ID**	**Description**	**NES[a]**	**p[b]**	**q[c]**
**Total Study Population**					
1	GO:0036507	protein demannosylation	1.9	6.04E-05	0.171
2	GO:0036508	protein alpha-1,2-demannosylation	1.9	6.04E-05	0.171
3	GO:0006333	chromatin assembly or disassembly	1.4	1.00E-04	0.211
**European Ancestry Subgroup**					
1	GO:0006958	complement activation, classical pathway	1.8	2.26E-10	**1.27E-06**
2	GO:0030449	regulation of complement activation	1.9	3.50E-09	**9.80E-06**
3	GO:0006956	complement activation	1.7	6.65E-09	**1.24E-05**
4	GO:0002920	regulation of humoral immune response	1.8	8.98E-09	**1.26E-05**
5	GO:0002455	humoral immune response mediated by circulating immunoglobulin	1.7	1.44E-08	**1.61E-05**
6	GO:0006959	humoral immune response	1.5	9.50E-07	**9.00E-04**
7	GO:0019724	B cell mediated immunity	1.5	2.36E-06	**0.002**
8	GO:0016064	immunoglobulin mediated immune response	1.5	3.91E-06	**0.003**
9	GO:0002449	lymphocyte mediated immunity	1.4	4.74E-06	**0.003**
10	GO:0006910	phagocytosis, recognition	1.7	6.19E-06	**0.004**
11	GO:0006911	phagocytosis, engulfment	1.6	1.04E-05	**0.005**
12	GO:0002460	adaptive immune response based on somatic recombination of immune receptors built from immunoglobulin superfamily domains	1.4	1.66E-05	**0.008**
13	GO:0099024	plasma membrane invagination	1.6	4.18E-05	**0.018**
14	GO:0038094	Fc-gamma receptor signaling pathway	1.5	5.58E-05	**0.020**
15	GO:0002433	immune response-regulating cell surface receptor signaling pathway involved in phagocytosis	1.5	5.64E-05	**0.020**
16	GO:0038096	Fc-gamma receptor signaling pathway involved in phagocytosis	1.5	5.64E-05	**0.020**
17	GO:0042742	defense response to bacterium	1.4	8.86E-05	**0.029**
18	GO:0010324	membrane invagination	1.5	1.00E-04	**0.034**
19	GO:0002431	Fc receptor mediated stimulatory signaling pathway	1.5	1.00E-04	**0.040**
20	GO:0002377	immunoglobulin production	1.4	1.00E-04	**0.041**
21	GO:0070268	cornification	1.7	2.00E-04	0.066

22	GO:0043032	positive regulation of macrophage activation	1.8	6.00E-04	0.141
23	GO:0038095	Fc-epsilon receptor signaling pathway	1.4	9.00E-04	0.231
24	GO:0002440	production of molecular mediator of immune response	1.3	1.00E-03	0.238
25	GO:0002697	regulation of immune effector process	1.2	1.10E-03	0.245
26	GO:0010463	mesenchymal cell proliferation	1.7	1.20E-03	0.248

[a] NES = normalized enrichment score.
[b] p = two sided p-value.
[c] q = two-sided false discovery rate (FDR) Benjamini-Hochberg q-value.
