# Supplementary material for: A cohort-based study of host gene expression: tumor suppressor and innate immune/inflammatory pathways associated with the HIV reservoir size
Source: PLoS Pathog. 2023 Nov 29;19(11):e1011114. doi: 10.1371/journal.ppat.1011114 (PMC10712869; doi:10.1371/journal.ppat.1011114)
Supplement: S2 Table — (PDF) [file ppat.1011114.s013.pdf]

S2 Table. Multivariate models of P3H3 and NBL1 protein expression from peripheral CD4+ T cells in relation to HIV total DNA among 40 participants.

HIV Total DNA					
**Protein Name**	**Gene**	**p [a]**	**FC [b]**	**% Change [c]**	**Description**
NBL1, DAN Family BMP Antagonist	*NBL1*	0.060	0.988	-1.2%	NBL1, also known as neuroblastoma suppressor of tumorigenicity 1, is a transcription factor that belongs to the DAN (differential screening-selected gene aberrant in neuroblastoma) family of proteins [53, 54] and is involved in the negative regulation of cell cycle (G1/S transition) [55-58]. In a recent *ex vivo* analysis of CD4+ T cells from rhesus macaques after HIV-1 Env immunization and antibody co-administration, *NBL1* was identified as a host gene that was differentially expressed in all treated (CTLA-4, PD-1, and CTLA-4 + PD-1 Ab) versus control groups [106].
Prolyl 3-Hydroxylase 3	*P3H3*	0.163	0.988	-1.2%	*P3H3* encodes for Prolyl 3-Hydroxylase 3, which functions as a collagen prolyl hydroxylase (vital for collagen biosynthesis) that affects properties of the extracellular matrix and alters cellular behavior [46-49]. Prior studies suggest that P3H3 plays a role as a tumor suppressor in breast, lymphoid, and other cancers [50-52].

[a] p = two sided p-value.
[b] FC = fold-change in host protein expression per two-fold change in copies of HIV from multivariate model adjusted for nadir CD4+ T cell count and timing of ART initiation.
[c] % Change = percent change in host protein expression per two-fold change in copies of HIV.
