# Supplementary material for: A cohort-based study of host gene expression: tumor suppressor and innate immune/inflammatory pathways associated with the HIV reservoir size
Source: PLoS Pathog. 2023 Nov 29;19(11):e1011114. doi: 10.1371/journal.ppat.1011114 (PMC10712869; doi:10.1371/journal.ppat.1011114)
Supplement: S3 Table — (PDF) [file ppat.1011114.s014.pdf]

S3 Table. Differentially expressed host genes associated with HIV unspliced RNA (usRNA) in the total study population (top panel) and the European ancestry subgroup (bottom panel), at a Benjamini-Hochberg false discovery rate (FDR) of q<0.25, that were not shown in **Table 2**.

**HIV Unspliced RNA - Total Study Population**						
**Gene**	**Gene Name**	**p[a]**	**q[b]**	**FC[c]**	**% Change[d]**	**TPM[e]**
MAILR	Macrophage Interferon Regulatory Lncrna	4.75E-05	0.053	0.969	-3.1	0.38
PLA1A	Phospholipase A1 Member A	5.47E-05	0.057	0.946	-5.4	0.08
IL6	Interleukin 6	6.41E-05	0.062	0.926	-7.4	27.46
PTGES	Prostaglandin E Synthase	6.81E-05	0.062	0.932	-6.8	2.73
CLEC4D	C-Type Lectin Domain Family 4 Member D	6.82E-05	0.062	0.938	-6.2	1.02
C15orf48	Chromosome 15 Open Reading Frame 48	7.53E-05	0.063	0.943	-5.7	1.25
GBF1	Golgi Brefeldin A Resistant Guanine Nucleotide Exchange Factor 1	7.60E-05	0.063	1.005	0.5	6.71
CLEC4E	C-Type Lectin Domain Family 4 Member E	7.88E-05	0.063	0.927	-7.3	6.65
AL133163.2	Novel Transcript	8.68E-05	0.067	0.966	-3.4	0.33
CCRL2	C-C Motif Chemokine Receptor Like 2	9.33E-05	0.068	0.967	-3.3	4.29
SLC1A3	Solute Carrier Family 1 Member 3	9.63E-05	0.068	0.951	-4.9	0.02
SMPDL3A	Sphingomyelin Phosphodiesterase Acid Like 3A	9.90E-05	0.068	0.937	-6.3	1.10
CSPG4BP	Chondroitin Sulfate Proteoglycan Family Member 4B, Pseudogene	1.00E-04	0.072	0.952	-4.8	0.03
AQP9	Aquaporin 9	1.00E-04	0.072	0.925	-7.5	3.48
ADM	Adrenomedullin	1.00E-04	0.075	0.950	-5.0	25.45
TLR8	Toll Like Receptor 8	1.00E-04	0.086	0.933	-6.7	2.23
ACOD1	Aconitate Decarboxylase 1	2.00E-04	0.086	0.941	-5.9	0.29
LINC02605	Long Intergenic Non-Protein Coding RNA 2605	2.00E-04	0.086	0.952	-4.8	0.99
HMGB2	High Mobility Group Box 2	2.00E-04	0.086	1.007	0.7	676.34
FFAR2	Free Fatty Acid Receptor 2	2.00E-04	0.086	0.932	-6.8	6.29
SPHK1	Sphingosine Kinase 1	2.00E-04	0.086	0.966	-3.4	2.78
RIN2	Ras And Rab Interactor 2	2.00E-04	0.089	0.938	-6.2	0.36
F3	Coagulation Factor III, Tissue Factor	2.00E-04	0.097	0.940	-6.0	1.10
LINC01465	Long Intergenic Non-Protein Coding RNA 1465	2.00E-04	0.098	0.978	-2.2	14.95

PPP1R17	Protein Phosphatase 1 Regulatory Subunit 17	2.00E-04	0.101	0.954	-4.6	0.09
OSR2	Odd-Skipped Related Transciption Factor 2	2.00E-04	0.101	0.950	-5.0	2.35
AC025580.2	Novel Transcript, Antisense to SLC30A4	2.00E-04	0.101	0.950	-5.0	0.05
FPR2	Formyl Peptide Receptor 2	2.00E-04	0.101	0.936	-6.4	0.88
TBCC	Tubulin Folding Cofactor C	2.00E-04	0.102	1.010	1.0	534.08
VNN3	Vanin 3	2.00E-04	0.103	0.960	-4.0	1.44
AL391832.2	Novel Transcript	3.00E-04	0.111	0.958	-4.2	0.14
CLEC6A	C-Type Lectin Domain Containing 6A	3.00E-04	0.111	0.963	-3.7	0.05
AC254633.1	Novel Transcript	3.00E-04	0.111	1.037	3.7	7.81
C5orf58	Chromosome 5 Open Reading Frame 58	3.00E-04	0.111	0.984	-1.6	1.03
DRAM1	DNA Damage Regulated Autophagy Modulator 1	3.00E-04	0.111	0.970	-3.0	0.71
SLC22A15	Solute Carrier Family 22 Member 15	3.00E-04	0.111	0.962	-3.8	0.10
LINC01093	Long Intergenic Non-Protein Coding RNA 1093	3.00E-04	0.111	0.954	-4.6	0.39
ZMAT4	Zinc Finger Matrin-Type 4	3.00E-04	0.115	0.963	-3.7	8.62E-04
LSM12	LSM12 Homolog	3.00E-04	0.115	0.994	-0.6	5.44
VSTM1	V-Set And Transmembrane Domain Containing 1	3.00E-04	0.116	0.933	-6.7	0.23
CCL3	C-C Motif Chemokine Ligand 3	3.00E-04	0.116	0.944	-5.6	45.50
CLIC4	Chloride Intracellular Channel 4	4.00E-04	0.120	0.969	-3.1	1.33
PRLR	Prolactin Receptor	4.00E-04	0.120	0.952	-4.8	0.01
GPR84	G Protein-Coupled Receptor 84	4.00E-04	0.126	0.943	-5.7	8.56
TIGAR	TP53 Induced Glycolysis Regulatory Phosphatase	4.00E-04	0.126	0.988	-1.2	0.96
SFRP1	Secreted Frizzled Related Protein 1	4.00E-04	0.127	0.930	-7.0	0.07
IL1RN	Interleukin 1 Receptor Antagonist	4.00E-04	0.129	0.927	-7.3	10.00
RAB11FIP3	RAB11 Family Interacting Protein 3	4.00E-04	0.130	1.006	0.6	4.17
PDGFRB	Platelet Derived Growth Factor Receptor Beta	4.00E-04	0.131	0.952	-4.8	0.19
SLC9A7P1	Solute Carrier Family 9 Member 7 Pseudogene 1	4.00E-04	0.131	0.946	-5.4	2.14
TLR4	Toll Like Receptor 4	5.00E-04	0.136	0.935	-6.5	3.95
SLC39A1	Solute Carrier Family 39 Member 1	5.00E-04	0.141	0.973	-2.7	0.34
CYRIA	CYFIP Related Rac1 Interactor A	5.00E-04	0.141	0.960	-4.0	0.81
AC037198.1	Novel Transcript, Sense Intronic to THBS1	5.00E-04	0.141	0.958	-4.2	2.81
IGSF6	Immunoglobulin Superfamily Member 6	5.00E-04	0.142	0.981	-1.9	2.91

ZNF225	Zinc Finger Protein 225	5.00E-04	0.142	1.013	1.3	1.01
AC245884.11	Novel Transcript	5.00E-04	0.142	0.948	-5.2	0.27
VAMP2	Vesicle Associated Membrane Protein 2	5.00E-04	0.142	1.005	0.5	476.99
NINJ1	Ninjurin 1	5.00E-04	0.142	0.982	-1.8	77.28
CXCL1	C-X-C Motif Chemokine Ligand 1	5.00E-04	0.142	0.943	-5.7	8.10
KTI12	KTI12 Chromatin Associated Homolog	6.00E-04	0.144	1.009	0.9	47.62
FXYD6	FXYD Domain Containing Ion Transport Regulator 6	6.00E-04	0.145	0.952	-4.8	0.06
UICLM	Up-Regulated In Colorectal Cancer Liver Metastasis	6.00E-04	0.151	0.948	-5.2	0.24
ARRDC3	Arrestin Domain Containing 3	6.00E-04	0.154	1.013	1.3	210.43
FRMD7	FERM Domain Containing 7	6.00E-04	0.154	0.955	-4.5	0.02
MEFV	MEFV Innate Immuity Regulator, Pyrin	7.00E-04	0.156	0.946	-5.4	1.63
SPIB	Spi-B Transcription Factor	7.00E-04	0.156	0.954	-4.6	0.60
HCAR3	Hydroxycarboxylic Acid Receptor 3	7.00E-04	0.167	0.944	-5.6	10.86
MTCYBP23	MT-CYB Pseudogene 23	7.00E-04	0.168	0.975	-2.5	0.20
C1QA	Complement C1q A Chain	7.00E-04	0.169	0.944	-5.6	1.43
VCAM1	Vascular Cell Adhesion Molecule 1	8.00E-04	0.172	0.952	-4.8	0.14
AL117335.1	Novel Transcript, Antisense to SIRPA	8.00E-04	0.181	0.966	-3.4	0.12
BNC2	Basonuclin 2	8.00E-04	0.181	0.952	-4.8	0.01
IRAK2	Interleukin 1 Receptor Associated Kinase 2	8.00E-04	0.181	0.977	-2.3	1.46
AC112128.1	Novel Protein	8.00E-04	0.183	1.016	1.6	0.05
RPH3A	Rabphilin 3A	9.00E-04	0.189	0.950	-5.0	0.01
AC011990.1	Novel Transcript	9.00E-04	0.194	0.968	-3.2	0.01
NFKBIZ	NFKB Inhibitor Zeta	9.00E-04	0.195	0.983	-1.7	102.56
LINC02413	Long Intergenic Non-Protein Coding RNA 2413 (	9.00E-04	0.196	0.956	-4.4	0.04
MYO6	Myosin VI	1.00E-03	0.196	0.982	-1.8	0.33
AL353593.1	Novel Transcript, Antisense to OBSCN	1.00E-03	0.198	0.987	-1.3	0.51
AC079753.2	Novel Transcript	1.00E-03	0.202	0.957	-4.3	0.33
AC106881.1	Novel Transcript, Antisense to UNC5C	1.00E-03	0.202	0.967	-3.3	0.16
RNF144B	Ring Finger Protein 144B	1.00E-03	0.202	0.970	-3.0	1.88
ADPRH	ADP-Ribosylarginine Hydrolase	1.00E-03	0.202	0.981	-1.9	2.40
IFNB1	Interferon Beta 1	1.00E-03	0.203	0.950	-5.0	1.02
LPL	Lipoprotein Lipase	1.10E-03	0.205	0.956	-4.4	0.02
RPS15AP30	Ribosomal Protein S15a Pseudogene 30	1.10E-03	0.217	0.961	-3.9	3.34

SCARF1	Scavenger Receptor Class F Member 1	1.20E-03	0.222	0.980	-2.0	1.41
C4orf46	Chromosome 4 Open Reading Frame 46	1.20E-03	0.222	0.989	-1.1	17.30
AC096733.2	Novel Transcript	1.20E-03	0.222	0.953	-4.7	3.62
AC115618.1	Novel Transcript, Antisense to RBM3	1.20E-03	0.226	0.974	-2.6	2.58
TCF7L2	Transcription Factor 7 Like 2	1.30E-03	0.228	0.984	-1.6	0.27
CCL3L3	C-C Motif Chemokine Ligand 3 Like 3	1.30E-03	0.234	0.921	-7.9	110.30
CCL20	C-C Motif Chemokine Ligand 20	1.40E-03	0.246	0.965	-3.5	7.27
MAFF	MAF Bzip Transcription Factor F	1.40E-03	0.246	0.984	-1.6	33.57
KRT5	Keratin 5	1.40E-03	0.248	0.954	-4.6	0.35
**HIV Unspliced RNA – European Ancestry Subgroup**						
**Gene**	**Gene Name**	**p[a]**	**q[b]**	**FC[c]**	**% Change[d]**	
MTCYBP23	MT-CYB Pseudogene 23	1.88E-05	0.052	0.959	-4.1	0.2
ACOD1	Aconitate Decarboxylase 1	2.35E-05	0.057	0.919	-8.1	0.29
ZMAT4	Zinc Finger Matrin-Type 4	2.81E-05	0.060	0.941	-5.9	8.62E-04
IFNB1	Interferon Beta 1	3.35E-05	0.065	0.921	-7.9	1.02
MAILR	Macrophage Interferon Regulatory Lncrna	4.49E-05	0.079	0.958	-4.2	0.38
GHITM	Growth Hormone Inducible Transmembrane Protein	6.25E-05	0.096	0.993	-0.7	64.23
NBN	Nibrin	7.23E-05	0.096	0.989	-1.1	4.71
SFRP1	Secreted Frizzled Related Protein 1	7.46E-05	0.096	0.881	-11.9	0.07
KCNJ2-AS1	KCNJ2 Antisense RNA 1	7.48E-05	0.096	0.900	-10.0	5.2
DAPK1-IT1	DAPK1 Intronic Transcript 1	8.18E-05	0.099	0.933	-6.7	1.11
MMP2-AS1	MMP2 Antisense RNA 1	1.00E-04	0.116	0.937	-6.3	0.03
AC106712.1	Novel Transcript	1.00E-04	0.116	0.950	-5.0	0.02
LINC00211	Long Intergenic Non-Protein Coding RNA 211	1.00E-04	0.116	0.946	-5.4	0.01
CCRL2	C-C Motif Chemokine Receptor Like 2	1.00E-04	0.121	0.960	-4.0	4.29
RPL36AP16	Ribosomal Protein L36a Pseudogene 16	2.00E-04	0.152	1.030	3.0	19.28
CSPG4BP	Chondroitin Sulfate Proteoglycan Family Member 4B, Pseudogene	2.00E-04	0.164	0.934	-6.6	0.03
AC091173.1	Novel Transcript	2.00E-04	0.173	0.952	-4.8	0.15
SLC39A1	Solute Carrier Family 39 Member 1	2.00E-04	0.179	0.960	-4.0	0.34
TCF7L2	Transcription Factor 7 Like 2	2.00E-04	0.181	0.979	-2.1	0.27
ADPRH	ADP-Ribosylarginine Hydrolase	3.00E-04	0.189	0.973	-2.7	2.4
MIR3945HG	MIR3945 Host Gene	3.00E-04	0.189	0.935	-6.5	0.36
CSF3	Colony Stimulating Factor 3	3.00E-04	0.189	0.913	-8.7	8.04
BNC2	Basonuclin 2	3.00E-04	0.189	0.932	-6.8	0.01

AC096733.2	Novel Transcript	3.00E-04	0.189	0.929	-7.1	3.62
IL1A	Interleukin 1 Alpha	3.00E-04	0.191	0.898	-10.2	5.81
AC106881.1	Novel Transcript, Antisense to UNC5C	4.00E-04	0.215	0.954	-4.6	0.16
KTI12	KTI12 Chromatin Associated Homolog	4.00E-04	0.215	1.013	1.3	47.62
ZNF366	Zinc Finger Protein 366	4.00E-04	0.215	1.070	7.0	0.03
LINC00677	Long Intergenic Non-Protein Coding RNA 677	4.00E-04	0.215	0.952	-4.8	0.54
LPL	Lipoprotein Lipase	4.00E-04	0.218	0.940	-6.0	0.02
AC092723.1	Novel Transcript	4.00E-04	0.232	0.961	-3.9	0.06
AC011990.1	Novel Transcript	5.00E-04	0.232	0.955	-4.5	0.01
SLC22A15	Solute Carrier Family 22 Member 15	5.00E-04	0.234	0.951	-4.9	0.1
AC096667.1	Proline-Rich Protein 18-Like	5.00E-04	0.240	0.936	-6.4	0.72
RRN3P4	RRN3 Pseudogene 4	5.00E-04	0.240	0.942	-5.8	0.9
RNU1-103P	RNA, U1 Small Nuclear 103, Pseudogene	5.00E-04	0.240	0.976	-2.4	41.3
CXCL3	C-X-C Motif Chemokine Ligand 3	5.00E-04	0.240	0.915	-8.5	31.06
IMP3	IMP U3 Small Nucleolar Ribonucleoprotein 3	6.00E-04	0.248	1.013	1.3	14.31
KCNJ2	Potassium Inwardly Rectifying Channel Subfamily J Member 2	6.00E-04	0.248	0.906	-9.4	2.06
OTUD3	OTU Deubiquitinase 3	6.00E-04	0.248	0.990	-1.0	9.6
VNN3	Vanin 3 (Source:HGNC Symbol;Acc:HGNC:16431)	7.00E-04	0.248	0.955	-4.5	1.44
AC066616.2	Mitochondrially Encoded NADH 4L (MT-ND4L) Pseudogene	7.00E-04	0.248	0.967	-3.3	0.18
THBD	Thrombomodulin	7.00E-04	0.248	1.063	6.3	12.06
ZNF563	Zinc Finger Protein 563	7.00E-04	0.248	1.016	1.6	2.48
PRLR	Prolactin Receptor	7.00E-04	0.248	0.938	-6.2	0.01
TECPR2	Tectonin Beta-Propeller Repeat Containing 2	7.00E-04	0.248	0.990	-1.0	1.03
TUSC3	Tumor Suppressor Candidate 3	7.00E-04	0.248	1.094	9.4	1.75E-03

[a] p = two sided p-value.
[b] q = two-sided false discovery rate (FDR) Benjamini-Hochberg q-value.
[c] FC = fold-change in host gene expression per two-fold change in copies of HIV from multivariate model adjusted for age, sex, nadir CD4+ T cell count, timing of ART initiation, ancestry (PCs), and residual variability (probabilistic estimation of expression residuals, PEERs).
[d] % Change = percent change in host gene expression per two-fold change in copies of HIV.
[e] Mean transcripts per million.
