# Supplementary material for: A cohort-based study of host gene expression: tumor suppressor and innate immune/inflammatory pathways associated with the HIV reservoir size
Source: PLoS Pathog. 2023 Nov 29;19(11):e1011114. doi: 10.1371/journal.ppat.1011114 (PMC10712869; doi:10.1371/journal.ppat.1011114)
Supplement: S4 Table — Genes sets with Benjamini-Hochberg false discovery rate (FDR)-adjusted q<0.05 are shown for the total study population (top panel) and for the European ancestry subgroup (bottom panel). Gene sets where q<0.05 are shown in bold font. (PDF) [file ppat.1011114.s015.pdf]

S4 Table. Gene set enrichment analyses (GSEA) of ranked differentially expressed genes in relation to HIV unspliced RNA using the Gene Ontology Biological Processes (GO-BP) database. Genes sets with Benjamini-Hochberg false discovery rate (FDR)-adjusted q<0.05 are shown for the total study population (top panel) and for the European ancestry subgroup (bottom panel). Gene sets where q<0.05 are shown in bold font.

**HIV Unspliced RNA - Total Study Population**					
**Rank**	**GO ID**	**Description**	**NES[a]**	**p[b]**	**q[c]**
1	GO:0009617	response to bacterium	1.4	1.34E-08	**7.55E-05**
2	GO:0031663	lipopolysaccharide-mediated signaling pathway	1.8	2.05E-06	**0.006**
3	GO:0071222	cellular response to lipopolysaccharide	1.5	3.35E-06	**0.006**
4	GO:0032640	tumor necrosis factor production	1.6	4.39E-06	**0.006**
5	GO:0001818	negative regulation of cytokine production	1.4	4.87E-06	**0.006**
6	GO:0071706	tumor necrosis factor superfamily cytokine production	1.5	6.46E-06	**0.006**
7	GO:0032611	interleukin-1 beta production	1.6	9.80E-06	**0.008**
8	GO:0071219	cellular response to molecule of bacterial origin	1.5	1.34E-05	**0.008**
9	GO:0032652	regulation of interleukin-1 production	1.6	1.55E-05	**0.008**
10	GO:0032680	regulation of tumor necrosis factor production	1.5	1.61E-05	**0.008**
11	GO:0032651	regulation of interleukin-1 beta production	1.7	1.75E-05	**0.008**
12	GO:0071216	cellular response to biotic stimulus	1.4	1.75E-05	**0.008**
13	GO:1903555	regulation of tumor necrosis factor superfamily cytokine production	1.5	1.75E-05	**0.008**
14	GO:0002237	response to molecule of bacterial origin	1.4	2.08E-05	**0.008**
15	GO:0032496	response to lipopolysaccharide	1.4	2.42E-05	**0.009**
16	GO:0002697	regulation of immune effector process	1.3	3.17E-05	**0.011**
17	GO:0042108	positive regulation of cytokine biosynthetic process	1.7	4.35E-05	**0.014**
18	GO:0036230	granulocyte activation	1.3	4.52E-05	**0.014**
19	GO:0033002	muscle cell proliferation	1.5	5.81E-05	**0.017**
20	GO:0032612	interleukin-1 production	1.6	6.26E-05	**0.018**
21	GO:0032635	interleukin-6 production	1.5	7.16E-05	**0.019**
22	GO:0071396	cellular response to lipid	1.3	7.84E-05	**0.020**
23	GO:1904646	cellular response to amyloid-beta	1.8	8.29E-05	**0.020**
24	GO:0002275	myeloid cell activation involved in immune response	1.3	8.40E-05	**0.020**
25	GO:0006959	humoral immune response	1.4	9.76E-05	**0.022**
26	GO:0042119	neutrophil activation	1.3	1.00E-04	**0.022**
27	GO:0032649	regulation of interferon-gamma production	1.6	1.00E-04	**0.022**
28	GO:1904645	response to amyloid-beta	1.8	1.00E-04	**0.022**
29	GO:0002827	positive regulation of T-helper 1 type immune response	1.9	1.00E-04	**0.022**

30	GO:1904037	positive regulation of epithelial cell apoptotic process	1.7	1.00E-04	**0.022**
31	GO:0043312	neutrophil degranulation	1.3	1.00E-04	**0.022**
32	GO:0050688	regulation of defense response to virus	1.6	1.00E-04	**0.023**
33	GO:0060428	lung epithelium development	1.9	1.00E-04	**0.023**
34	GO:0002444	myeloid leukocyte mediated immunity	1.3	1.00E-04	**0.023**
35	GO:0002221	pattern recognition receptor signaling pathway	1.4	2.00E-04	**0.024**
36	GO:0050691	regulation of defense response to virus by host	1.7	2.00E-04	**0.024**
37	GO:0002446	neutrophil mediated immunity	1.3	2.00E-04	**0.024**
38	GO:0007249	I-kappaB kinase/NF-kappaB signaling	1.4	2.00E-04	**0.024**
39	GO:0038094	Fc-gamma receptor signaling pathway	1.4	2.00E-04	**0.024**
40	GO:0060740	prostate gland epithelium morphogenesis	2.0	2.00E-04	**0.025**
41	GO:0032755	positive regulation of interleukin-6 production	1.6	2.00E-04	**0.025**
42	GO:0002283	neutrophil activation involved in immune response	1.3	2.00E-04	**0.025**
43	GO:0002224	toll-like receptor signaling pathway	1.4	2.00E-04	**0.025**
44	GO:0042035	regulation of cytokine biosynthetic process	1.5	2.00E-04	**0.026**
45	GO:0032675	regulation of interleukin-6 production	1.5	2.00E-04	**0.031**
46	GO:0043299	leukocyte degranulation	1.3	2.00E-04	**0.031**
47	GO:0071674	mononuclear cell migration	1.6	3.00E-04	**0.032**
48	GO:0008544	epidermis development	1.4	3.00E-04	**0.033**
49	GO:0006909	phagocytosis	1.3	3.00E-04	**0.034**
50	GO:0032731	positive regulation of interleukin-1 beta production	1.6	3.00E-04	**0.037**
51	GO:0032653	regulation of interleukin-10 production	1.6	3.00E-04	**0.037**
52	GO:0043331	response to dsRNA	1.7	3.00E-04	**0.038**
53	GO:0001773	myeloid dendritic cell activation	1.8	4.00E-04	**0.040**
54	GO:0009913	epidermal cell differentiation	1.4	4.00E-04	**0.041**
55	GO:0032613	interleukin-10 production	1.6	4.00E-04	**0.041**
56	GO:0060512	prostate gland morphogenesis	1.8	4.00E-04	**0.042**
57	GO:0097028	dendritic cell differentiation	1.7	4.00E-04	**0.042**
58	GO:0030216	keratinocyte differentiation	1.4	4.00E-04	**0.042**
59	GO:0006898	receptor-mediated endocytosis	1.3	5.00E-04	**0.043**
60	GO:0032732	positive regulation of interleukin-1 production	1.6	5.00E-04	**0.043**
61	GO:0150077	regulation of neuroinflammatory response	1.8	5.00E-04	**0.043**
62	GO:0050663	cytokine secretion	1.4	5.00E-04	**0.043**
63	GO:0050900	leukocyte migration	1.2	5.00E-04	**0.043**
64	GO:0002431	Fc receptor mediated stimulatory signaling pathway	1.4	5.00E-04	**0.043**
65	GO:1903557	positive regulation of tumor necrosis factor superfamily cytokine production	1.5	5.00E-04	**0.043**
66	GO:0002460	adaptive immune response based on somatic recombination of immune receptors built from immunoglobulin superfamily domains	1.3	5.00E-04	**0.044**
67	GO:0002548	monocyte chemotaxis	1.6	5.00E-04	**0.044**
68	GO:0043588	skin development	1.4	5.00E-04	**0.044**

69	GO:0032760	positive regulation of tumor necrosis factor production	1.5	6.00E-04	**0.046**
70	GO:0050727	regulation of inflammatory response	1.3	6.00E-04	**0.046**
71	GO:0006816	calcium ion transport	1.3	6.00E-04	**0.046**
72	GO:0002433	immune response-regulating cell surface receptor signaling pathway involved in phagocytosis	1.4	6.00E-04	**0.046**
73	GO:0038096	Fc-gamma receptor signaling pathway involved in phagocytosis	1.4	6.00E-04	**0.046**
74	GO:0030595	leukocyte chemotaxis	1.4	6.00E-04	**0.047**
75	GO:0032602	chemokine production	1.5	6.00E-04	**0.049**
76	GO:0002819	regulation of adaptive immune response	1.4	7.00E-04	**0.049**

HIV Unspliced RNA - European Ancestry Subgroup

Rank	GO ID	Description	NES[a]	p[b]	q[c]
1	GO:0009617	response to bacterium	1.5	3.55E-12	**1.99E-08**
2	GO:0001819	positive regulation of cytokine production	1.5	1.97E-09	**5.50E-06**
3	GO:0032496	response to lipopolysaccharide	1.5	7.04E-09	**1.31E-05**
4	GO:0002237	response to molecule of bacterial origin	1.5	9.88E-09	**1.38E-05**
5	GO:0032103	positive regulation of response to external stimulus	1.4	2.48E-08	**2.78E-05**
6	GO:0031349	positive regulation of defense response	1.5	4.93E-08	**4.60E-05**
7	GO:0002699	positive regulation of immune effector process	1.5	3.24E-07	**3.00E-04**
8	GO:0023061	signal release	1.3	4.47E-07	**3.00E-04**
9	GO:0002694	regulation of leukocyte activation	1.3	1.24E-06	**8.00E-04**
10	GO:0050729	positive regulation of inflammatory response	1.6	1.43E-06	**8.00E-04**
11	GO:0071216	cellular response to biotic stimulus	1.5	1.72E-06	**9.00E-04**
12	GO:0050727	regulation of inflammatory response	1.4	1.91E-06	**9.00E-04**
13	GO:0002449	lymphocyte mediated immunity	1.4	2.94E-06	**0.001**
14	GO:0071222	cellular response to lipopolysaccharide	1.5	4.94E-06	**0.002**
15	GO:0022407	regulation of cell-cell adhesion	1.4	5.29E-06	**0.002**
16	GO:0071219	cellular response to molecule of bacterial origin	1.5	6.12E-06	**0.002**
17	GO:0002703	regulation of leukocyte mediated immunity	1.5	6.74E-06	**0.002**
18	GO:0002708	positive regulation of lymphocyte mediated immunity	1.6	7.29E-06	**0.002**
19	GO:0035747	natural killer cell chemotaxis	2.1	7.29E-06	**0.002**
20	GO:0050663	cytokine secretion	1.5	1.24E-05	**0.003**
21	GO:0035743	CD4-positive, alpha-beta T cell cytokine production	2.0	1.29E-05	**0.003**
22	GO:0050715	positive regulation of cytokine secretion	1.6	1.29E-05	**0.003**
23	GO:0002292	T cell differentiation involved in immune response	1.7	1.30E-05	**0.003**
24	GO:0045622	regulation of T-helper cell differentiation	1.9	1.31E-05	**0.003**
25	GO:0002367	cytokine production involved in immune response	1.6	1.34E-05	**0.003**
26	GO:0031663	lipopolysaccharide-mediated signaling pathway	1.7	1.61E-05	**0.004**
27	GO:0002705	positive regulation of leukocyte mediated immunity	1.5	1.77E-05	**0.004**
28	GO:1904646	cellular response to amyloid-beta	1.8	1.97E-05	**0.004**
29	GO:0002548	monocyte chemotaxis	1.8	2.00E-05	**0.004**
30	GO:0002706	regulation of lymphocyte mediated immunity	1.5	2.11E-05	**0.004**

31	GO:0002460	adaptive immune response based on somatic recombination of immune receptors built from immunoglobulin superfamily domains	1.3	2.16E-05	**0.004**
32	GO:0043370	regulation of CD4-positive, alpha-beta T cell differentiation	1.8	2.22E-05	**0.004**
33	GO:0055057	neuroblast division	2.1	2.61E-05	**0.004**
34	GO:0050905	neuromuscular process	1.7	2.69E-05	**0.004**
35	GO:0042093	T-helper cell differentiation	1.7	2.75E-05	**0.004**
36	GO:0050707	regulation of cytokine secretion	1.5	2.75E-05	**0.004**
37	GO:0043367	CD4-positive, alpha-beta T cell differentiation	1.6	2.89E-05	**0.004**
38	GO:0032649	regulation of interferon-gamma production	1.6	3.06E-05	**0.005**
39	GO:0070374	positive regulation of ERK1 and ERK2 cascade	1.5	3.17E-05	**0.005**
40	GO:0043410	positive regulation of MAPK cascade	1.3	3.96E-05	**0.006**
41	GO:0002718	regulation of cytokine production involved in immune response	1.6	4.29E-05	**0.006**
42	GO:0032755	positive regulation of interleukin-6 production	1.6	4.35E-05	**0.006**
43	GO:0050863	regulation of T cell activation	1.4	4.35E-05	**0.006**
44	GO:2000514	regulation of CD4-positive, alpha-beta T cell activation	1.7	4.46E-05	**0.006**
45	GO:0002286	T cell activation involved in immune response	1.6	4.74E-05	**0.006**
46	GO:0030593	neutrophil chemotaxis	1.6	4.74E-05	**0.006**
47	GO:0060191	regulation of lipase activity	1.6	4.80E-05	**0.006**
48	GO:0051249	regulation of lymphocyte activation	1.3	4.91E-05	**0.006**
49	GO:0061900	glial cell activation	1.7	4.91E-05	**0.006**
50	GO:0097529	myeloid leukocyte migration	1.4	4.91E-05	**0.006**
51	GO:0002720	positive regulation of cytokine production involved in immune response	1.7	5.53E-05	**0.006**
52	GO:0032635	interleukin-6 production	1.5	5.64E-05	**0.006**
53	GO:0060193	positive regulation of lipase activity	1.7	5.64E-05	**0.006**
54	GO:0060326	cell chemotaxis	1.4	5.70E-05	**0.006**
55	GO:0001906	cell killing	1.5	5.81E-05	**0.006**
56	GO:0042742	defense response to bacterium	1.4	6.26E-05	**0.006**
57	GO:0003229	ventricular cardiac muscle tissue development	1.8	6.37E-05	**0.006**
58	GO:0032609	interferon-gamma production	1.5	6.71E-05	**0.006**
59	GO:0051770	positive regulation of nitric-oxide synthase biosynthetic process	2.0	6.82E-05	**0.006**
60	GO:0042100	B cell proliferation	1.6	6.94E-05	**0.006**
61	GO:1903037	regulation of leukocyte cell-cell adhesion	1.4	6.94E-05	**0.006**
62	GO:0002526	acute inflammatory response	1.6	7.05E-05	**0.006**
63	GO:0007159	leukocyte cell-cell adhesion	1.3	7.05E-05	**0.006**
64	GO:0050867	positive regulation of cell activation	1.3	7.28E-05	**0.006**
65	GO:0032611	interleukin-1 beta production	1.6	7.39E-05	**0.006**
66	GO:0033002	muscle cell proliferation	1.4	7.50E-05	**0.006**
67	GO:0002697	regulation of immune effector process	1.3	7.61E-05	**0.006**

68	GO:0090179	planar cell polarity pathway involved in neural tube closure	2.0	7.61E-05	**0.006**
69	GO:0001505	regulation of neurotransmitter levels	1.4	7.95E-05	**0.006**
70	GO:1902106	negative regulation of leukocyte differentiation	1.6	7.95E-05	**0.006**
71	GO:0002369	T cell cytokine production	1.7	8.29E-05	**0.007**
72	GO:0042108	positive regulation of cytokine biosynthetic process	1.6	8.40E-05	**0.007**
73	GO:0032675	regulation of interleukin-6 production	1.5	9.19E-05	**0.007**
74	GO:0042116	macrophage activation	1.6	9.19E-05	**0.007**
75	GO:0002695	negative regulation of leukocyte activation	1.4	9.53E-05	**0.007**
76	GO:0001909	leukocyte mediated cytotoxicity	1.5	9.64E-05	**0.007**
77	GO:0070372	regulation of ERK1 and ERK2 cascade	1.4	9.76E-05	**0.007**
78	GO:0007249	I-kappaB kinase/NF-kappaB signaling	1.3	1.00E-04	**0.008**
79	GO:0001910	regulation of leukocyte mediated cytotoxicity	1.6	1.00E-04	**0.008**
80	GO:0001774	microglial cell activation	1.7	1.00E-04	**0.008**
81	GO:0002269	leukocyte activation involved in inflammatory response	1.7	1.00E-04	**0.008**
82	GO:0150076	neuroinflammatory response	1.6	1.00E-04	**0.008**
83	GO:0030595	leukocyte chemotaxis	1.4	1.00E-04	**0.009**
84	GO:0070371	ERK1 and ERK2 cascade	1.4	1.00E-04	**0.009**
85	GO:0002294	CD4-positive, alpha-beta T cell differentiation involved in immune response	1.6	1.00E-04	**0.009**
86	GO:0009615	response to virus	1.3	1.00E-04	**0.009**
87	GO:0031341	regulation of cell killing	1.5	1.00E-04	**0.009**
88	GO:0002456	T cell mediated immunity	1.5	1.00E-04	**0.009**
89	GO:0045620	negative regulation of lymphocyte differentiation	1.7	2.00E-04	**0.010**
90	GO:0032612	interleukin-1 production	1.5	2.00E-04	**0.010**
91	GO:0000186	activation of MAPKK activity	1.7	2.00E-04	**0.010**
92	GO:1905276	regulation of epithelial tube formation	1.9	2.00E-04	**0.011**
93	GO:0042330	taxis	1.2	2.00E-04	**0.011**
94	GO:0050708	regulation of protein secretion	1.3	2.00E-04	**0.011**
95	GO:0045089	positive regulation of innate immune response	1.4	2.00E-04	**0.012**
96	GO:0071396	cellular response to lipid	1.2	2.00E-04	**0.012**
97	GO:0097696	receptor signaling pathway via STAT	1.5	2.00E-04	**0.012**
98	GO:1902105	regulation of leukocyte differentiation	1.3	2.00E-04	**0.012**
99	GO:0031295	T cell costimulation	1.6	2.00E-04	**0.013**
100	GO:0035710	CD4-positive, alpha-beta T cell activation	1.5	2.00E-04	**0.013**
101	GO:0045581	negative regulation of T cell differentiation	1.7	3.00E-04	**0.014**
102	GO:0042089	cytokine biosynthetic process	1.5	3.00E-04	**0.015**
103	GO:0046688	response to copper ion	1.8	3.00E-04	**0.015**
104	GO:0002724	regulation of T cell cytokine production	1.7	3.00E-04	**0.015**
105	GO:0022409	positive regulation of cell-cell adhesion	1.4	3.00E-04	**0.015**
106	GO:0002287	alpha-beta T cell activation involved in immune response	1.6	3.00E-04	**0.015**
107	GO:0002293	alpha-beta T cell differentiation involved in immune response	1.6	3.00E-04	**0.015**

108	GO:0035745	T-helper 2 cell cytokine production	1.9	3.00E-04	**0.015**
109	GO:0042249	establishment of planar polarity of embryonic epithelium	1.9	3.00E-04	**0.015**
110	GO:0007259	receptor signaling pathway via JAK-STAT	1.5	3.00E-04	**0.015**
111	GO:0003401	axis elongation	1.8	3.00E-04	**0.016**
112	GO:0002696	positive regulation of leukocyte activation	1.3	3.00E-04	**0.017**
113	GO:0043331	response to dsRNA	1.7	3.00E-04	**0.017**
114	GO:0002430	complement receptor mediated signaling pathway	1.9	3.00E-04	**0.017**
115	GO:0042035	regulation of cytokine biosynthetic process	1.5	3.00E-04	**0.017**
116	GO:0042542	response to hydrogen peroxide	1.4	4.00E-04	**0.017**
117	GO:0051250	negative regulation of lymphocyte activation	1.4	4.00E-04	**0.018**
118	GO:0071692	protein localization to extracellular region	1.3	4.00E-04	**0.018**
119	GO:0006935	chemotaxis	1.2	4.00E-04	**0.018**
120	GO:0002791	regulation of peptide secretion	1.3	4.00E-04	**0.018**
121	GO:0032642	regulation of chemokine production	1.6	4.00E-04	**0.018**
122	GO:0045624	positive regulation of T-helper cell differentiation	1.8	4.00E-04	**0.019**
123	GO:0032613	interleukin-10 production	1.6	4.00E-04	**0.020**
124	GO:2000551	regulation of T-helper 2 cell cytokine production	1.9	4.00E-04	**0.020**
125	GO:0018212	peptidyl-tyrosine modification	1.3	5.00E-04	**0.020**
126	GO:0055010	ventricular cardiac muscle tissue morphogenesis	1.7	5.00E-04	**0.020**
127	GO:0002830	positive regulation of type 2 immune response	1.8	5.00E-04	**0.021**
128	GO:0034341	response to interferon-gamma	1.4	5.00E-04	**0.021**
129	GO:0042088	T-helper 1 type immune response	1.6	5.00E-04	**0.021**
130	GO:0006836	neurotransmitter transport	1.3	5.00E-04	**0.021**
131	GO:0031294	lymphocyte costimulation	1.6	5.00E-04	**0.021**
132	GO:0032602	chemokine production	1.5	5.00E-04	**0.021**
133	GO:0010518	positive regulation of phospholipase activity	1.6	5.00E-04	**0.021**
134	GO:0002833	positive regulation of response to biotic stimulus	1.3	5.00E-04	**0.021**
135	GO:0090177	establishment of planar polarity involved in neural tube closure	1.9	5.00E-04	**0.021**
136	GO:0090178	regulation of establishment of planar polarity involved in neural tube closure	1.9	5.00E-04	**0.021**
137	GO:0043330	response to exogenous dsRNA	1.7	5.00E-04	**0.021**
138	GO:1990266	neutrophil migration	1.5	5.00E-04	**0.021**
139	GO:0000302	response to reactive oxygen species	1.3	5.00E-04	**0.022**
140	GO:0050731	positive regulation of peptidyl-tyrosine phosphorylation	1.4	6.00E-04	**0.023**
141	GO:0002573	myeloid leukocyte differentiation	1.4	6.00E-04	**0.023**
142	GO:1904407	positive regulation of nitric oxide metabolic process	1.7	6.00E-04	**0.023**
143	GO:0050870	positive regulation of T cell activation	1.3	6.00E-04	**0.024**
144	GO:0042107	cytokine metabolic process	1.5	6.00E-04	**0.024**
145	GO:0036005	response to macrophage colony-stimulating factor	1.9	6.00E-04	**0.024**
146	GO:0036006	cellular response to macrophage colony-stimulating factor stimulus	1.9	6.00E-04	**0.024**
147	GO:0032653	regulation of interleukin-10 production	1.6	6.00E-04	**0.024**

148	GO:0002702	positive regulation of production of molecular mediator of immune response	1.5	6.00E-04	**0.024**
149	GO:0032634	interleukin-5 production	1.9	6.00E-04	**0.024**
150	GO:0032674	regulation of interleukin-5 production	1.9	6.00E-04	**0.024**
151	GO:0050864	regulation of B cell activation	1.4	6.00E-04	**0.024**
152	GO:0046637	regulation of alpha-beta T cell differentiation	1.6	7.00E-04	**0.025**
153	GO:0050869	negative regulation of B cell activation	1.7	7.00E-04	**0.027**
154	GO:0032722	positive regulation of chemokine production	1.6	8.00E-04	**0.027**
155	GO:0002521	leukocyte differentiation	1.2	8.00E-04	**0.027**
156	GO:0032943	mononuclear cell proliferation	1.3	8.00E-04	**0.027**
157	GO:0051251	positive regulation of lymphocyte activation	1.3	8.00E-04	**0.027**
158	GO:1904645	response to amyloid-beta	1.7	8.00E-04	**0.027**
159	GO:0002822	regulation of adaptive immune response based on somatic recombination of immune receptors built from immunoglobulin superfamily domains	1.4	8.00E-04	**0.029**
160	GO:0048660	regulation of smooth muscle cell proliferation	1.4	8.00E-04	**0.029**
161	GO:0048659	smooth muscle cell proliferation	1.4	8.00E-04	**0.030**
162	GO:0042110	T cell activation	1.2	9.00E-04	**0.030**
163	GO:0050868	negative regulation of T cell activation	1.4	9.00E-04	**0.030**
164	GO:0070661	leukocyte proliferation	1.3	9.00E-04	**0.030**
165	GO:0002790	peptide secretion	1.2	9.00E-04	**0.030**
166	GO:0009306	protein secretion	1.2	9.00E-04	**0.030**
167	GO:0035592	establishment of protein localization to extracellular region	1.2	9.00E-04	**0.030**
168	GO:0042267	natural killer cell mediated cytotoxicity	1.5	9.00E-04	**0.030**
169	GO:0002711	positive regulation of T cell mediated immunity	1.5	9.00E-04	**0.030**
170	GO:0042269	regulation of natural killer cell mediated cytotoxicity	1.6	9.00E-04	**0.030**
171	GO:0043371	negative regulation of CD4-positive, alpha-beta T cell differentiation	1.7	9.00E-04	**0.030**
172	GO:0002228	natural killer cell mediated immunity	1.5	1.00E-03	**0.031**
173	GO:0002700	regulation of production of molecular mediator of immune response	1.4	1.00E-03	**0.031**
174	GO:0031343	positive regulation of cell killing	1.5	1.00E-03	**0.031**
175	GO:0002438	acute inflammatory response to antigenic stimulus	1.8	1.00E-03	**0.031**
176	GO:0002819	regulation of adaptive immune response	1.4	1.00E-03	**0.031**
177	GO:0032623	interleukin-2 production	1.5	1.00E-03	**0.031**
178	GO:0032651	regulation of interleukin-1 beta production	1.5	1.00E-03	**0.032**
179	GO:0032731	positive regulation of interleukin-1 beta production	1.6	1.00E-03	**0.032**
180	GO:0046632	alpha-beta T cell differentiation	1.4	1.00E-03	**0.032**
181	GO:0045217	cell-cell junction maintenance	1.8	1.00E-03	**0.032**
182	GO:0018108	peptidyl-tyrosine phosphorylation	1.3	1.10E-03	**0.033**
183	GO:0070664	negative regulation of leukocyte proliferation	1.5	1.10E-03	**0.033**
184	GO:0010035	response to inorganic substance	1.2	1.10E-03	**0.033**
185	GO:0046651	lymphocyte proliferation	1.3	1.10E-03	**0.033**
186	GO:2000319	regulation of T-helper 17 cell differentiation	1.7	1.10E-03	**0.034**

187	GO:0050866	negative regulation of cell activation	1.3	1.20E-03	**0.036**
188	GO:0002673	regulation of acute inflammatory response	1.6	1.20E-03	**0.036**
189	GO:0043122	regulation of I-kappaB kinase/NF-kappaB signaling	1.3	1.20E-03	**0.036**
190	GO:0061081	positive regulation of myeloid leukocyte cytokine production involved in immune response	1.7	1.20E-03	**0.036**
191	GO:0032729	positive regulation of interferon-gamma production	1.5	1.20E-03	**0.036**
192	GO:0032740	positive regulation of interleukin-17 production	1.8	1.20E-03	**0.036**
193	GO:0032732	positive regulation of interleukin-1 production	1.5	1.30E-03	**0.037**
194	GO:0050900	leukocyte migration	1.2	1.30E-03	**0.037**
195	GO:0051047	positive regulation of secretion	1.2	1.30E-03	**0.038**
196	GO:0002431	Fc receptor mediated stimulatory signaling pathway	1.4	1.40E-03	**0.040**
197	GO:0046634	regulation of alpha-beta T cell activation	1.4	1.40E-03	**0.040**
198	GO:0007269	neurotransmitter secretion	1.4	1.40E-03	**0.040**
199	GO:0099643	signal release from synapse	1.4	1.40E-03	**0.040**
200	GO:0030889	negative regulation of B cell proliferation	1.7	1.50E-03	**0.042**
201	GO:0001912	positive regulation of leukocyte mediated cytotoxicity	1.5	1.50E-03	**0.042**
202	GO:0002715	regulation of natural killer cell mediated immunity	1.6	1.50E-03	**0.042**
203	GO:0030888	regulation of B cell proliferation	1.5	1.50E-03	**0.042**
204	GO:0043405	regulation of MAP kinase activity	1.3	1.60E-03	**0.043**
205	GO:0045429	positive regulation of nitric oxide biosynthetic process	1.6	1.60E-03	**0.044**
206	GO:0050885	neuromuscular process controlling balance	1.6	1.60E-03	**0.044**
207	GO:2000379	positive regulation of reactive oxygen species metabolic process	1.5	1.60E-03	**0.044**
208	GO:0060026	convergent extension	1.8	1.60E-03	**0.044**
209	GO:0033674	positive regulation of kinase activity	1.2	1.70E-03	**0.044**
210	GO:0038094	Fc-gamma receptor signaling pathway	1.4	1.70E-03	**0.044**
211	GO:0050829	defense response to Gram-negative bacterium	1.6	1.70E-03	**0.045**
212	GO:0050730	regulation of peptidyl-tyrosine phosphorylation	1.3	1.70E-03	**0.045**
213	GO:1903426	regulation of reactive oxygen species biosynthetic process	1.5	1.70E-03	**0.045**
214	GO:1901224	positive regulation of NIK/NF-kappaB signaling	1.4	1.70E-03	**0.045**
215	GO:2000516	positive regulation of CD4-positive, alpha-beta T cell activation	1.6	1.70E-03	**0.045**
216	GO:0002709	regulation of T cell mediated immunity	1.5	1.80E-03	**0.045**
217	GO:0002824	positive regulation of adaptive immune response based on somatic recombination of immune receptors built from immunoglobulin superfamily domains	1.4	1.80E-03	**0.045**
218	GO:0060740	prostate gland epithelium morphogenesis	1.8	1.80E-03	**0.045**
219	GO:0097191	extrinsic apoptotic signaling pathway	1.3	1.80E-03	**0.045**
220	GO:0030101	natural killer cell activation	1.5	1.80E-03	**0.045**
221	GO:0045619	regulation of lymphocyte differentiation	1.3	1.80E-03	**0.045**
222	GO:1904892	regulation of receptor signaling pathway via STAT	1.4	1.80E-03	**0.045**
223	GO:0006450	regulation of translational fidelity	1.7	1.90E-03	**0.047**

224	GO:0034614	cellular response to reactive oxygen species	1.3	1.90E-03	**0.048**

[a] NES = normalized enrichment score.
[b] p = two sided p-value.
[c] q = two-sided false discovery rate (FDR) Benjamini-Hochberg q-value.
