# Supplementary material for: A cohort-based study of host gene expression: tumor suppressor and innate immune/inflammatory pathways associated with the HIV reservoir size
Source: PLoS Pathog. 2023 Nov 29;19(11):e1011114. doi: 10.1371/journal.ppat.1011114 (PMC10712869; doi:10.1371/journal.ppat.1011114)
Supplement: S5 Table — (PDF) [file ppat.1011114.s016.pdf]

S5 Table. Multivariate models of plasma IL-1β, IL-10, TNF-α, G-CSF, IP-10, TNFAIP5, and sTLR protein expression in relation to HIV unspliced RNA among 175 participants.

HIV Unspliced RNA					
**Protein Name**	**Gene**	**p[a]**	**FC[b]**	**% Change[c]**	**Description**
Interleukin-1 alpha (IL-1α) [d]	*IL1A* [d]	--	--	--	IL-1α is one of 11 members of the IL-1 family of cytokines [189]. The IL-1 cytokine family that as damage-associated molecular patterns (DAMPs) triggering innate inflammation and also play a key role in angiogenesis, along with tumor necrosis factor and IL-6 [190]. IL-1α is constitutively present intracellularly in most non-hematopoietic cells, rarely secreted by living cells and in contrast to IL-1β (the most well studied cytokine in this family), IL-1α can act as a “dual function” cytokine, serving as both an alarmin (directly senses DNA damage) as well as a proinflammatory mediator [78, 79].
Interleukin-1 beta (IL-1β)	*IL1B*	0.109	0.870	-13.0%	IL-1β is the primary circulating form of IL-1 [107] and is a potent proinflammatory cytokine that is upregulated in several disease states [158]. Like IL-1α, IL-1β induces inflammation by triggering a cascade of inflammatory mediators via the NOD-like receptor family pyrin domain containing 3 (NRLP3) inflammasome pathway [81, 82]. IL-1 is an “upstream” pro-inflammatory inducer of interleukin-6 (IL-6) [83], and plasma IL-6 strongly predicts morbidity (e.g., myocardial infarction, stroke, malignancy) [84-87] and mortality [80, 86-89] among people with HIV on ART. The IL-1 signaling pathway, and in particular, IL-1β, has emerged as a major target for immune modulation [107, 159].
Interleukin-10 (IL-10)	*IL10*	0.365	0.971	-2.9%	IL-10 is a pleiotropic cytokine that is produced by, and can target, many cell types (e.g., CD4+ and CD8+ T cells, B cells, macrophages, dendritic cells, natural killer cells) and plays a critical role in balancing anti- and pro- inflammatory immunity and tissue protection/mucosal homeostasis [142]. The effect of IL-10 varies by stage of infection and by tissue [139-141]. While IL-10 is mostly an anti-inflammatory cytokine widely known for preventing excessive inflammation in immune-mediated diseases such as inflammatory bowel disease [143, 144], rheumatoid arthritis systemic lupus erythematosus [145], and SARS-CoV-2 infection [146-148], and most recently, related to the maintenance of the SIV reservoir during ART [75], IL-10 has been shown to exert potent innate immune defenses (e.g., in bacterial sepsis [141]).
Tumor necrosis factor alpha (TNF-α)	*TNFA*	4.51e-3	0.798	-20.2%	TNF-α is a multifunctional cytokine that plays critical roles in innate and adaptive immunity, normal physiologic functions of immune cells, and has been associated with pathogenic inflammatory diseases and metabolic disorders [152]. TNF-α-induced PRRs such as TNFAIP5, TNFAIP6, and TNFAIP9 not only respond to TNF-α, they are modulated by the NF-κB pathway , respond to toll-like receptor engagement and IL-1 signaling (upon bacterial lipopolysaccharide stimulation) [116-119], and

					negatively regulate NF-κB signaling and IL-6 production [150, 151]. Thus, TNF plays a key role in proinflammatory responses linked to IL-1β and IL-6 signaling [153] and has been long been recognized to be associated with inflammatory [154] and infectious diseases such as HIV [155].
Colony Stimulating Factor 3 (G-CSF)	*CSF3*	0.410	1.014	1.5%	*CSF3* encodes for granulocyte stimulating factor 3 (G-CSF), a member of the IL-6 superfamily of cytokines [115]. G-CSF is mostly known for its role as a growth factor for neutrophils, promoting the proliferation and survival of neutrophil precursors. However, G-CSF has also been shown to regulate T cell responses via induction of IL-10 secretion [167], leading to inhibition of CD4+ and CD8+ T cell responses and reduction of cytotoxic responses [168]. Indeed, donor treatment of pegylated G-CSF was found to increase IL-10-producing regulatory T cells (Tregs) and enhance transplant tolerance [169], suggesting that G-CSF can directly modify T cell responses via IL-10 [196].
Interferon gamma-induced protein 10 (IP-10)	*CXCL10*	0.250	1.026	2.6%	*CXCL10* encodes for IP-10 (interferon gamma-induced protein 10) which recruits activated Th1 lymphocytes to sites of infection [120-122] and in HIV, signals through TLR7/9-dependent pathways [122], predicts HIV disease progression [123, 124], correlates with acute HIV seroconversion [125], and promotes HIV latency [126, 127].
Tumor Necrosis Factor-α induced protein 5 (TNFAIP5), Pentraxin-related protein (PTX3), Tumor Necrosis Factor-Inducible Gene 14 Protein (TSFG14)	*TNFAIP5*	0.584	1.01	1.4%	*TNFAIP5* encodes for a pattern recognition receptor, Tumor Necrosis Factor-α induced protein 5, also known as pentraxin-related protein (PTX3) or Tumor Necrosis Factor-Inducible Gene 14 Protein (TSFG14). TNFAIP5, or PTX3, is induced in response to TNF-α, TLR engagement and IL-1β signaling and is part of the pentraxin superfamily of proteins, which includes C-reactive protein (CRP) and serum amyloid [209]. TNFAIP5 is a soluble PRR that plays a key role in host antimicrobial innate immune defense but also functions in complement activation and regulating inflammation in response to tissue repair and cancer [210]. Elevated serum levels of PTX have been associated with severity and survival in patients with sepsis [211].
Toll-like receptor 4 (TLR4)	*TLR4*	0.863	0.997	-0.3%	TLR4 encodes for toll-like receptor (TLR) 4, which plays an important role in recognizing bacterial lipopolysaccharide (LPS) and inducing proinflammatory signaling such as the NF-kB and IL-1 pathways [163]. Indeed HIV-1 Tat has been shown to induce TLR4-induced LPS-mediated cytokine release in the gastrointestinal system [164] and may support several studies demonstrating higher levels of bacterial translocation in people with HIV compared to uninfected individuals [133-138].

[a] p = two sided p-value.
[b] FC = fold-change in host protein expression per two-fold change in copies of HIV from multivariate model adjusted for nadir CD4+ T cell count and timing of ART initiation.
[c] % Change = percent change in host protein expression per two-fold change in copies of HIV
[d] To maximize the sensitivity in the detection of IL-1α, which is constitutively present intracellularly in most non-hematopoietic cells [78, 79] but rarely secreted by living cells [80, 81], we used two technical replicates per undiluted sample and incubation was performed with agitation of the plates overnight at 4˚C. However, we were able to detect only 13 out of the 175 samples tested (7.4%), and the concentration of these samples were below the detection range (0.01-0.09 pg/ml), rendering the calculations less reliable, and thus results for IL-1α are not shown in final tables.
