# Supplementary material for: A cohort-based study of host gene expression: tumor suppressor and innate immune/inflammatory pathways associated with the HIV reservoir size
Source: PLoS Pathog. 2023 Nov 29;19(11):e1011114. doi: 10.1371/journal.ppat.1011114 (PMC10712869; doi:10.1371/journal.ppat.1011114)
Supplement: S6 Table — (PDF) [file ppat.1011114.s017.pdf]

S6 Table. Multivariate models of Kir2.1 (*KCNJ2*) and Connexin 26 (*GJB2*) protein expression from peripheral CD4+ T cells in relation to HIV unspliced RNA among 40 participants.

HIV Unspliced RNA					
**Protein Name**	**Gene**	**p [a]**	**FC[b]**	**% Change[c]**	**Description**
Kir2.1	*KCNJ2*	0.570	0.997	-1.6%	Inwardly rectifying potassium channel Kir2.1 is the encoded protein for *KCNJ2*. Inwardly rectifying potassium ion channels can regulate HIV-1 entry and release into host cells [82]. Tight regulation of potassium ion concentrations has been shown to play a critical role in HIV-1 virus production in CD4+ T cells in cell culture models [170]. HIV Nef protein has been shown to increase K+ concentrations in cells [171], and in turn, changes in K+ concentration have been shown to regulate stages in the HIV life cycle (viral entry, replication, and release) [82].
Connexin 26	*GJB2*	0.858	0.998	-0.2%	Gap junction beta 2 protein, also known as connexin 26, is the encoded protein for *GJB2*, also known as *CX26*. Gap junction proteins, or connexins, act as cell-cell communication channels to transport signaling molecules (e.g., K^+, Ca+, ATP) [83, 84], but HIV-1 has been shown to exploit these communication channels to disseminate infection as well as associated inflammation even in the absence of viral replication [85, 86]. Connexins are expressed in the endoplasmic reticulum and transported to the plasma membrane as connexin hemichannels that then fuse apposing cells, forming gap junctions [191, 192]. A growing body of literature strongly suggests that connexins intensify inflammation by facilitating damage-associated molecular pattern (DAMP) release, which then bind to pattern recognition receptors such as toll-like receptors (TLRs) and nod-like receptors (NLRs). Thus, in several inflammatory diseases, blocking connexin channels has consistently been shown to reduce tissue injury and improve organ function [172].

[a] p = two sided p-value.
[b] FC = fold-change in host protein expression per two-fold change in copies of HIV from multivariate model adjusted for nadir CD4+ T cell count and timing of ART initiation.
[c] % Change = percent change in host protein expression per two-fold change in copies of HIV.
