# Supplementary material for: A cohort-based study of host gene expression: tumor suppressor and innate immune/inflammatory pathways associated with the HIV reservoir size
Source: PLoS Pathog. 2023 Nov 29;19(11):e1011114. doi: 10.1371/journal.ppat.1011114 (PMC10712869; doi:10.1371/journal.ppat.1011114)
Supplement: S7 Table — Differentially expressed host genes in relation to HIV Intact DNA in the Total Study Population (top panel) and the European ancestry subgroup (bottom panel), at a Benjamini-Hochberg false discovery rate (FDR) of q<0.25. Two-fold higher level of HIV intact DNA was associated with upregulation of genes involved in glycogen degradation (AGL) and inhibits thrombus (clot) degradation (PLGLB1). (PDF) [file ppat.1011114.s018.pdf]

S7 Table. Differentially expressed host genes in relation to HIV Intact DNA in the Total Study Population (top panel) and the European ancestry subgroup (bottom panel), at a Benjamini-Hochberg false discovery rate (FDR) of q<0.25. Two-fold higher level of HIV intact DNA was associated with upregulation of genes involved in glycogen degradation (*AGL*) and inhibits thrombus (clot) degradation (*PLGLB1*).

**HIV Intact DNA**							
**Gene**	**Gene Name**	**p[a]**	**q[b]**	**FC[c]**	**% Change[d]**	**TPM[e]**	**Description**
**Total Study Population**							
NA							
**European Ancestry Subgroup**							
AGL	amylo-alpha-1, 6-glucosidase, 4-alpha-glucanotransferase	2.09E-05	0.23	1.009	0.9	3.36	*AGL* (AGL, Amylo-Alpha-1, 6-Glucosidase, 4-Alpha-Glucanotransferase) gene is involved in glycogen metabolism (process of breaking down stored glucose into glucose molecules for immediate glucose release and availability) [213-215].
PLGLB1	plasminogen like B1	2.39E-05	0.23	1.060	6.0	0.31	*PLGLB1 (*plasminogen like B1) gene is involved in thrombin clot degradation) [216-218]. PLGLB1 has previously been linked to a clonally expanded HIV-1 provirus (integrated in the opposite direction) from a patient with squamous cell carcinoma [97].

[a] p = two sided p-value.
[b] q = two-sided false discovery rate (FDR) Benjamini-Hochberg q-value. Bold font denotes genes with q<0.05.
[c] FC = fold-change in host gene expression per two-fold change in copies of HIV from multivariate model adjusted for nadir CD4+ T cell count, timing of ART initiation, ancestry (PCs), and residual variability (probabilistic estimation of expression residuals, PEERs).
[d] % Change = percent change in host gene expression per two-fold change in copies of HIV.
[e] Mean transcripts per million.
