# Supplementary material for: A cohort-based study of host gene expression: tumor suppressor and innate immune/inflammatory pathways associated with the HIV reservoir size
Source: PLoS Pathog. 2023 Nov 29;19(11):e1011114. doi: 10.1371/journal.ppat.1011114 (PMC10712869; doi:10.1371/journal.ppat.1011114)
Supplement: S8 Table — Genes sets with Benjamini-Hochberg false discovery rate (FDR)-adjusted q<0.25 are shown for the total study population (top panel) and for the European ancestry subgroup (bottom panel). Gene sets where q<0.05 are shown in bold font. (PDF) [file ppat.1011114.s019.pdf]

S8 Table. Gene set enrichment analyses (GSEA) of ranked differentially expressed genes in relation to HIV intact DNA using the Gene Ontology Biological Processes (GO-BP) database. Genes sets with Benjamini-Hochberg false discovery rate (FDR)-adjusted q<0.25 are shown for the total study population (top panel) and for the European ancestry subgroup (bottom panel). Gene sets where q<0.05 are shown in bold font.

**HIV Intact DNA**					
	**GO ID**	**Description**	**NES[a]**	**p[b]**	**q[c]**
**Total Study Population**					
NA					
**European Ancestry Subgroup**					
1	GO:0043312	neutrophil degranulation	1.3	1.49E-05	**0.046**
2	GO:0002283	neutrophil activation involved in immune response	1.3	1.91E-05	**0.046**
3	GO:0043299	leukocyte degranulation	1.3	2.47E-05	**0.046**
4	GO:0002444	myeloid leukocyte mediated immunity	1.3	4.13E-05	0.058
5	GO:0002275	myeloid cell activation involved in immune response	1.3	6.15E-05	0.060
6	GO:0002446	neutrophil mediated immunity	1.3	6.49E-05	0.060
7	GO:0042119	neutrophil activation	1.3	7.50E-05	0.060
8	GO:0036230	granulocyte activation	1.3	1.00E-04	0.085
9	GO:0019377	glycolipid catabolic process	1.9	3.00E-04	0.157
10	GO:0016311	dephosphorylation	1.3	3.00E-04	0.162
11	GO:0046479	glycosphingolipid catabolic process	1.9	4.00E-04	0.205
12	GO:0060037	pharyngeal system development	1.9	5.00E-04	0.224

[a] NES = normalized enrichment score.
[b] p = two sided p-value.
[c] q = two-sided false discovery rate (FDR) Benjamini-Hochberg q-value.
